# Brain-targeted liposomes loaded with monoclonal antibodies reduce alpha-synuclein aggregation and improve behavioral symptoms in Parkinson’s disease

**DOI:** 10.1002/adma.202304654

**Published:** 2023-09-27

**Authors:** Mor Sela, Maria Poley, Patricia Mora-Raimundo, Shaked Kagan, Aviram Avital, Maya Kaduri, Gal Chen, Omer Adir, Adi Rozencweig, Yfat Weiss, Ofir Sade, Yael Leichtmann-Bardoogo, Lilach Simchi, Shlomit Aga-Mizrachi, Batia Bell, Yoel Yeretz-Peretz, Aviv Zaid Or, Ashwani Choudhary, Idan Rosh, Diogo Cordeiro, Stav Cohen-Adiv, Yevgeny Berdichevsky, Anas Odeh, Jeny Shklover, Janna Shainsky-Roitman, Joshua E. Schroeder, Dov Hershkovitz, Peleg Hasson, Avraham Ashkenazi, Shani Stern, Tal Laviv, Ayal Ben-Zvi, Avi Avital, Uri Ashery, Ben M. Maoz, Avi Schroeder

**Affiliations:** 1 The Louis Family Laboratory for Targeted Drug Delivery and Personalized Medicine Technologies, Department of Chemical Engineering, Technion – Israel Institute of Technology, Haifa 32000, Israel; 2 The Norman Seiden Multidisciplinary Program for Nanoscience and Nanotechnology, Technion – Israel Institute of Technology, Haifa 32000, Israel; 3 The Interdisciplinary Program for Biotechnology, Technion - Israel Institute of Technology, Haifa 32000, Israel; 4 School of Neurobiology, Biochemistry and Biophysics, George S. Wise Faculty of Life Sciences, Sagol School of Neuroscience, Tel Aviv University, Tel Aviv 6997801, Israel; 5 Sagol School of Neuroscience, Tel Aviv University, Tel Aviv 6997801, Israel; 6 Department of Occupational Therapy, Faculty of Social Welfare and Health Sciences, University of Haifa, Haifa 3498838, Israel; 7 Department of Physiology and Pharmacology, Faculty of Medicine, Tel Aviv University, Tel Aviv 6997801, Israel; 8 Department of Developmental Biology and Cancer Research, The Institute for Medical Research Israel-Canada, Faculty of Medicine, The Hebrew University of Jerusalem, Jerusalem 9190500, Israel; 9 Sagol Department of Neurobiology, Faculty of Natural Sciences, University of Haifa, Haifa 3498838, Israel; 10 The Department of Cell and Developmental Biology, Faculty of Medicine, Tel Aviv University, Tel Aviv 6997801, Israel; 11 Spine Unit, Orthopedic Complex, Hadassah Hebrew University Medical Center, Kiryat Hadassah, POB 12000, Jerusalem 9190500, Israel; 12 Department of Pathology, Tel Aviv Sourasky Medical Center, Tel Aviv 6997801, Israel; 13 Sackler Faculty of Medicine, Tel Aviv University, Tel Aviv 6997801, Israel; 14 Department of Genetics and Developmental Biology, The Rappaport Faculty of Medicine and Research Institute, Technion – Israel Institute of Technology, Haifa 32000, Israel; 15 Department of Biomedical Engineering, Tel Aviv University, Tel Aviv 6997801, Israel; 16 The Center for Nanoscience and Nanotechnology, Tel Aviv University, Tel Aviv 6997801, Israel; 17 Sagol Center for Regenerative Medicine, Tel Aviv University, Tel Aviv 6997801, Israel

**Keywords:** brain targeting, lipid nanoparticles, neuroinflammation, Parkinson’s disease, central nervous system.

## Abstract

Monoclonal antibodies (mAbs) hold promise in treating Parkinson’s disease (PD), although poor delivery to the brain hinders their therapeutic application. In the current study, we demonstrate that brain-targeted liposomes (BTL) enhance the delivery of mAbs across the blood-brain-barrier (BBB) and into neurons, thereby allowing the intracellular and extracellular treatment of the PD brain. BTL were decorated with transferrin to improve brain targeting through overexpressed transferrin-receptors on the BBB during PD. BTL were loaded with SynO4, a mAb that inhibits alpha-synuclein (AS) aggregation, a pathological hallmark of PD. We show that 100-nm BTL cross human BBB models intact and were taken up by primary neurons. Within neurons, SynO4 was released from the nanoparticles and bound to its target, thereby reducing AS aggregation, and enhancing neuronal viability. *In-vivo*, intravenous BTL administration resulted in a 7-fold increase in mAbs in brain cells, decreasing AS aggregation and neuroinflammation. Treatment with BTL also improved behavioral motor function and learning ability in mice, with a favorable safety profile. Accordingly, targeted nanotechnologies offer a valuable platform for drug delivery to treat brain neurodegeneration.

## Introduction

Parkinson’s disease (PD) is known to impact approximately 1% of the population aged 60 and older, with limited treatment modalities. This neurodegenerative condition is primarily characterized by the degeneration of dopaminergic neurons in the midbrain substantia nigra (SN),^[1–2]^ resulting in debilitating motor symptoms such as tremors, poor gait, and impaired speech, which deteriorate with disease progression.^[2–3]^ PD is associated with neuronal, microglial, and astrocyte dysfunction, leading to a loss of normal homeostatic and/or acquisition of neurotoxic functions.^[4]^ To date, no single factor underlying PD has been identified, although genetic and environmental factors have been implicated in its etiology.^[5]^

A pathological hallmark of PD is the presence of inclusion bodies (i.e., Lewy bodies), composed primarily of aggregated alpha-synuclein (AS).^[6]^ AS mutations, such as A53T and A30P, were found to induce abnormal AS accumulation and, consequently, oligomerization and aggregation.^[7]^ In the brain, AS is detected in various conformations, from unfolded monomers to soluble oligomers and insoluble fibrils.^[7]^ AS oligomers could either aggregate to form toxic β-sheet fibrils, leading to the propagation of AS pathology, or form structures that do not propagate but are, nevertheless, toxic.^[8]^ Reducing AS oligomerization and aggregation has been suggested as a potential treatment for PD.^[9]^ Accordingly, monoclonal antibodies (mAbs), such as SynO4, were designed to bind epitopes in the non-amyloid β component and C-terminal regions of AS, thereby preventing further AS aggregation.^[10–12]^ However, SynO4 has poor brain penetration properties, predominantly exhibiting extracellular activity in the absence of a suitable carrier.^[13]^ These limitations can be overcome using nanoscale drug delivery systems, as these systems combine brain-targeting capabilities with the ability to deliver multiple mAbs within each nanoparticle intracellularly.^[14–15]^

The blood-brain barrier (BBB) remains the primary obstacle for drug targeting to the brain. This physical and metabolic obstacle comprises a monolayer of endothelial cells on the vascular lumen side, with pericytes and astrocytes on the basal side providing support and directly interacting with the endothelial cells. Tight junctions between endothelial cells restrict the passage of molecules into the brain, with access primarily regulated through receptor-mediated transcytosis.^[16–17]^

Although nanoparticles have been employed as drug delivery systems for treating cancer ^[18]^ and targeting drugs to specific organs, such as the lungs and liver^[19–21]^, their ability to penetrate the brain during PD remains unexplored. This can be achieved by adding targeting moieties to the outer surface of the nanoparticles to enhance their BBB penetration.^[14, 22–32]^

To increase BBB penetration in PD, one promising approach ^[33–35]^ is based on the overexpression of transferrin (TF) receptor (TfR1) on the BBB endothelium of patients with PD, allowing transcytosis across the barrier.^[36–37]^

Accordingly, we hypothesized that conjugating TF, a 76-KDa protein with affinity to the TfR1, to the liposome surface can enhance liposomal uptake in the brain (Figure 1A).

Hence, in the current study, we evaluated the ability of TF-conjugated liposomes loaded with SynO4 mAbs (hereafter, brain-targeted liposomes, or BTL) to cross the BBB and deliver their therapeutic cargo to brain parenchyma cells, including endothelial, neurons, and glial cells. We further assessed the efficacy of formulated BTL to reduce neuroinflammation and slow PD progression in mice. Our findings support the use of targeted nanoparticles as systems for the targeted delivery of biologics to treat PD.

## Results and Discussion

### Synthesizing BTL

To achieve BBB penetration, we engineered BTL by conjugating TF to the outer surface of 100-nm liposomes loaded with SynO4. SynO4-loaded BTL were prepared via two main synthetic steps. First, SynO4 mAbs were loaded into liposomes through a thin-film hydration process.^[38]^ Then, TF was conjugated to amine-functionalized polyethylene glycol (PEG) extending from the outer surface of the liposomes using EDC/NHS coupling chemistry^[39]^ (Figure 1B).

For the fabrication steps, the working temperature was selected to preserve the bioactivity of SynO4 during the process (T=45°C; see methods, Figure S1, and Figure S2). Considering the liposome composition, 1,2-dipalmitoyl- sn-glycerol-3-phosphocholine (DPPC; 16:0, Tm=41°C) was selected as the main lipid at a molar of 65%, along with cholesterol (30 mol%), 1,2-distearoyl-sn-glycero-3-phosphoethanolamine-N-[methoxy(polyethyleneglycol)-1000] (DSPE-PEG1000; 2.5 mol%), and 1,2-distearoyl-sn-glycero-3-phosphoethanolamine-N-[amino(polyethyleneglycol)-2000] (DSPE-PEG2000-NH2; 2.5 mol%). The average BTL size was 113.5±1.5 nm, with 33±6 antibodies loaded into each liposome and 95±23 TF-targeting molecules conjugated to their outer surface. (Figures 1C, D, and S3). The protein mass of loaded SynO4 mAbs was 0.142 mg mAb/94.5 mg liposome-lipid, comparable with the drug mass loaded into clinically approved liposomes. Under physiological conditions, the established BTL composition allowed mAb release over 48 h (*p*=0.0044) (Figure S4). BTL remained stable, and mAbs were biologically active for two weeks at 4°C and for one week at 25°C (Figure S6).

Additionally, untargeted, control liposomes loaded with SynO4 mAbs were prepared and had an average size of 114.3±0.3 nm, comprising 73±10 SynO4 units per liposome (Figure S3 and S5, respectively). Notably, conjugating TF to the liposome surface displaced the surface absorbed SynO4.

Importantly, targeting moieties were conjugated to the nanoparticle surface without impacting the docking region within the targeting moiety, ensuring that this region remained unaltered during the conjugation process. Therefore, we selected the amine PEG linker to conjugate carboxyl groups onto the external surface of the TF moiety (Movie S1). Based on protein dynamics analysis, each TF molecule presented ~38 COOH binding sites to allow conjugation of the PEG entity without compromising the biological binding site. The TF binding site (TfR1-TF) comprises six main amino acids (His249, Tyr95, Asp63, Asp221, Asp356, Tyr188) that form multiple chemical interactions with the TF molecule.^[40–42]^

To visualize the presence of TF on the liposome surface, gold nanoparticles (GNPs) were conjugated to TF and imaged using cryogenic transmission electron microscopy (cryo-TEM). The gold nanoparticles (5 nm in diameter, dark objects in Figure 1E) were in close proximity (<15 nm) to the outer surface of the liposomes (Figure S7), in agreement with their positioning on the distal end of PEG2000 extending from the liposome surface.

PEG molecules extending from the outer surface of nanoparticles can induce steric interference, preventing the binding of targeting moieties to their biological target.^[43]^ Only 3.8±0.9% of PEG chains extending from the BTL were conjugated to TF; the remaining PEG molecules extending from the liposome surface were capped with a non-binding methyl group on their distal side. These non-binding PEG molecules could improve the steric stability of liposomes, as well as extend their circulation time, however, the length of the non-binding PEG affected the BTL targeting capacity.^[44]^

To address PEG-induced steric hindrance, we examined the uptake of liposomes with TF conjugated to short (1000) or long (2000) PEG moieties using human brain endothelial cells (hCMEC/D3) (Figure 1F). Nanoparticles with TF molecules conjugated to longer PEG moieties exhibited greater endothelial uptake. Specifically, nanoparticles with TF conjugated to PEG2000 (PEG2000-TF) combined with unconjugated PEG1000 showed better endothelial uptake than particles with PEG2000-TF together with unconjugated PEG2000 (*p*<0.0001). Based on these findings, we postulate that neighboring PEG molecules exert steric hindrance when the length of an unconjugated PEG-lipid is similar to that of a targeting-ligand-displaying PEG lipid, inhibiting efficient interaction between the targeting ligand and its receptor. Accordingly, we utilized the PEG2000-TF/PEG1000 formulation for subsequent experiments. To optimize the number of TF moieties per nanoparticle, we screened liposomes with an increasing number of TFs on their surface (Figure S8). We observed that increasing the number of TFs conjugated to the liposome surface improved endothelial uptake of TF-conjugated liposomes without SynO4 mAbs (hereinafter, BTL (empty)) (*p*<0.0001). Maximal endothelial uptake was achieved at a TF concentration of 109±11 per liposome (achieved by adding 20 mg/ml TF during the formulation process), at which the liposome surface was saturated. Considering financial cost versus cell uptake benefits, liposomes with 95±23 TF units were deemed optimal BTL (achieved by adding only 10 mg/ml TF during the formulation process; see Methods and Figure S9).

Recent *in vitro* and *in-vivo* studies have suggested that PEG molecules on the surface of liposomes can enhance liposomal uptake by neurons.^[45–47]^ The length of the PEG chain extending from the liposome surface plays an important role in cellular uptake and targeting capacity. Our findings indicate that conjugating TF moieties to a long PEG2000 chain, maintaining PEG1000 as the remaining steric PEG molecules, results in superior uptake by brain endothelial cells. This result can be attributed to the steric effect of neighboring PEG molecules on the formulation, camouflaging the TF targeting moiety from its biological target when markedly long.

### BTL cross the BBB

We next assessed the ability of BTL to cross the BBB using an *in-vitro* BBB model of the neurovascular unit (NVU). We also examined the integrity of BTL after crossing the BBB. The *in-vitro* BBB model system comprised a Transwell\Chip plate containing a compartment of induced pluripotent stem cells (iPSC) derived human brain microvascular endothelial cells (BMECs) placed, in a noncontact manner, atop a basolateral compartment of primary cortical neurons and astrocytes (Figure 2A). The degree of BTL transport across the endothelial monolayers was determined by measuring the liposomal content in media on the basolateral side of the BBB model. BTL permeability across the monolayer increased gradually from the donor to the acceptor cell (Figure 2B) without affecting tight junction integrity in the endothelial monolayer (Figure S10). According to cryo-TEM analysis of media on the basolateral side of the barrier, BTL remained intact after crossing the BBB (Figure 2C). Furthermore, we conducted live imaging to monitor the passage of BTL across the BBB layers. We found that particles were internalized by BMECs and migrated to the basolateral side of the BBB (Figure S11 and Movie S2).

Subsequently, we used direct stochastic optical reconstruction microscopy (dSTORM) to determine the subcellular localization of BTL in capillary endothelial cells while crossing the BBB (Figure 2D).^[48]^ Healthy mice were intravenously administered AZDye 647-labeled BTL. Brains were fixed, and cortical tissue sections were immunolabeled with GLUT1 (glucose transport protein type 1) to identify the endothelium in capillary cross-sections and LAMP1 (lysosome-associated membrane protein 1) to detect lysosome organelles within cells. Tissue dSTORM imaging confirmed that BTL crossed the BBB. To further validate this finding, we examined a shorter exposure time point of 30 min to capture BTL within endothelial cells (Figure 2D(i)). Additionally, BTL signals were observed in the brain parenchyma 6 h post-injection (Figure 2D(ii)). Nanoscale localization of single molecules showed minimal BTL colocalization with LAMP1, i.e., the lysosomal marker. The fluorescent signal from the lysosome molecules (LAMP1) co-localized with only 1.7±1.3% of the BTL fluorescent signal, suggesting that BTL escape lysosomal degradation on entering the endothelium.

Next, we determined the spatial and temporal dynamics associated with BTL entry into brain neurons (Figure S12, 2E, and F). We used in-utero electroporation of pCAG-EGFP plasmid at embryonic day 14 (E14) to fluorescently label the excitatory layer 2/3 cortical cells within the mouse cerebral cortex.^[49]^ GFP-positive mice were identified at birth and were left to mature until postnatal day 30 (p30), at which point they underwent cranial window surgery.^[50]^ To track BTL entry into cortical neurons *in-vivo*, we used two-photon fluorescence lifetime imaging^[50]^. We then compared the fluorescence lifetime of neurites and cell bodies post-BTL injection. Two hours post-injection, we identified small punctate structures characterized by short lifetimes along dendritic branches; however, these structures were absent within cell bodies (Figure 2E and F; soma: 0.057Δns±0.03Δns [*p*=0.7293] vs. 0 h, and neurite: 0.306Δns±0.06Δns [*p*<0.0035] vs. 0 h). However, 5 h post-injection, we detected distinct punctate formations on both cell bodies and neurites, displaying reduced lifetime values (Figure 2E and F; soma: 0.175Δns±0.03Δns [*p*<0.0012] vs. 0 h, and neurite: 0.487Δns±0.05Δns [*p*<0.0001] vs. 0 h). Accordingly, in a healthy cortex, BTL particles were internalized by neuronal processes within 2 h and by cell bodies within 5 h post-injection.

Overall, these findings suggest that BTL crossed the BBB, evaded lysosomal degradation in endothelial cells, and were taken up by neurons and cells in the brain parenchyma. In addition, BTL were distributed to other organs, mainly the liver and the kidneys, similarly to other reported nanoparticles.^[51]^

### mAb delivery to neurons

We compared the neuronal uptake and activity of SynO4-loaded BTL with those of free SynO4. Confocal microscopy revealed the presence of BTL within the cell body and along the exons of primary murine cortical neurons overexpressing human AS-A53T, incubated overnight with BTL (Figure S13, 3A, and 3B). Within neurons, SynO4 mAbs were released from the liposome and engaged with target AS aggregates. The free SynO4 antibody control group exhibited a weak cellular signal, suggesting that free antibodies failed to enter neurons efficiently (*p*<0.0001) (Figure 3C).

AS-A53T-overexpressing differentiated human SH-SY5Y (i.e., PD-induced cells) incubated overnight with BTL or free SynO4 were subjected to flow cytometric analysis, demonstrating that 63.4±2.8% of BTL-treated cells were SynO4 positive, compared with 2.8±2.0% of free-SynO4-treated cells (*p*<0.0001).

Furthermore, we assessed the uptake and target engagement of BTL using differentiated human SH-SY5Y cells seeded with exogenous AS aggregates. Following overnight treatment, super-resolution microscopy revealed BTL accumulation in both neuronal cell bodies and fibers (Figure S14a and b). Twelve hours post-incubation, 26±8% of SynO4 mAb was released from BTL intracellularly, resulting in colocalization and target engagement with 73±7% of intracellular AS aggregates (Figure S14c and d). Accordingly, our findings indicate the effective intracellular delivery of mAbs using BTL. Within neurons, mAbs were released from the liposomes and bound to their target (Figure S15).

To determine the efficacy of BTL in reducing AS aggregation and improving neuronal viability, AS-A53T-overexpressing primary murine cortical neurons (PD-induced cells) were treated with either BTL or free SynO4. After staining for phosphorylated AS, the cells were visualized using dSTORM microscopy (Figures 3D and S16), and the number of AS aggregates was analyzed (Figure 3E). BTL-treated PD-induced cells exhibited reduced AS aggregation when compared with untreated PD-induced cells (*p*=0.0003) and PD-induced cells treated with free SynO4 mAbs (*p*<0.0001). Furthermore, treatment with BTL reversed the cell phenotype to a healthy basal level of AS expression and aggregation. Conversely, treatment with free SynO4 mAb did not reduce the number of AS aggregates.

To determine the impact on cell viability, we treated AS-A53T-overexpressing differentiated human SH-SY5Y (i.e., PD-induced cells) with BTL or free SynO4 (Figure 3F). BTL-treated cells exhibited a 76±2% reduction in the level of late apoptotic/necrotic cells when compared with untreated PD-induced cells (*p*<0.0001) (Figure 3G). Free SynO4-treated neurons showed a negligible reduction in cell death (*p*=0.0346), reflecting the low cellular uptake of mAbs. In addition, treatment with empty BTL (without SynO4) did not significantly reduce the number of apoptotic cells, indicating the therapeutic effect was primarily attributed to SynO4 mAbs loaded into BTL.

These *in-vitro* findings underscore the potential therapeutic efficacy of BTL to reduce AS aggregation and inhibit neuronal cell death.

### BTL accumulate in the brain of PD mice

Overexpression of TfR1 on the BBB endothelium is a pathological hallmark of PD.^[52–53]^ Given that the BTL were designed to target TfR1, we first validated TfR1 overexpression in the viral PD-induced mouse model. To overexpress AS, C57BC/6JRccHsd male mice were inoculated with an adeno-associated virus (AAV) encoding human AS (AAV2/6-hSyn1-Human SNCA-WPRE-polyA) administered into the right substantia nigra (Figure S17). The brains of AAV-inoculated PD mice showed a 3-fold higher relative TfR1 expression than the brains of healthy mice (*p*<0.0005) (Figure 4A).

Next, we intravenously injected Cy5-labeled BTL (empty) or Cy5-labeled untargeted liposomes and examined liposome levels in the PD brain (intracellular plus extracellular) and other organs 12 h post-injection (Figures 4B, C, and S18). The accumulation of intravenously administered BTL in PD brains was 3-fold higher than that of untargeted liposomes (*p*=0.1061), supporting the hypothesis that TF improves BBB penetration and brain targeting (Figure 4C). In addition, PD brains exhibited ~2-fold higher BTL accumulation than healthy brain tissue (*p*=0.08533).

In a complementary study, we assessed whether treatment with BTL could increase mAb levels in the brain. PD mice were intravenously administered BTL or free SynO4 mAbs; brains were subsequently harvested and processed for antibody quantification (both intracellular and extracellular) using a direct ELISA assay. Brain tissues of BTL-treated mice showed 2.3-fold higher levels of mAbs than those of free SynO4-treated mice (*p*<0.05) (Figure 4D). Using confocal microscopy, we examined frozen brain sections to assess intracellular liposome accumulation in the brain following intravenous administration of Cy5-labeled BTL loaded with Cy3-labeled SynO4 or free Cy3-labeled SynO4 (Figure S19). Our findings confirmed that SynO4 mAb encapsulation in targeted liposomes enhances brain delivery to both the brain parenchyma and brain cells.

To assess the magnitude of nanoparticle delivery compared with that of free antibody delivery at the cellular level, PD mice were intravenously administered Cy5-labeled components: BTL (empty), free SynO4, or TF-targeted-SynO4 (i.e., SynO4 with TF conjugated directly to the mAb). Following intravenous administration, brain tissues were harvested and digested for single-cell analysis, and Cy5-positive cells were recorded (Figure 4E). A quantitative FACS analysis 12 h post-injection demonstrated that the total number of Cy5-positive cells in the PD brains was 5.4-fold higher than that in healthy mouse brains (*p*<0.005) (Figure 4F). Following BTL delivery, the number of Cy5-positive cells was 7.6-fold greater than that after free antibody delivery (*p*<0.005) and 1.6-fold greater than that after targeted free SynO4 delivery (*p*<0.00181).

To evaluate BTL distribution in different cell populations in PD and healthy brains, brain cells were labeled using an antibody panel for microglia (CD45+, CD11b+), endothelial cells (CD31+),astrocytes (CD44+), and neurons (CD24+; Figure S20 and S21). In PD brains, 35±11% of endothelial cells exhibited BTL positivity (Figure 4G(i)), with only 8±2% of endothelial cells exhibiting BTL-positivity in healthy brains (Figure 4G(ii)). Furthermore, 12±1% of neurons in PD brains were BTL positive (Figure 4G(i)) compared with 8±2% of neurons in healthy brains^[54]^ (Figure 4G(ii)). In both PD and healthy mice, microglia and astrocytes showed poor liposomal uptake (Figure 4G). The predominant accumulation of nanoparticles in brain endothelial cells can be explained by the fact that endothelial cells are the first point of contact for circulating BTL as they cross the BBB.^[16–17]^ Furthermore, endothelial cells overexpress TfR1 in the brain microvasculature, thereby enhancing the uptake of BTL via receptor-mediated transcytosis.^[36]^ PD is characterized by the death of dopaminergic neurons in the brain.^[3, 55]^ Accordingly, we next measured BTL uptake by dopaminergic neurons and compared it with that by other neuronal subtypes. After intravenous administration of BTL, the brains of PD mice were harvested, and brain cells were labeled with specific markers for microglia, endothelial cells, astrocytes (as detailed above), as well as dopaminergic neurons (Dopamine Transporter+) (Figure S22a). The mean fluorescent intensity (MFI) of Cy5-labeled BTL within dopaminergic cells was 6222±2154, i.e., ~1.9-fold higher than the MFI of 3200±1514 observed in other neuronal subtypes or oligodendrocytes. Similar results were also observed for the SynO4 content in dopaminergic neurons. Intravenous BTL administration increased the MFI of Cy3-labeled SynO4 mAbs (17,840±9737, ~2.3-fold) within dopaminergic neurons when compared with the intensity of 7835±5669 detected in other neuronal subtypes or oligodendrocytes (Figure S22b and c). The higher BTL uptake in dopaminergic neurons could be attributed to the dopaminergic overexpression of TfR1 during PD.^[54, 56]^

To assess BTL internalization within dopaminergic neurons, we used confocal microscopy to visualize patient-derived dopaminergic neurons with amplified copies of the SNCA gene.^[57]^ After incubation, BTL was detected within the cytoplasm and along the exons of patient-derived PD neurons (Figure 4H).

### BTL reduce AS aggregation and neuroinflammation in AAV-inoculated PD mice

To examine the ability of BTL to reduce AS aggregation in the brain, AAV-inoculated mice overexpressing AS (PD-induced) were treated with free SynO4, BTL, or left untreated (disease control), and a healthy group was used as a second control. Each group was injected intravenously every other day for two or four weeks (Figure 5A). Immunohistochemistry was performed to assess AS levels, neuronal survival, and levels of activated microglia and reactive astrocytes in the substantia nigra. AS aggregates were labeled using a 5G4 antibody, whereas the number of dopaminergic neurons was detected using a tyrosine hydroxylase (TH) antibody, which recognizes TH enzyme expression by dopaminergic neurons (Figures 5B(i), C(i), S23, and S24). To examine neuroinflammation^[58]^, activated microglial cells were detected using the ionized calcium-binding adaptor molecule 1 (Iba1) antibody (Figure S25). Furthermore, the number of reactive astrocytes was calculated using the glial fibrillary acidic protein (GFAP) antibody, expressed exclusively in astrocytes (Figure S26).

Two weeks after treatment initiation, the BTL-treated group showed a significant reduction in the percentage of extracellular AS aggregates (76±12% reduction) when compared with the free antibody antibody-treated group (45±10% reduction; *p*=0.0004) (Figure 5B(ii)). Analyzing the number of cells exhibiting aggregated AS and the total amount of aggregated AS (extracellular and intracellular), BTL-treated mice exhibited lower levels of AS-positive cells (~2.8-fold, *p*=0.0006) and total AS accumulation (~2.5-fold, *p*=0.0005) than untreated PD mice (Figure S27a and b).

We next evaluated the percentage of live dopaminergic neurons by comparing the number of neurons on the inoculated side (right hemisphere) with those on the non-infected side (left hemisphere). In the untreated group, the two-week viral PD injection reduced dopaminergic neurons by 24±12% (*p*=0.0026) (Figure 5B(iii)). The BTL-treated group showed a slight improvement in dopaminergic neuron survival (81±9% survival vs. 76±12%-healthy: *p*=0.0147) when compared with the free SynO4-treated group (73±12% survival vs. 76±12%-healthy: *p*=0.0002).

Given that the reactive glia process involves molecular and morphological changes in astrocytes and microglial cells, including increased expression of GFAP^[59–62]^ and Iba1^[63–64]^ markers, respectively, we assessed these biomarkers in mice two weeks post-viral injection-induced PD. Activated glial cells were reduced in both BTL- and free antibody-treated groups when compared with those in the untreated PD group (*p*<0.0001) (Figure 5D(i) and D(ii)). However, BTL treatment induced a more significant reduction in activated microglia cells (~3.4-fold, *p*<0.0001) than free SynO4 treatment (~1.7-fold, *p*=0.0032) (Figure 5D(i)).

Four weeks post-treatment, BTL-treated mice showed a further reduction in the percentage of extracellular AS aggregates (69±9% reduction) when compared with the 50±10% reduction observed in free SynO4 mAb-treated mice (*p*=0.0198) (Figure 5C(ii)). Compared with AS-positive cell numbers in the untreated PD group, the number of AS-positive cells in the BTL-treated group decreased by ~1.5-fold (*p*=0.0097) (Figure S27c), and the total amount of AS aggregates reduced by ~3-fold (*p*<0.0001) (Figure S27d).

Overall, BTL treatments reduce AS aggregation when compared with treatment with free SynO4 and other controls.

Four weeks after viral AS inoculation, the untreated PD group exhibited dopaminergic neuron survival of 60±11% (Figure 5C(iii)). Treatment with BTL delayed the loss of dopaminergic neurons, resulting in a neuronal survival rate of 70±10%, whereas treatment with free SynO4 mAbs achieved survival of only 53±26%, thereby demonstrating a trend of dopaminergic neuron survival. Four weeks post-viral injection-induced PD, we observed that glial inflammation continued progressing (Figure 5E(i) and E(ii)). Both BTL- and free SynO4 mAb-treated PD mice showed a significant reduction in the number of activated microglia cells (*p*<0.0001) (Figure 5E(i)). The BTL-treated group exhibited a marked reduction in the number of inflammatory astrocytes when compared with the untreated PD group (*p*=0.0363); treatment with free SynO4 mAbs failed to reduce the number of activated astrocyte cells (*p*=0.9375) (Figure 5E(ii)).

Taken together, our findings indicate that SynO4 mAb-loaded BTL reduce AS aggregation and neuroinflammation, thereby decelerating neuronal degeneration. Based on the findings of examined parameters, intravenous administration of the liposomal formulation afforded greater efficacy than intravenous free antibody administration, highlighting the ability of the developed brain-targeted delivery system to overcome challenges encountered by brain biological therapies, including BBB permeation, cell membrane penetration, and neuroprotection.

### BTL improve motor function and motor learning in PD mice

Next, we examined the effect of BTL treatment on motor function, as well as on motor learning (Figures 6 and S28). AAV-inoculated mice overexpressing AS were randomly divided into four groups: untreated PD mice, PD mice treated with free SynO4 mAbs, PD mice treated with BTL, and healthy mice (control group). Treatment groups received intravenous injections every other day for four weeks and were examined using the rotarod apparatus at two and four weeks post-treatment (Figure 6A). Two weeks post-treatment, the BTL group achieved motor function outcomes similar to the healthy control group (*p*<0.7607) (Figure 6B). Conversely, free SynO4 mAb-treated mice showed a reduced latency to fall when compared with BTL-treated mice (*p*<0.0074). Additionally, we evaluated the short-term motor learning capacity of mice (Figure 6C). Both healthy and BTL-treated mice showed gradual improvement in performance, with increasing latency to fall (Healthy and BTL day 1 vs. Day 3: *p*<0.0001). Free SynO4-treated and untreated PD groups showed reduced motor learning, maintaining a stable performance capacity (PD Day 1 vs. Day 3: *p*<0.0497; free SynO4 Day 1 vs. Day 3: *p*<0.0138). On day 3 of measurement, we noted a significant difference between the BTL group and the untreated PD (*p*<0.0067) and free SynO4-treated (*p*<0.0025) groups.

Four weeks post-treatment, the mice underwent the rotarod test to determine the long-term motor learning capacity (Figure 6D and E). Healthy and BTL-treated groups showed a 2-fold improvement in performance on day 3 of measurement (the end point of motor learning) when compared with that observed at experiment initiation (Figure 6D). Untreated PD and free SynO4-treated mice had a shorter latency to fall than healthy control mice (*p*<0.0042 and *p*<0.0106, respectively); the difference was not significant in BTL-treated mice when compared with healthy mice (*p*=0.4636). Free SynO4-treated mice showed values comparable with untreated PD mice (*p*=0.9906), whereas treatment with BTL improved performance (*p*=0.2047). The untreated PD group showed no significant improvement from the first day of motor functioning to the last day of motor learning (Figure 6E).

This behavioral study demonstrates the potential of BTL in improving motor function and learning abilities in virally inoculated PD mice (Movie S3).

### Preliminary safety of BTL in mice

To evaluate the safety profile of BTL, mouse blood samples were collected after 40 days of treatment. Representative histopathological organ sections from the liver (Figure 6F(i)), kidney (Figure 6G(i)), and spleen (Figure 6H(i)) were analyzed. Hematoxylin and eosin-stained tissue sections were used to examine cell structure. No apparent differences were detected between healthy and BTL-treated groups in any examined organs. Specifically, no steatosis and no peri-portal or parenchymal inflammation were observed in the liver. The glomerular and tubular structures were normal, with no interstitial inflammation.

To evaluate potential hepatotoxicity (Figure 6F(ii)), we measured levels of hepatic enzymes, including aspartate transaminase (AST) (Figure S29a), alanine transaminase (ALT) (Figure S29b), lactate dehydrogenase (LDH) (Figure S29c), alkaline phosphatase (ALP) (Figure S29d), and total bilirubin (T.Bil) (Figure S29e). No significant differences were detected between the healthy and BTL-treated groups. Treatment with free SynO4 induced a slight increase in hepatic enzyme and bilirubin levels (Figures 6F(ii), S29a, and e).^[65]^ Nephrotoxicity (Figure G(ii)) was assessed by measuring blood levels of creatinine (Figure S29f), urea (Figure S29g), and albumin (Figure S29h). No appreciable differences were detected between healthy and BTL-treated groups. Hematologic parameters (Figure 6H(ii)) were determined by measuring %lymphocytes (Figure S29i), %neutrophils (Figure S29j), lymphocyte antibodies (Figure S29k), and neutrophile antibodies (Figure S29l). PD, free SynO4, and BTL-treated groups showed elevated levels of white blood cells (WBC) (Figure 6H(ii)) and lymphocytic antibodies (Figure S29k), possibly due to elevated levels of AS, capable of inducing inflammation and an immune response.^[66]^ However, treatment with free SynO4 reduced neutrophil levels (neutropenia, Figure S29j).

Taken together, these data suggest that BTL administration over a 4-week period was favorably tolerated and did not cause organ toxicity.

## Conclusions

Herein, we engineered TF-targeted liposomes loaded with SynO4 (BTL) to treat PD. The synthetic approach involved loading SynO4 mAbs into 100-nm liposomes and then conjugating a targeting moiety (TF) to their outer surface via covalent bonds. It is well established that SynO4 can bind and inhibit AS aggregation; however, its free, non-liposomal form has poor brain penetration capacity.

To preserve the bioactivity and integrity of SynO4 during the formulation process, DPPC, a phospholipid with a favorable phase transition temperature (Tm=41°C), was selected as the main lipid component of nanoparticles. BTL with 95±23 TF units on their outer surface, encapsulating 33±6 SynO4, remained stable for two weeks at 4°C.

*In vitro*, BTL crossed BMEC endothelial BBB monolayers, escaping endocytic pathways and retaining their structural and functional integrity.

BTL were readily taken up by patient-derived neurons and primary cortical neurons, distributing throughout the cell body and axons. Within neurons, SynO4 was released, binding its AS target, resulting in reduced AS aggregation and enhanced neuronal viability. However, free SynO4 did not penetrate neurons efficiently.

*In-vivo,* intravenously administered BTL was favorably accumulated in the brain of PD mice, compared with untargeted liposomes, or TF-SynO4.

Most importantly, treatment with BTL slowed the progression of PD in model mice. Treatment with BTL reduced AS aggregation and cell death in PD neurons when compared with free SynO4 treatment. Two and four weeks of intravenous BTL administration significantly reduced intracellular and extracellular AS aggregation and neuroinflammation in PD mice. Furthermore, treatment with BTL improved motor function and learning capabilities while maintaining a favorable safety profile.

Collectively, the findings of the current study demonstrate that targeted nanoparticles cross the BBB and deliver antibodies intracellularly and extracellularly to the brain during PD, supporting the utility of this approach for treating other neurodegenerative diseases.

## Experimental Section

### ELISA establishment

An in-house ELISA assay using the direct ELISA method was developed for the Anti-Human SNCA Therapeutic (SynO4) Antibody (TAB-0750CLV-L; Creative Biolabs, USA) (Figure S2). A 384-well plate was coated with 1.4 μg/ml Recombinant Human Alpha-Synuclein protein aggregate (Active) (ab218819; Abcam, UK), sealed, and incubated overnight at 4°C. The next day, plates were blocked in 5% fetal bovine serum (FBS) (K210430; Rhenium, Israel) in PBST (Dulbecco’s phosphate-buffered saline [PBS]) (D8537; Sigma-Aldrich, Israel) with 0.05% Tween20 (P1379; Sigma-Aldrich); this was followed by the addition of known concentrations of SynO4 antibody. Next, plates were washed to remove PBST, followed by incubation with the secondary antibody (1:100,00; Goat Polyclonal to Mouse IgG1-HRP) (ab6789; Abcam, UK). TMB ELISA Substrate (ab171522; Abcam) was used for signal development, with kinetics absorbance measured using a plate reader (reading at 650 nm with 2 min intervals for 1 h). A linear calibration curve for the SynO4 antibody (Figure S1b) was generated based on the described ELISA setup. For generating the standard IC_50_ curve (Figure S1c), SynO4 was pre-incubated with a 10-fold serial dilution of AS aggregates.

To determine the working temperature for the nanoparticle preparation with the SynO4 antibody, antibody samples were pre-heated at different temperatures (35, 45, 55, and 65°C) for 1 h, and a non-heated antibody sample (25°C) was also used. The samples were either maintained overnight at 4°C or immediately incubated with known concentrations of AS aggregates to generate IC_50_ curves (Figure S1f and S1d, respectively) and graphs of the maximum antibody activity percentage (Figure S1g and S1e, respectively).

### BTL fabrication

The thin-film method was used for liposome fabrication. First, 100 mM of total lipid mixture of DPPC (556610; Lipoid, Germany), cholesterol (C8667; Sigma-Aldrich), 1,2-distearoyl-sn-glycero-3-phosphoethanolamine-N-[methoxy(polyethylene glycol)-1000] (ammonium salt) (DSPE-PEG1000) (001096; Biopharma PEG, USA) and 1,2-distearoyl-sn-glycero-3-phosphoethanolamine-N-[amino(polyethylene glycol)-2000] (ammonium salt) (DSPE-PEG2000-NH2) (10455; Biopharma PEG), in molar percentages of 65:30:2.5:2.5, was dissolved in chloroform. To prepare untargeted liposomes, 1,2-distearoyl-sn-glycero-3-phosphoethanolamine-N-[methoxy(polyethylene glycol)-2000] (ammonium salt) (DSPE-PEG2000) (29232000; Lipoid) was used instead of DSPE-PEG2000-NH2.

Using a rotary evaporator, chloroform was evaporated (45°C, 50 [rpm] to obtain a thin homogenous lipid film, which was subsequently stored in a vacuum at -20°C for further use. Next, the film was hydrated with 2 mg/ml of SynO4 antibody solution in PBS for 1 h at 45°C and subjected to 50 rpm to obtain liposomes loaded with SynO4 mAbs; to prepare empty liposomes, the solution contained only PBS. Next, the liposome mixture was extruded through 400-, 200-, 100-, and 80-nm pore-size polycarbonate membranes (10417106, 110606, 110605, and 110604, respectively; LIFEGENE, Israel) using a LIPEX extruder (Northern Lipids, Canada), at 45°C, with a maximum working pressure of 15 bar, to obtain homogenous 100-nm liposomes. Finally, the non-encapsulated SynO4 antibodies were removed by dialysis against PBS (pH 7.4; 1:1000 volume ratio) using a 1000 kDa dialysis membrane (131486; Repligen, USA) at 4°C for 48 h. The buffer was exchanged four times during the 48 h, and the dialysis bag was replaced with a new bag at each change (Figure S2).

To quantify the encapsulated concentration of SynO4 mAbs within liposomes, liposomes were diluted several times and incubated in a blocking solution with 0.5% Triton-X-100 (93443; Sigma-Aldrich) for 1 h at 25°C to release SynO4 mAbs into the supernatant; for positive controls, untargeted liposomes or TF-conjugated liposomes without SynO4 mAbs (BTL (empty)) were mixed with a known concentration of SynO4. Next, the ELISA steps were performed as described above. To quantify the SynO4 concentration within liposomes, a linear calibration curve for the SynO4 antibody was obtained using the absorbance values. The SynO4 mAb encapsulated units per liposome were calculated using the calculated SynO4 concentration and the liposome particle concentration as follows:

Equation 1 $$C_{mAb}=X[\text{ug}/\text{ml}]xY[\text{ml}]=Z[\text{ug}]$$

Equation 2 $$N_{mAb}=C_{mAb}/Mw_{mAb}=Z[\text{ug}]/150,000[\text{gr}/\text{mole}]=Q\;\text{[mole}]$$

Equation 3 $${\text{Units}}_{mAb}=n_{mAb}xN=Q\;\text{[mole]}\;x6.022^{\wedge }10^{23}=mAb\text{units}$$

Equation 4 $${\text{Units}}_{\text{liposomes}\;}=L[\text{particles}/\text{ml}]\times Y[\text{ml}]=\text{Liposomes units}$$

Equation 5 $$\text{mAb encapsulated units per liposome}\;=\;{\text{Units}}_{mAb}/{\text{Units}}_{\text{liposomes}}$$

### TF-to-liposome conjugation

Human holo-Transferrin (T4132; Sigma- Aldrich) was cross-linked to the surface of liposomes using N-(3-dimethylaminopropyl)-N’-ethyl carbodiimide hydrochloride (EDC) (03450; Sigma-Aldrich) and N-hydroxysulfosuccinimide sodium salt (Sulfo-NHS) (FH24507; Tzamal D-Chem Laboratories Ltd, Israel). First, 10 mg/ml of TF (PBS), 15 mg/ml of Sulfo-NHS (PBS), and 15 mg/ml of EDC (DMSO) solutions were prepared. Second, each reagent solution was added to the TF solution to reach a 1:25 molar proportion (TF:Sulfo-NHS) and 1:10 molar proportion (TF:EDC). Using 1.2 M HCL, the pH of the TF solution with reagents was adjusted to 6. Third, the solution was mixed (450 rpm) at 25°C for 1 h. Fourth, the TF solution was dialyzed against PBS (pH 6; 1:1000 volume ratio) using a 12-14 kDa dialysis membrane (132700; Repligen) at 4°C overnight to remove excess reagents. Fifth, BTL (empty) were synthesized by mixing 100 nm empty NH2-liposomes (100 mM) with the TF solution; TF-conjugated liposomes loaded with SynO4 mAbs (BTL nanoparticles) were synthesized by mixing 100 nm NH2-liposomes loaded with SynO4 (100 mM) with the TF solution, to reach a final liposomal concentration of 50 mM and a molar proportion of 10:1 (liposomes:TF). Sixth, the pH of the liposome reaction was adjusted to 8.4 with 1 M sodium bicarbonate, and the reaction was incubated for 2 h at 450 rpm and maintained at 25°C. Lastly, non-conjugated TF was removed by dialysis against PBS (pH 7.4; 1:1000 volume ratio) using a 1000 kDa dialysis at 4°C for 48 h. The buffer was exchanged thrice (after 1 h, 4 h, and overnight, respectively) for BTL (empty); for BTL with SynO4, an additional exchange was performed the following day, followed by changing to a new bag overnight (Figure S2).

The Micro BCA Protein Assay Kit was used to quantify the number of TF units on the surface of liposomes (23235; Thermo Fisher, Rhenium, Israel). First, a TF calibration curve was prepared. To quantify the number of TF units on BTL with SynO4/empty, each tube of the calibration curve contained TF at a known concentration, with SynO4/empty liposomes (5%, 100 mM), and Triton-X100 (2%); the remaining volume was made up with PBS. In addition, the liposome sample was prepared by mixing 10% BTL with SynO4/empty (50 mM) and 2% Triton-X100 in PBS. Afterward, the samples (calibration curve and liposome) were incubated at 58°C for 1 h and centrifuged (13,000 rpm, 10 min) to induce bursting of liposomes and obtain a clean supernatant. The clean supernatant from each sample was transferred to a 96-well plate. The BCA reagent, prepared according to the manufacturer’s instructions, was added to wells comprising samples. The plate was sealed and incubated at 37°C for 1 h. Subsequently, the plate was cooled to 25°C (~8 min), and absorbance was measured at 562 nm using a plate reader (Tecan, Switzerland). A linear calibration curve for TF was obtained using the absorbance values to quantify the amount of TF (μg/ml) on the liposomes. Then, the number of TF units per liposome surface was calculated using the TF concentration and the liposome particle concentration as follows:

Equation 1 $$C_{TF}=X[\text{ug}/\text{ml}]xY[\text{ml}]=Z[\text{ug}]$$

Equation 2 $$n_{TF}=C_{TF}/Mw_{TF}=Z[\text{ug}]/76,000[\text{gr}/\text{mole}]=Q[\text{mole}]$$

Equation 3 $${\text{Units}}_{TF}=n_{TF}xN=Q\;\text{[mole]}\;x6.022^{\wedge }10^{23}=TF\text{units}$$

Equation 4 $${\text{Units}}_{\text{liposomes}}\;=\text{L [particles}/\text{ml}]xY[\text{ml}]=\text{Liposomes units}$$

Equation 5 $$\text{TF units per liposome surface}={\text{Units}}_{TF}/{\text{Units}}_{\text{liposomes}}$$

### Physical liposome characterization

The physical characteristics of liposomes, including mean size diameter (nm), particle size distribution, poly-dispersity index, and zeta potential (mV), were measured using dynamic light scattering with a Zetasizer Nano (ZSP, Malvern, UK). The particle concentration (particles/ml) was measured using the Zetasizer Ultra (Malvern). The drug-loading rate was calculated as follows (Figure S3):

Equation 1 $$C_{SynO4}[\mu \text{mole}]=\left(C_{SynO4}[\text{ug}/\text{ml, Figure}1\text{C and S}4]x1.5[\text{ml, sample volume}]\right)/150,000[\text{gr}/\text{mole}]$$

Equation 2 $$(\mu \text{mole antibody/}\mu {\text{mole lipid) min}}^{-1}=(C_{\text{Syn}O4}[\mu \text{mole}]/350[\mu \text{mole, total amount of}\text{lipids in the formulation])}60[{\text{min}}^{-1}\text{, hydration time]}$$

### BTL release profile

Using a 1000 kDa dialysis membrane, BTL, and free SynO4 mAbs were dialyzed against PBS (pH 7.4; 1:1000 volume ratio) at 37°C and 60 rpm. For each time interval, the remaining sample volume (from the dialysis bag) was weighed, and 6 μl of the sample was taken and stored separately at 4°C. Then, the sample solution was reinjected into the bag. Finally, 6-μl samples were diluted (1/1000) and measured using ELISA. The percentage of SynO4 released was determined as follows:

Equation 1 $$m_{SynO4(x\text{hour})}=C_{Syn04}[\text{mg}/\text{ml},\text{ELISA]}x\text{remaining sample volume in each time point}[\text{ml}]$$

Equation 2 $${\%}_{\text{SynO4 remaining in dialysis bag}}\;=m_{\text{SynO4}(x\text{hour})}/m_{\text{SynO4}(0\text{hour})}x100$$

Equation 3 $${\%}_{\text{SynO4 released in the external buffer}}\;=100-{\%}_{\text{SynO4 remaining in the dialysis bag}}$$

### Stability characterization of BTL

The size and particle concentration of BTL samples stored at 4 and 25°C were measured at six-day intervals over 31 days using a Zetasizer Ultra instrument. The activity percentage of encapsulated SynO4 mAbs was assessed every seven days, resulting in a total of five measurements, using the developed ELISA.

### Percentage of TF molecules on the BTL surface

The nanoparticle surface area occupied by TF molecules was quantified as follows:

Equation 1 $${\text{Units}}_{TF}x\pi r^{2}\text{(surface area of a head cylinder)}=95\pm 23x\pi (2.4\text{nm}{)}^{2}=1716.9\pm 422\text{nm}$$

Equation 2 $$4\pi r^{2}(\text{surface area of a sphere (liposome)})=4\pi (59.7\text{nm}{)}^{2}=44787.7\text{nm}$$

Equation 3 $$\%TF\text{molecules}=\text{Units}TFx\pi r^{2}\text{(surface area of a head cylinder)}/4\pi r^{2}\text{(surface area of a sphere (liposome))}\times 100\%=3.8\%\pm 0.9\%$$

### Conjugation of GNPs to TF-liposomes

GNPs were attached to TF-liposomes using a 5 nm NHS-Activated Gold Nanoparticle Conjugation Kit (Cytodiagnostics Inc, Canada, CGN5K-5-2). First, 10^13^ particles/ml TF-liposomes or untargeted liposomes (negative control) were mixed with 5 nm NHS-GNPs (10^13^ particles/ml) and then incubated at 25°C for 2 h. The reaction was then stopped by adding a quencher solution, according to the manufacturer’s instructions. Next, GNPs were bound covalently to TF-liposomes through amine groups of TF molecules. Finally, the GNP-TF-liposomes and GNP with the unconjugated untargeted liposome samples were imaged using cryo-TEM. Semiquantitative data of the minimal distance (nm) between GNPs and the lipid membrane was calculated using a code written in Fiji imaging software analysis^[67]^ (Figure S7).

### Cryo-TEM

Cryo-TEM imaging was performed using an FEI (Thermo Fisher Scientific) Talos 200C high-resolution TEM (Center for Electron Microscopy of Soft Matter, Wolfson Department of Chemical Engineering, Technion). The specimens were prepared at 25°C and 100% relative humidity under a controlled environment vitrification system. Drops of diluted liposomes were placed in a carbon-coated perforated polymer film, mounted on a 200-mesh TEM grid, and manipulated using tweezers. Then, 10^10^ particles/ml GNP-TF-liposomes or GNP with unconjugated untargeted liposomes were used for the imaging measurement; for the BBB *in-vitro* crossing evaluation, imaging was performed on a 24-h medium sample with TF-liposomes without a dilution step. After thinning with a filter paper-covered metal strip, the drop was immersed in liquid ethane at its freezing point (-183°C). Under controlled conditions, the grid was transferred into a Gatan 626 (Gatan, Pleasanton, CA) cryo-holder and imaged at -175°C. Digital images were captured using a highly sensitive FEI Falcon III direct-imaging camera. A volta phase plate was used to enhance image contrast.

### Dye-labeled liposomes synthesis

Cy5-labeled liposomes, Cy3-labeled liposomes, or AZDye 647-labeled liposomes were prepared using the thin-film method, as described above. Briefly, 1,2-distearoyl-sn-glycero-3-phosphoethanolamine (DSPE) (Lipoid, Germany, 565400) was mixed with Cy5 se(mono so3) (Cy5) (BD759435; BLD pharm, China), Cyanine3 NHS ester (Cy3) (ab146452; Abcam), or AZDye 647 NHS ester (1344-5; Click Chemistry Tools, USA) in a molar proportion 1:1 (DSPE:Dye) to yield Cy5-DSPE synthesized lipids, Cy3-DSPE synthesized lipids, and AZDye 647-DSPE synthesized lipids, respectively. Then, the labeled lipid was added to the lipid mixture at a 0.4% molar ratio, achieving labeled liposome compositions of DPPC:Cholesterol: DSPE-PEG2000-NH2: DSPE-PEG1000: DSPE-Dye in molar percentages of 64.6:30:2.5:2.5:0.4, respectively.

To maintain a constant number of TF units per liposome surface, Dye-DSPE was added to all liposome preparations.

### Dye-labeled SynO4 antibody synthesis

The dye-labeled SynO4 antibody was prepared using the EDC/NHS coupling reaction. Briefly, SynO4 antibody (2 mg/ml) was mixed with Cyanine3 NHS ester or Cy5 se(mono so3) at a molar proportion of 1:10 for 2 h, at a pH of 8.4, at 25°C. Then, the reaction solution was dialyzed against PBS (pH 7.4; 1:1000 volume ratio) using a 12–14 kDa dialysis membrane; the external buffer was exchanged three times (after 1 h, 4 h, and overnight, respectively). Finally, the absorbance of the Cy3-labeled SynO4 antibody or Cy5-labeled SynO4 antibody was measured at 280 nm using a plate reader. Finally, the number of dye molecules per antibody was calculated using the following equation:

Equation 1 $$\text{Dye/mAb}=A_{\text{Dye}}x\varepsilon_{mAb}/\left(A_{mAb}-A_{Dye}xCF\right)x\varepsilon_{\text{Dye}}$$

In all the dye-antibody conjugations, the ratio of dye/mAb ranged between 2 and 4.

### Cell culture

Each cell line or primary culture was cultured at 37°C in a humidified atmosphere containing 5% CO_2_, and a fresh medium was added every 2–3 days.

hCMEC/D3 immortalized human brain capillary endothelial cells (Merck, USA) were provided by A. Sosnik (Laboratory of Nanomaterials Science, Department of Materials Science and Engineering, Technion). Cells (adherent) were cultured in EndoGRO-MV Complete Media Kit (SCME004; Merck Millipore, USA), supplemented with 1 ng/ml FGF-2 (GF003; Merck Millipore). Cell plating was performed on a flask coated with collagen type I, rat tail (08115; Merck Millipore) solution in PBS (BSS-1005-B; Merck Millipore) at a dilution of 1:20 and then incubated for 1 h at 37°C. Then, trypsin-EDTA (SM2003C; Merck Millipore) was used for cell dissociation.

SH-SY5Y (ATCC), a thrice-cloned subline of the neuroblastoma cell line SK-N-SH, was provided by Prof. A. Fishman (Laboratory of Molecular and Applied Biocatalysis, Faculty of Biotechnology and Food Engineering, Technion). Cells (adherent) were cultured in a complete media comprising a 1:1 mixture of Dulbecco’s Modified Eagle’s Medium (DMEM) (D5796; Sigma-Aldrich) and Nutrient Mixture F12 HAM with sodium B (N4888; Sigma-Aldrich), supplemented with 1% (v/v) penicillin (10,000 units/ml), streptomycin (10 mg/ml; Pen-Strep) (030311B; Biological Industries, Israel), 1% (v/v) amphotericin B (Amp-B; 2.5 mg/ml) (030281B; Biological Industries), 10% (v/v) FBS, and 1% (v/v) non-essential amino acids (013401B; Biological Industries). In general, cells were dissociated and harvested using a cell scraper.

For neuronal differentiation, SH-SY5Y cells were seeded on 1% gelatin from porcine skin, gel strength 300, Type a (G2500; Sigma-Aldrich) coated plates, followed by incubation in complete media supplemented with 10 μM all-trans retinoic acid (RA) (R2625; Sigma-Aldrih) for 4 days. Then, the medium was replaced with a starvation media (complete media without FBS), supplemented with 50 ng/ml human BDNF factor (4500210; PeproTech, Israel) for an additional 4 days; the cells were fully differentiated after 7 days.

Cortical neuronal cultures of P1 and P2 mice pups (ICR strain) were established and provided by the U. Ashery Lab (School of Neurobiology, Biochemistry, and Biophysics, George S. Wise Faculty of Life Sciences, Sagol School of Neuroscience, Tel Aviv University), generated as previously described.^[68]^ Cells were plated into a 6-well plate (500,000 cells/ml) on a glass coated with a MatriGel (354230; Corning, USA) in Hanks balanced salt solution (HBSS) (H1387; Sigma-Aldrich) and 20 mm HEPES buffer (030251B; Biological Industries, Israel). Neurons were grown in Neurobasal A (NBA) (10888022; Gibco, Ireland), supplemented with B-27 (17504044; Gibco), GlutMax (35050061; Gibco), Pen-Strep, and 5% fetal calf serum (FCS) (10270106; Gibco) to support glial growth on the day of culture preparation. From the following day, the medium was exchanged twice weekly with a growth medium, which contained no serum and was similar to the plating medium, to avoid glial cell proliferation.

Dopaminergic neurons were derived from iPSCs of a human patient with PD with 4 copies of an SNCA gene mutation (AS gene), as previously described^[57]^, and cultured in the S. Stern lab (Sagol Department of Neurobiology, Faculty of Natural Sciences, University of Haifa). Briefly, human iPSCs from a patient with PD were grown in mTesR™ plus medium (05825; Stem Cell Technologies, Canada) until the culture reached ~80% confluency. The iPSC colonies were then dissociated into a single-cell suspension using Accutase™ (A1110501; Thermo Fisher Scientific) for 5 min, followed by the addition of trypsin inhibitor (03-048-1C; Sartorius, Germany) to stop the dissociation. The dissociated cells were then re-plated on Matrigel (3433-010; R and D systems, USA)-coated plates in mTesR™ plus media at a density of 40,000 cells/cm^2^. After two days of daily media changes, cells reached ~50% confluency. At this point (Day 0), the differentiation process was initiated using KSR media-DMEM F-12 (11320033; Thermo Fisher Scientific), with Glutamax 1X, 15% KO-SR (10828028; Thermo Fisher Scientific), 1% NEAA (11140050; Thermo Fisher Scientific), 1% antibiotic-antimycotic (15240096; Thermo Fisher Scientific), and 0.l mM β-mercaptoethanol (M6250; Sigma-Aldrich). The medium was then changed gradually to N2 medium-DMEM F-12 with Glutamax 1X, 1% N2 supplement (17502048; Thermo Fisher Scientific), 1% antibiotic-antimycotic (for Days 5 and 6: 75% KSR:25% N2; for Days 7 and 8: 50% KSR:50% N2; for Days 9 and 10: 25% KSR:75% N2).

Finally, the medium was changed to B27 medium on Day 11 (Neurobasal medium [21103049; Thermo Fisher Scientific], 2% B-27 supplement, 1% Glutamax, 1% antibiotic-antimycotic, 10 ng/ml BDNF, 10 ng/ml GDNF [45010; PeproTech], 1 ng/ml TGFβ3 [100-36E; PeproTech], 0.2 mM ascorbic acid [72132; Stem Cell Technologies], and 0.1 mM cAMP [1141; Tocris UK]). During the differentiation process, the following small molecules were also added to the culture medium: 10 M SB431542 (13031; Cayman Chemical, USA) on Days 0–4; 100 nM LDN-193189 (1066208; BioGems, USA) on Days 0–12; 2 M purmorphamine (10009634; Cayman Chemical), 0.25 M SAG (11914; Cayman Chemical) and 100 ng/ml FGF8b (PeproTech, Israel, 100-25) on Days 1–6; 3 M CHIR99021 (13122; Cayman Chemical) on Days 3–12. Half the media was changed every other day. After 20 to 25 days of differentiation, neurons were dissociated for the second time using Accutase™ and trypsin inhibitor (as described above), re-plated onto Matrigel-coated 48-well coverslips and allowed to differentiate in B27 medium until day 30. From day 30 onward, the basal medium was gradually replaced with Brainphys™ (05790; Stem Cell Technologies, Canada) medium (instead of DMEM-F12), which helps in the formation of synaptic connections.

### Assessing the impact of PEG tail length on cellular uptake of liposomes in hCMEC/D3 and TF targeting capacity

Briefly, hCMEC/D3 cells were seeded (75,000 cells/ml) one day before the experiment. On the day of the experiment, PEG2000-TF/PEG1000 and PEG2000/PEG1000 Cy5-labeled liposomal formulations were prepared as described previously. Additional, PEG2000-TF/PEG2000 and PEG2000 Cy5-labeled liposomal formulations were prepared in molar percentages of 64.6 (DPPC):30 (cholesterol):2.5 (PEG2000-TF):2.5 (PEG2000):0.4 (Cy5-DSPE) and 64.6 (DPPC):30 (cholesterol):5 (PEG2000):0.4 (Cy5-DSPE), respectively. The hCMEC/D3 cells were incubated for 30 min with the different Cy5-labeled liposomal formulations to a final concentration of 0.5 mM total lipids, corresponding to ~ 10^12^ liposomes/ml. Then, the culture medium was removed, and cells were rinsed with PBS three times, incubated with Trypsin-EDTA for 4 min in heating, and centrifuged at 500*×g* for 7 min. Finally, the cell pellet was resuspended in fresh medium supplemented with 5% (v/v) FBS. Cellular uptake was detected in the Cy5 channel after acquiring 30,000 cells per sample using a flow cytometer (FACS; BD LSR-II, BD Biosciences, USA). Analyses were performed using FCS Express (De Novo software).

Likewise, we evaluated the effect of the amount of TF conjugated to liposome surface on cellular uptake in hCMEC/D3. Five different formulations of Cy5-labeled BTL (empty) were prepared with TF concentrations ranging from 1–20 mg/ml. TF protein conjugation to liposomes was described previously. The amount of TF mixed with the liposomes varied in each formulation, and labeled untargeted liposomes were used as a negative control (Figure S8).

### Super-resolution imaging of BTL cellular uptake in endothelial cells

Briefly, hCMEC/D3 cells were seeded (45,000 cells/ml) one day before the experiment. The following day, cells were overnight incubated with Cy5-labeled BTL (empty) to a final concentration of 2.5 mM total lipids, corresponding to 2.20×10^13^ liposomes/ml. Subsequently, the culture medium was removed, and cells were thrice rinsed with PBS and fixed with cold 4% paraformaldehyde (PFA) for 10 min. The cells were then permeabilized (0.1% Triton X100 for 5 min), blocked (10% FBS in 0.05% Tween20 in PBS for 30 min), and stained with Anti-Transferrin Receptor Antibody (ab84036; Abcam) at 5 μg/ml in blocking serum for 1 h. Next, cells were stained with Goat Anti-Rabbit IgG H&L conjugated Alexa Fluor 488 (ab150077; Abcam) at a dilution of 1:1000 in blocking serum for 1 h and then thrice rinsed with PBS (Figure S9). Acquisition and processing were performed using super-resolution (SR) microscopy (Elyra 7 eLS, Zeiss, Germany) and ZEN software, applying 405-, 488-, 561-, and 642-nm lasers.

### In-vitro BBB model of the NVU

BMECs were differentiated from iPSCs (BGU003 passage 16–18), as described by Neal et al. and Vatine et al.^[69–70]^ with the following modifications: iPSCs were seeded on Matrigel-coated plates (354234; Corning) at 20,800 cells/ml 24 h before differentiation. Differentiation was initiated by culturing cells in DMEM/F12 medium, supplemented with 20% knockout serum (10828010; Gibco), 1% non-essential-A amino-acids, 1 mM L-glutamine (25030149; Gibco), 216 μM β mercaptoethanol (31350010; Gibco), 100 U/ml penicillin, and 100 ug/ml streptomycin. The medium was changed daily for four days. Then, the medium was switched to endothelial serum-free medium (11111044; Gibco), supplemented with 20 ng/ml bFGF (10018B; Peprotech), 10 μM RA, B-27, 100 U/ml penicillin, and 100 μg/ml streptomycin, for 2 days, followed by re-seeding of cells on 3 μm pore Transwells (Greiner AG, Austria) coated with 400 μg/ml human collagen type IV (C5533; Sigma-Aldrich) and 100 μg/ml human fibronectin (356008; Corning), at least 4 h before seeding. Cells were seeded at 2×105 cells per 24-well Transwell. The following day, the medium was replaced with endothelial serum-free medium, supplemented with B-27, 100 U/ml penicillin, and 100 μg/ml streptomycin, exchanged every other day. The barrier function and density of cell layers were evaluated by TEER measurements (Millicell ERS-2 Voltohmmeter, Merck Millipore) daily. After ten days of differentiation, a TEER value of 154±9 ohm×cm^2^ was obtained, which was determined as an optimal value for the experiment.

For the liposome transport experiments, iPSC-derived BMECs cultured in Transwells were placed atop a primary cortical neuron culture transduced with a pAAV-hSyn1-EGFP-(P2A)-α-Syn A53T-HA tag viral vector. For control, Transwells without a layer of BMECs were used; liposome transport was faster on Transwells without a layer of BMECs.

For the live imaging experiments, 5 mM Cy5-labeled BTL (empty) was added to the donor chamber at different times (1, 4, 27, and 36 h, respectively). Then, BMECs were fixed for immunostaining of ZO-1 (cell signaling), and image acquisition was performed using a confocal microscope (Olympus IX-83) to allocate labeled liposomes at the Z position.

### Permeability of BTL across the in-vitro BBB model

To determine the permeability of BTL (empty) across the BBB, particles were prepared and added to the donor chamber at a concentration of 5 mM total lipids, corresponding to 1.05×10^13^ liposomes/ml. Medium samples (50 μl) were extracted from the acceptor chamber at 1, 2, 4, and 24 h and read using a plate reader at *λ_ex_*=633 nm and *λ_em_*=685 nm; as a chamber was available for each time point, replacement with fresh medium was not required. The cells were maintained under culture conditions during the transportation experiment. The liposome concentration was calculated using a calibration curve of Cy5-labeled BTL (empty), concentration versus intensity.

### Live imaging of BTL transport across the BMEC layer

A “Well-Chip” (Movie S2) was developed to image BTL transport, ensuring high-quality imaging and higher throughput. The “Well-Chip” was based on the concept of our previously reported chips, i.e., to create a modular system for imaging.^[71]^ The system was established using a polydimethylsiloxane (PDMS) sheet prepared with Sylgard 184 (761028; Sigma-Aldrich), mixed with 1:10 curing agent, followed by curing at 60C for at least 4 h. PDMS sheets were cut to fit a 12×60mm cover glass and punched with 3 holes (ID 10mm) to fit a Transwell assembly, as shown in the description of Movie S2. The cut PDMS sheets were cleaned in EtOH, dried at room temperature, activated in oxygen plasma (Atto-BR-200-PCCE; Diener Electronic, Germany) for 30 s, and assembled on the cover glass. A ring (outer diameter 9.5 mm, inner diameter 7.5 mm) was inserted within each “Well-Chip” to hold the Transwell at a specific height, designed in SolidWorks CAD software (SolideWorks Corporation, USA), and printed in a Form3 3D printer (Formlabs, Somerville, USA) using clear resin. Custom “Well-chips” were sterilized by washing with EtOH and irradiating with UV light for 30 min.

Following platform preparation, Transwells were added to the “Well-Chip”, and the Cy5-labeled BTL were added to the upper side of the Transwell along with seeded BMECs. BTL transport was examined using an Olymus IX-83 confocal microscope for 20 h in Z-stack. Time-lapse image analysis of BTL at different membrane levels was performed using ImageJ software (National Institutes of Health, Bethesda, MD).^[67]^ The lowest level was z=1 below the cells, the highest level was z=70 above the cells, and the membrane level was z=40 (Figure S11c).

### Evaluation of the endolysosomal pathway of BTL in brain sections

Three 7–8-week-old female C57/6JOlaHsd mice (Envigo, Israel) were deeply anesthetized (8.5 mg/ml ketamine, 1.5 mg/ml xylazine, in 100 μl saline) and injected retro-orbitally with 50 μl of AZDye 647-labeled BTL (empty) (50 mM; 1.79E+13 particles/ml). The particles were allowed to circulate for 30 min or 6 h. Brains were harvested, dissected, fixed in 4% PFA at 4°C overnight, cryopreserved in 30% sucrose, and frozen in TissueTek OCT (Sakura, Japan). Frozen brains were cut into 6 μm slices for dSTORM imaging (CM1950, Leica, Germany) to produce coronal brain sections. Then, slices were mounted on poly-D-lysine coated coverslips (no. 1.5H; Marienfeld-Superior, Germany). dSTORM imaging was performed using freshly prepared imaging buffer containing 50 mM Tris (pH 8.0), 10 mM NaCl, and 10% (w/v) glucose with an oxygen-scavenging GLOX solution (0.5 mg/ml glucose oxidase) (G2133; Sigma-Aldrich), 40 μg/ml catalase (C40; Sigma-Aldrich), 10 mM cysteamine MEA (30070; Sigma-Aldrich), and 1% ß-mercaptoethanol.^[72–74]^ A Nikon Ti-E inverted microscope was used for examination. The Nikon STORM system (N-STORM) was built on TIRF illumination using a 1.49 NA 100× oil immersion objective and an ANDOR DU-897 camera. Tissue sections were incubated with the following primary antibodies: Rabbit GLUT1 Polyclonal antibody (#07-1401; Millipore, USA) at a 1:400 dilution and Rat LAMP1 Monoclonal antibody (1D4B; DSHB, USA) at a 1:100 dilution. For secondary antibody staining, an Anti-Rat IgG Alexa fluor-488 (712-545-153; Jackson, USA) and an Anti-Rabbit IgG CF568 (20098; Biotium, USA) were used at dilutions of 1:400 and 1:800, respectively. Activation was achieved with 488, 568, and 647 laser lines, with a cycle repeat of ~4000 cycles for each channel. Nikon NIS Element and ThunderSTORM (NIH ImageJ)^[75]^ software were used for image acquisition and analysis. We used the dSTORM approach based on labeling the target protein with a primary antibody, followed by a secondary antibody conjugated to a fluorophore. Thus, resolved signals represent a location approximately 40 nm from the actual epitope (assuming the approximation of the two antibodies’ length in a linear conformation). A resolution of approximately 20 nm allowed signal separation and the application of these as proxies for the abundance of target molecules, which can reliably be used to compare different states. Single-molecule localization microscopy results in point patterns with specific coordinates of individual detected molecules. These coordinates are typically summarized in a ‘molecular list’ (provided by ThunderSTORM analysis).^[75]^ Colocalization analysis to determine the proximity of BTL to the LAMP1 marker (lysosomes) was performed using ImageJ. To avoid underestimating lysosomal localization, colocalization was defined as a distance <100 nm between liposomes and lysosome markers. A total of 15 capillaries were analyzed from three mice.

All experimental mice were maintained in the animal facility of the Hebrew University under specific pathogen-free conditions. All animals were treated according to institutional guidelines approved by the Institutional Animal Care and Use Committee (IACUC) at Hebrew University (Protocol MD-21-16361-5).

### Studying the spatial and temporal dynamics of BTL penetration into neuronal brain cells

*In utero* electroporation was performed on E14.5 timed pregnant ICR dames. The lateral ventricle of embryos was injected with a plasmid encoding CAG-mEGFP at a concentration of 1 μg/μl with 0.01% Fast Green dye. Five electrical pulses (45 V, 50-ms duration, 1 Hz) were delivered using a NEPA21 electroporator (NEPAGENE). Following birth, GFP-positive pups were identified and left to mature until p30. For cranial window surgery, mice were anesthetized with isoflurane, placed in a stereotaxic frame, and the skin and skull were exposed. A dental drill was used to perform a 2.5–3 mm circular craniotomy centered over the GFP-positive region. The skull was sealed using a cover glass, which was secured and attached to the exposed skull with a head plate fixing the head during imaging using dental cement. Mice were left to recover on a heating pad. After 2pFLIM imaging, mice were intravenously administered 350 μl 50 mM of Cy3-labeled BTL (1.68×10^13^ particles/ml) via the tail vein. For *in-vivo* 2pFLIM imaging, GFP, and Cy3 were excited using a Ti: sapphire laser (Chameleon, Coherent) at a wavelength of 920 nm and a power of 10–30 mW. Fluorescence lifetime images were obtained using a Bergamo two-photon microscope (Thorlabs) equipped with a time-correlated single photon counting board (Time Harp 260, Picoquant). Emission was collected with a 16×0.8 NA objective (Nikon), divided with a 565-nm dichroic mirror (Chroma), and detected with two photo-multiplier tubes with low transfer time spread (H7422-40p; Hamamatsu). Images were collected at 128×128 or 256×256 pixels, acquired at 2 ms/line, and averaged over 24 frames. The fluorescence lifetime of GFP and Cy3 was measured by curve fitting using custom software written with C#, as described previously.^[50]^ For analysis, TauD and TauAD values for GFP and Cy3 were fixed at 2.6 and 1.0 ns, respectively. A double exponential fit was calculated for regions of interest (ROI) on cell bodies and neurites. The difference in lifetime was calculated between a corresponding Cy3-labeled BTL-positive puncta and a nearby GFP background region (Figure S12). All animal experiments were approved by the Tel Aviv University Committee on Animal Care.

### Construction of a pAAV-hSyn1-eGFP-P2A-AS A53T-HA tag target plasmid

We next constructed an AAV vector exploiting the human synapsin-1 promoter to drive the expression of eGFP and alanine 53 to a threonine mutant AS linked by a self-cleaving P2A peptide, using A53T mutation by Inverse PCR applying primers hSynuclein A53T.FOR (5’–ACAACAGTGGCTGAGAAGACC- 3’) and hSynuclein A53T.REV (5’–CACACCATGCACCACTCCC – 3’) and a lentiviral plasmid pCMV-eGFP-wild type AS-HA tag as a template. Next, the HA-tagged A53T mutant AS gene was subcloned by Gibson assembly into NcoI and HindIII sites of plasmid pAAV-hSyn1-eGFP-P2A-eGFPf-WPRE-HGHpA (74513; Addgene, USA), applying lentiviral plasmid pCMV-eGFP-α-Syn-Puro A53T mut-HA tag as a template and the forward and reverse primers Syn-NcoI-gib.FOR (5’ – TGAAACAAGCAGGGGATGTCGAAGAGAATCCCGGGCCAGCCATGGATGTATTCATG AAAGGACTTTCAAAGG – 3’) and Syn-HindIII-gib.REV (5’ – TCTTTCACAAATTTTGTAATCCAGAGGTTGATTATCGATAAGCTTTTAAGCGTAATCT GGAACATCGTATGGG – 3’). Plasmid integrity was validated using DNA sequencing analysis (Figure S13).

### AAV production and infection of primary neurons

An AAV delivery system was used to express the eGFP-P2A-aplpha-synA53T-HA fusion protein in mouse primary cortical neurons. ProAAV HEK-293T cells were transfected with helper plasmids PHP.eB, pADdeltaF6, PHP.S (103005, 112867, and 103006, respectively; Addgene) and with a target plasmid pAAV-hSyn1-eGFP-P2A-α-synA53T-HA tag bearing the eGFP-P2A-α-synA53T-HA fusion protein under control of the human synapsin promoter. Briefly, ProAAV HEK-293T packaging cells growing in 15-cm dishes were transfected with a mix of 20 μg helper vector PHP.eB/PHP.S, 20 μg helper vector pADdeltaF6, and 20 μg target plasmid: pAAV-hSyn1-eGFP-P2A-α-synA53T-HA. JetPI transfection reagent (101-10N; Polyplus, Paris, France) was used as a transfection reagent under poor nutrients media: 1% Glutamax, high glucose DMEM. Eighteen hours post-transfection, the culture media was replaced with a fresh rich-serum medium containing 2% penicillin/streptomycin, 20% FBS, 1% glutamax, and high glucose DMEM. After 48 h of incubation, ProAAV HEK cells were detached by 0.5 M EDTA, and AAV particles were extracted, purified, and concentrated according to the AAVpro® Purification Kit Maxi (6666; TaKaRa, Japan). Quantitative PCR for AAV titer measurement was performed by applying SYBR green fastmix (95073-250; Quantabio, USA), forward ITR primer 5’-GGAACCCCTAGTGATGGAGTT, and reverse ITR primer 5’-CGGCCTCAGTGAGCGA. Readouts were compared to a calibration curve of a known concentration of an AAV backbone plasmid. Primary mouse cortical neurons were infected with α-synA53T AAV particles overnight. The culture medium was replaced with a fresh medium, and neurons were further incubated for seven days without changing the medium (Figure S13).

### Confocal imaging of BTL cellular uptake in PD primary neurons

Confocal microscopy (LSM 710; Zeiss, Germany) was performed to examine liposomal uptake and antibody payload release in PD primary neurons (Figure 3B). Overexpressed AS PD primary neurons were prepared using AAV developed with HEK293T (ATCC) cells, using high-titer AAV2 virions pseudotyped with RepCap-DJ packing, pAdenohelper, and a pAAV-hSyn1-EGFP-(P2A)-α-Syn A53T-HA tag (Figure S13). Briefly, cells were seeded (500,000 cells/ml) and then transduced with the engineered AAV virus in their growth media on day 3; the entire medium was replaced on day 4. On day 8, cells were treated either with free Cy3-labeled SynO4 antibody (3.95 μg/ml) or with 2.5 mM Cy5-labeled BTL loaded with Cy3-labeled SynO4 overnight. The following day, the medium was removed, and the cells were washed (3× with PBS), fixed (4%PFA for 10 min), permeabilized (0.25% Triton X100), blocked (5% Normal Goat Serum [S-1000; Vector Laboratories Inc, USA] with 1% bovine serum albumin [BSA] [0332-TAM; VWR Chemicals, USA], and immunostained with Rabbit Monoclonal Anti-Alpha-Synuclein (ab212184; Abcam) and Goat Anti-Rabbit CF568 (1:500) (20801; Biotium, USA); the cells were stained with Hoechst (1 μg/ml) for nuclei labeling. The entire procedure was carried out at the U. Ashery Lab. Imaging was performed at the LSE Infrastructure Center (Technion). The acquisition was performed using ZEN software and 405-, 488-, 543-, and 639- nm lasers.

### FACS quantification of BTL uptake in vitro

Seven days before experimentation, SH-SY5Y cells were seeded (120,000 cells/ml) and treated with RA and BDNF reagents to ensure full differentiation. Wells were transduced for 24 h with AAV1/2-CMV/CBA-human-A53T-alpha-synuclein-WPRE-BGH-polyA vector in a titer of 5×10^11^ GC/ml (PD-induced cells). On day 2, the PD cell medium was removed, and some wells were treated overnight with Cy3-labeled SynO4 mAbs (4.06 μg/ml) or Cy5-labeled BTL loaded with Cy3-labeled SynO4 mAbs (2.5 mM); fresh medium was added to the untreated wells. On day 3, the medium from all wells was removed, and wells were washed with PBS (3× times); cells were then dissociated with cell dissociation buffer mixed with 10% FBS solution. The buffer was centrifugated at 500 ×*g* for 7 min. Next, cell pellets were resuspended in a 5% FBS solution. The cells were then analyzed using a spectral flow cytometer (Cytek Aurora, CYTEK, USA), with 100,000 events counted per sample. The results were analyzed using FCS Express software. The results (1 independent repetition performed in 5 replicates) are presented as mean± standard deviation (SD). Two-tailed unpaired Student’s t-test was used for the statistical analysis; ***p<0.0001.

### SR imaging of BTL cellular uptake in differentiated PD-SH-SY5Y cells

Seven days before experimentation, cells were seeded (75,000 cells/ml) and treated with RA and BDNF reagents for full differentiation. Next, cells were incubated with 2.8 μg/ml recombinant human AS protein aggregates (active) (ab218819; Abcam) labeled with an Alexa Flour 488 Conjugation Kit (ab236553; Abcam) for 6 h to establish PD neurons. The culture medium was then removed, and cells were rinsed with PBS (3 × times) and incubated overnight with Cy5-labeled BTL loaded with Cy3-labeled SynO4 (2.5 mM), free Cy3-labeled SynO4 (3.65 μg/ml), or Cy5-labeled BTL (empty) (2.5 mM, control). The following day, the medium was removed, and the cells were rinsed with PBS (3 × times) (Figure S14a and b) and stained with Hoechst staining (1 μg/ml) (63493; Sigma-Aldrich) for nuclei labeling in both experiments. Image acquisition and processing were performed using SR microscopy (Elyra 7 eLS, Zeiss, Germany) and ZEN software, applying 405-, 488-, 561-, and 642-nm lasers.

### Quantitative imaging analysis of in-vitro studies

The confocal images of PD primary neurons were analyzed using the IMARIS software. An analysis was conducted to measure the amount of SynO4 antibody within each neuron, normalized by the number of neurons per field (at least 9 fields of 3 independent repetitions). Furthermore, SR images (Figure S14c and d) of PD-SH-SY5Y were analyzed using the IMARIS software, allowing semi-automated tracing of SynO4 antibody colocalization with AS aggregates,along with the percentage of SynO4 antibody released from BTL. The examined parameters and statistics were measured for at least 18 fields of three independent repetitions.

### Confocal imaging of BTL cellular uptake in PD-SH-SY5Y cells

Seven days before experimentation, cells were seeded (120,000 cells/ml) and treated with RA and BDNF reagents for full differentiation. Next, certain wells were incubated with 0.2 μM recombinant human AS protein aggregates (active) labeled with an Alexa Flour 488 Conjugation Kit for 6 h to establish PD neurons; the remaining wells were left untreated (representing a healthy control group). The culture medium was then removed, and cells were thrice rinsed with PBS and incubated overnight with Cy3-labeled BTL loaded with Cy5-labeled SynO4 (2.5 mM), free Cy5-labeled SynO4 (4.25 μg/ml), or Cy5-labeled BTL (empty) (2.5 mM, control). The following day, the medium was removed, and cells were rinsed with PBS (3× times) and stained with Hoechst staining (1 μg/ml; 63493; Sigma-Aldrich) for nuclei labeling in both experiments. Imaging was performed at the LSE Infrastructure Center (Technion). Image acquisition was achieved using ZEN software and by applying 405-, 488-, 543-, and 639-nm lasers (Figure S15).

### dSTORM microscopy

Initially, healthy primary and PD primary neurons transduced with the engineered AAV virus (day 3) were seeded (500,000 cells/ml). On day 8, the cells were incubated overnight with a free SynO4 antibody (3.95 μg/ml) and BTL (2.5 mM). The following day, the medium was removed, the cells were washed (3× with PBS), fixed (4% PFA for 10 min), permeabilized (0.25% Triton X100), blocked (5% Normal Goat Serum with 1% BSA), and immunostained with Rabbit Monoclonal Anti-Alpha-Synuclein and Goat Anti-Rabbit CF568.

### SR acquisition

dSTORM, SR imaging was performed using a single-molecule localization microscope (Vutara 350, Bruker). The defined precision of Vutara 350 is 20 nm XY and 50 nm Z resolution, with the lateral resolution between 15–40 nm and the axial resolution between 50–80 nm. It should be noted that we did not employ Z-stack imaging, given that we were imaging the sample at one Z plane. The custom case of Vutara 350 is designed for SR, environmental isolation, temperature regulation, and drift minimization.

Bruker’s patented Biplane technology offers higher localization precision than the astigm-atismbased method over a larger axial range, making it the preferred commercial 3D SR technique. Compared with the astigmatism-based method, the Biplane technique offers an enhanced per-pixel SNR, resulting in superior localization precision. The Biplane and Quadfield modules detect the PSF from two different focal planes and sum the total number of photons, yielding superior localization precision over a larger axial range (without the perceived loss of photons). sCMOS camera (4 MP, 6.5 m × 6.5 m pixel size for super-resolution imaging) and a CCD camera (1392×1040 for widefield imaging) were employed. Imaging was performed using a water immersion 60× objective NA 0.13-0.21/FN26.5. and 1000 mW lasers. To enable single-molecule photoswitching Alexa Fluor 647 and CF®568 dyes, the chamber was filled with imaging buffer B (50 mM Tris-HCl pH 8.0, 10 mM NaCl, 10 % (w/v) glucose), supplemented with 20 mM cysteamine (MEA; dissolved in buffer A [50 mM Tris-HCl pH 8.0, 10 mM NaCl]), 2% (v/v) Gloxy (glucose oxidase [168.8 AU] and catalase [1404 AU] mixture in buffer A), and 1% (v/v) 2-mercaptoethanol. Image processing was performed using the open-source software ImageJ^[76]^ and home-written analysis software, as detailed below.

### Cluster analysis

For each condition, 10 dSTORM images of 4 independent replicates were obtained randomly. A density-based clustering algorithm, Hierarchical Density-Based Spatial Clustering of Applications with Noise (HDBSCAN), was used to analyze the localization extracted from each image. We used a built-in module from the hdbscan-clustering library in Python. HDBSCAN determines the core distances for each localization to estimate its probability density function (PDF). The core distance of a point is its distance from its kth nearest neighbor; the denser the area, the smaller the core distance of the point. The parameters were as follows: minPoints = 50, and the extracting algorithm was ‘leaf. We applied noise reduction with principal component analysis with a standard deviation (SD) of 1.0. The PDF of a point is defined as the probability of being within a small region around it and can also be interpreted as the expected density around that point. Based on the mutual reachability distance, HDBSCAN assigns points to clusters. The mutual reachability distance of a pair of points is the maximum value between the core distance of point ‘a’, the core distance of point ‘b’, and the distance between points ‘a’ and ‘b’. Localizations of Connexin43 proteins in the dSTORM images were extracted as xy-coordinates and analyzed for cluster size, density, and number of localizations per cluster.

### FACS evaluation of cell death

Seven days before experimentation, SH-SY5Y cells were seeded (120,000 cells/ml) and treated with RA and BDNF reagents to ensure full differentiation. Wells were transduced for 24 h with either AAV1/2-CMV/CBA-human-A53T-AS-WPRE-BGH-polyA (GD1001RV; Charles River, USA) vector in a titer of 5×10^11^ GC/ml (PD-induced cells) or hydrogen peroxide (H_2_O_2_; 0.2M, positive control), or were left untreated (healthy, negative control). On day 2, the PD cell medium was removed, and some wells were treated overnight with free SynO4 mAbs (0.042 μg/ml), Cy5- labeled BTL (empty) (0.05 mM) or Cy5-labeled BTL (0.05 mM); fresh medium was added to the remaining wells. On day 3, the medium from all wells was collected, and cells were dissociated with the cell dissociation buffer (13151014; Rhenium). The buffer was re-united with the medium and centrifugated at 500×*g* for 7 min. Then, the cell pellets were twice washed with PBS and resuspended in a binding buffer. Subsequently, 5 μl Annexin V-FITC was added to the cell suspensions for 15 min in the dark, with 2.5 μl PI added during the last 5 min. The cells were then analyzed with a spectral flow cytometer (Cytek Aurora, CYTEK, USA), with 30,000 events counted per sample. The results were analyzed using FCS Express software. Annexin V-FITC and PI double-positive cells were necrotic or late apoptotic cells. The experiments were performed using a MEBCYTO Apoptosis Kit (Annexin V-FITC Kit) (4700; Enco, Israel).

### AAV-based PD mice model establishment

Healthy 6–8-week-old c57BC/6JRccHsd male mice (Envigo, Israel) were anesthetized with 0.5% isoflurane and 1% O_2_. Mice were administered a unilateral stereotactic injection of rAAV vector encoding AS-AAV2/6-hSyn1-Human SNCA-WPRE-polyA (SBSAA30004; Sirion Biolabs, Germany). Next, mice were injected with 1.5 μl rAAV (1.63×10^13^ GC/ml) directly to the right-side substantia nigra using an automated stereotactic injection device equipped with the mouse brain atlas; the machine was provided by the A. Zeisel lab, Technion. The well-being of all treated animals was monitored for over 8 weeks. After 2, 4, and 8 weeks of viral injection, mice were sacrificed, and immunohistochemical and western blot analyses were conducted (Figure S17). All animal experiments were approved by the Inspection Committee on the Constitution of the Animal Experimentation at the Technion (IL0010120 and IL0330222) and conducted according to its stipulated regulations.

### Western blot analysis

Two weeks post-PD induction, frozen brain sections of mice were divided into right and left hemisphere samples and homogenized in lysis buffer. The initial homogenization was performed using a gentle MACS Dissociator (Miltenyi, Germany), followed by sonication on ice. Next, samples were centrifuged at 15,000 *×g* for 1 h at 4°C. The supernatant was termed “triton-soluble”; the pellet was resuspended in lysis buffer with 2% sodium dodecyl sulfate (SDS) and termed “triton-insoluble”. Subsequently, the protein concentration was determined using a Bradford Protein Assay Kit (5000201; BIO-RAD, Israel). Next, 200 μg of protein extract was used to run an SDS-PAGE protein gel. Finally, a western blot was performed using an Anti-Alpha-Synuclein antibody (MJFR1) (ab138501; Abcam) and goat Anti-rabbit IgG-H&L (ab6721; Abcam). Given that anti-human AS antibody does not react with mouse AS, the western blot results refer solely to the human AS originating from the viral expression (Figure S17d).

### Reverse transcription-quantitative PCR (RT-qPCR) analysis

Eight weeks post-PD induction (via injection with 1.5 μl rAAV [1.63×10^13^ GC/ml] directly to the right-side substantia nigra), 6–8-week-old mice administered PBS injection, no injection were sacrificed and perfused with ice-cold PBS. The brains were frozen using liquid nitrogen and maintained at -80°C for further use. The outer sections of the BBB area were peeled off using a scalpel and frozen in liquid nitrogen. The sections were ground to a powder in liquid nitrogen using a mortar and pestle. Total RNA was extracted using a NucleoSpin RNA Plus kit (MAN740955; Ornat, Israel) in accordance with the manufacturer’s instructions. The purity and quantity of extracted RNA were evaluated using a plate reader, while its integrity was assessed using gel electrophoresis (2% agarose, 35 min at 100 V). Next, 400 ng of RNA was converted to cDNA using a qScript cDNA Synthesis kit (95047500; Agentek, Quanta BioSciences, Israel), following the manufacturer’s protocol. Lastly, quantitative real-time PCR (qRT–PCR) was performed using qPCRBIO SyGreen Blue Mix Lo-ROX (Tamar LTD., Israel, PB201551) with cycling conditions implemented according to the manufacturer’s instructions.

The RT-qPCR was performed using a QuantStudio1 (Applied Biosystems) real-time PCR thermal cycler. Then, the relative TfR1 expression was calculated using the 2^–ΔΔCt^ method. Before operating the RT-qPCR, specific primers (TF forward: AAACACAGACGTGCTCCATCA reverse: TCCTGCGTCCACTTTTGTCAT, and GAPDH forward: TGGGTGTGAACCACGAGAAA reverse: GGGCCATCCACAGTCTTCTG) were tested for their specificity (by analyzing dissociation curves ranging from 60–95°C), optimal concentration, and amplification efficiencies using standard no template and no enzyme controls.

### In-vivo biodistribution

Eight weeks post-PD induction (via injection with 1.5 μl rAAV [8.15×10^12^ GC/ml] directly to the right-side substantia nigra), 6–8-week-old mice administered PBS injection, or no injection were sacrificed and perfused with ice-cold PBS and healthy mice were intravenously injected (350 μl) either with Cy5-labeled untargeted liposomes or with Cy5-labeled BTL (empty); non-injected mice were used as a negative control. Twelve hours post-injection, mice were euthanized and perfused with PBS, and their brains, lungs, livers, kidneys, hearts, and spleens were extracted and imaged by IVIS.

### IVIS imaging

Biodistribution was performed using IVIS imaging. Except for the brains, extracted organs were imaged *ex vivo* at an excitation of 640 nm and emission of 680 nm, binning of 4, and f-stop of 2, with 1-s for Cy5-labeled liposome detection (Figure S18). *Ex vivo* brain images were obtained at an excitation of 640 nm and emission of 680 nm, binning of 4, and f-stop of 2 and 5-s parameters. The Cy5-labeled BTL (empty) and Cy5-labeled untargeted liposomes were also imaged at 640 nm excitation and 680 nm emission, 4 binning, 2 f-stops, lasting 5 and 1 s, respectively. Quantitative data from all images were analyzed using the ROI tool in Living Image software. A control (non-injected) mouse was used for analysis, with average radiance subtracted from the average radiance of each injected tissue, respectively. To compare the BTL (empty) mice group and the untargeted liposomes mice group, the radiance values of each tissue were normalized to the respective mean radiance value of each liposomal formulation.

### Analysis of SynO4 antibody levels in the whole brain using an IgG1-based ELISA assay

Approximately 4–5 weeks post-PD induction (via the injection of 1.5 μl rAAV [1.63×10^13^ GC/ml] directly to the right-side substantia nigra), mice were randomly separated into three treatment groups: Cy5-labeled BTL loaded with Cy3-labeled SynO4 (5.59×10^12^ particles/ml), free Cy3-labeled SynO4 (158.5 μg/ml), and non-injected. The mice were intravenously injected (150 μl) with the selected treatment and sacrificed 24 h post-injection. The mice were then perfused with PBS, and their brains were frozen using liquid nitrogen and kept at -80°C for further use. Fresh frozen brain tissues were crushed into a fine powder using a pestle and mortar submerged in liquid nitrogen. The frozen powder was then transferred to Ripa Lysis buffer (RIPA; 50 mM Tris-HCL, 150 mM NaCl, 1 mM NP-40, and 0.5 mM sodium deoxycholate), supplemented with phosphatase and protease inhibitors (0.4 mg/ml Collagenase Type 4 [LS004186; Worthington, USA], 0.08 mg/ml Collagenase Type 1 [SCR103; Sigma-Aldrich], and 0.1 mg/ml DNase 1 [AMPD11KT; Sigma-Aldrich]). After further homogenization, the lysate was centrifuged for 30 min at 12,000×g and 4°C. A supernatant containing the entire brain protein lysate was used for the experiment. The protein concentration was measured using the Bradford assay. A protein lysate was produced from each group (i.e., BTL, free SynO4, and no treatment), and 500 μg/ml of total protein from each group was used for the ELISA. Given that the SynO4 antibody is a mouse-origin IgG1 isotype, the detection was performed using an anti-mouse IgG1 ELISA assay kit (ab133045; Abcam), according to the manufacturer’s protocol.

### Fluorescence immunohistochemistry analysis

Fluorescence immunohistochemistry was used to visually verify the presence of BTL in the substantia nigra area of the brain. Approximately 4–5 weeks post-PD induction (via the injection of 1.5 μl rAAV [1.63×10^13^ GC/ml] directly to the right-side substantia nigra), mice were randomly administered one of three treatments: intravenous injection of Cy5-labeled BTL loaded with Cy3-labeled SynO4 (5.59×10^12^ particles/ml), intravenous injection of free Cy3-labeled SynO4 (158.5 μg/ml), and non-injected. The mice were sacrificed 24 h after treatment administration. Finally, mice were perfused with PBS, and the brains were frozen using liquid nitrogen and maintained at -80°C for further use. Then, 16 μm sections of fresh frozen brain tissue (substantia nigra area) were cut using a cryostat machine. The frozen slices were mounted with DAPI Fluoromount-G (010020; ENCO, Israel), covered-slipped, and dried overnight at 4°C. The slides were imaged the following day using confocal microscopy.

Image processing, manipulation, and analysis were conducted using Python. Several steps were taken to improve image quality and quantify color overlap. First, a median filter with a kernel size of 5 was applied to reduce noise and create smoother images. Subsequently, unsharp masking was used to enhance contrast and sharpen edges. The unsharp masked image was generated by subtracting the blurred image from the median filtered image with a weight of 4. Second, channel extraction was performed on the unsharp masked image to separate the red and green color channels. These channels contained pertinent information for quantifying the overlap between respective colors. To determine this overlap, a bitwise AND operation was executed on red and green channels, resulting in isolated regions where colors overlapped. Individual contours were identified by converting these overlap regions into binary images and utilizing the OpenCV library. The number of contours corresponded directly to the number of overlapping colors within each image was analyzed. Finally, all measurements collected from each image were compiled into a data frame and exported as an Excel sheet for further analysis (Figure S19).

### TF-SynO4 antibody conjugation

Human holo-TF was cross-linked to SynO4 mAbs using EDC and Sulfo-NHS reagents. First, 1 mg/ml SynO4 antibody solution (PBS), 15 mg/ml Sulfo-NHS (PBS), and 15 mg/ml EDC solution (DMSO) were prepared. Second, the reagent solutions were added to the antibody solution at the following molar proportions: 1:25 SynO4:Sulfo-NHS and 1:10 SynO4:EDC. The pH of the SynO4 solution was adjusted to 6 using 1.2 M HCL. Then, the solution was mixed (450 rpm) at 25°C for 1 h. To remove excess reagents, the SynO4 solution was dialyzed against PBS (pH 6.0; 1:1000 volume ratio) using a 12–14 kDa dialysis membrane at 4°C overnight. Next, TF-SynO4 antibodies were synthesized by mixing 1 mg/ml TF solution (PBS) with an activated SynO4 antibody solution at a 2:1 molar proportion, respectively. Then, the pH of the TF-SynO4 solution was adjusted to 8.4 using 1 M sodium bicarbonate, with the reaction incubated (450 rpm) for 2 h at 25°C. Non-conjugated TF was removed by dialysis against PBS (pH 7.4; 1:1000 volume ratio) using a 300 kDa dialysis membrane (131456; Repligen, USA) at 4°C for 1 week; the buffer was exchanged twice daily. Finally, a Micro BCA Protein Assay Kit was used to quantify the final concentration of the TF-SynO4 antibody solution.

To prepare Cy5-labeled TF-SynO4 mAbs, a volume of Cy5-NHS dye was added to a known concentration of TF-SynO4 solution, 10:1 molar proportion, respectively. Then, the pH of the Cy5-labeled transferrin-SynO4 solution was adjusted to 8.4 using 1 M sodium bicarbonate, and the reaction was incubated (450 rpm) for 2 h at 25°C. Finally, the Cy5-labeled TF-SynO4 solution was dialyzed against PBS (pH 7.4; 1:1000 volume ratio) using a 12–14 kDa dialysis membrane at 4°C overnight to remove excess Cy5 dye.

### In-vivo FACS analysis of liposome uptake at the whole brain and single-cell level

Eight weeks post-PD induction (via the injection of 1.5 μl rAAV [8.15×10^12^ GC/ml] directly to the right-side substantia nigra), mice were intravenously injected with 350 μl of Cy5-labeled SynO4 (45 μg/ml), Cy5-labeled transferrin-SynO4 (45 μg/ml), or Cy5-labeled BTL (empty) (5.84×10^12^ particles/ml); healthy mice were intravenously injected with Cy5-labeled BTL (empty); and non-treated mice were used as a negative control. Twelve hours post-injection, mice were sacrificed and perfused with ice-cold PBS, and their brains were extracted and suspended in cold PBS. The brain tissues were cut into small pieces and enzymatically and physically dissociated using a gentleMACS device (Miltenyi Biotec; it has built-in heating programs provided by S. Shen-Orr lab, Technion) and an Adult Mouse Brain Dissociation Kit (mouse and rat) (130107677; Almog diagnostics, Israel), according to the kit’s dissociation protocol.

The dead cells were removed from cell samples using a Dead Cell Removal Kit (130090101; Almog Diagnostics), according to the manufacturer’s instructions. The live-single-cell suspensions from each treated brain were divided into “stained” and “non-stained” cells; both groups contained Cy5 dye. The live-single-cell suspensions were divided into “non-stained” and “stained” cells. The “stained” cells were obtained after incubation with a panel of antibodies: PE Anti-Mouse/Human CD44 (BLG-103008), Brilliant Violet 711™ Anti-Mouse CD45 (BLG-103147), Brilliant Violet 421™ Anti-Mouse CD31 (BLG-102424), PE/Cyanine7 Anti-Mouse/Human CD11b (BLG-101216), Anti-Mouse CD24 Antibody Clone M1/69 Alexa Fluor® 488 (60099AD), and ACSA-2 Antibody Anti-Mouse APC-Vio770 REAfinity (130-116-247) (all antibodies were from Almog diagnostics). The dilution of each antibody was determined according to the manufacturer’s instructions. The cells were incubated with antibodies for 30 min on ice in the dark. Subsequently, cells were washed with PB buffer (0.5% BSA in PBS) and resuspended with phosphate buffer before reading. All cell groups were analyzed using Cytek Aurora; an Anti-Rat and Anti-Hamster Ig k/negative control compensation particles set (552845; BD, USA) was used for single staining. A minimum of one million cells were recorded for each test sample. The analysis was performed using FCS Express software (Figure S20). The “non-stained” cells of treated brains were used to calculate the average percentage of Cy5-positive cells (Figure 4F). The “stained” cells of treated brains were used to calculate the average percentage of Cy5-positive cells within each cell type (Figure 4G). The “non-stained” cells of non-treated brains were used to calculate the average percentage of each cell type (cell brain population) (Figure S21).

### In-vivo FACS analysis of liposome uptake in PD dopaminergic neurons

Mice 8 weeks post-PD induction (via the injection of 1.5 μl rAAV (8.15x10^12^ GC/ml) directly to the right-side substantia nigra), mice were intravenously injected with 350 μl of Cy5-labeled BTL (9.83×10^12^ particles/ml) loaded with Cy3-labeled SynO4 mAbs (80 μg/ml); non-treated mice were used as a negative control. Twelve hours post-injection, mice were sacrificed and perfused with ice-cold PBS, and their brains were extracted and suspended in cold PBS. The brain tissues were cut into small pieces and enzymatically and physically dissociated using a gentleMACS device and an Adult Mouse Brain Dissociation Kit. Dead cells were removed from the cell samples using a Dead Cell Removal Kit. The live-single-cell suspensions from each treated brain were incubated with a panel of the antibodies: Brilliant Violet 510™ Anti-Mouse/Human CD44 (BLG-103044; Almog diagnostics), Brilliant Violet 711™ Anti-Mouse CD45, Brilliant Violet 421™ Anti-Mouse CD31, PE/Cyanine7 Anti-Mouse/Human CD11b, Anti-Dopamine Transporter (DAT) (extracellular)-FITC Antibody (AMT-003-F; Allomone labs, Israel), and ACSA-2 Antibody Anti-Mouse APC-Vio770 REAfinity; negative control samples of treated brains were incubated without the Anti-Dopamine Transporter (DAT) (extracellular)-FITC Antibody. The dilution of each antibody was determined according to the manufacturer’s instructions. The cells were incubated with antibodies for 35 min on ice in the dark. Then, cells were washed with phosphate buffer (0.5% BSA in PBS) and resuspended with phosphate buffer before reading. All cell groups were analyzed using Cytek Aurora; Comp Beads (424601; BioLegend, USA) was used for single staining. A minimum of one million cells were recorded for each test sample. Dopaminergic neurons are a rare group, comprising only a small number of approximately 21,000 cells in the midbrain of C57BL/6 mice^[77]^. To enhance the likelihood of identifying this population, the FCS files from all six samples were merged using the Cytek Aurora program before conducting the analysis (Figure S22).

### In-vitro cellular uptake of BTL in PD human dopaminergic neurons

Human dopaminergic neuronal cells were treated with 2.5 mM Cy5-labeled BTL particles in 500 μl media per well. This 48-h treatment was performed 45 days after patient derivation. Subsequently, neurons were fixed with 4% PFA at 37°C for 15 min, followed by thrice washing with PBS for 5 min each.

For immunocytochemistry^[78]^, fixed neurons on 48-well coverslips were treated with 0.2% Triton-X for 1 h to ensure permeabilization and then blocked using 10% horse serum in DPBS. The primary antibodies, mouse tyrosine hydroxylase antibody (ab129991; Abcam) at a 1:250 dilution and chicken beta-III-tubulin/TUJ1 (ab41489; Abcam) at a 1:1500 dilution, were applied to the cells and incubated. For secondary antibody staining, a Goat Anti-Mouse IgG (H&L)-488 (ab150117; Abcam) and a Goat Anti-Chicken IgY (H&L)-568 (ab175711; Abcam) were used at dilutions of 1:250 and 1:1000, respectively.

The coverslips were counterstained with DAPI using Fluoromount-GTM Mounting Medium (00-4959-52; Thermo Fisher Scientific, USA) and left to dry overnight, protected from light. The imaging process was conducted at the LSE Infrastructure Center (Technion) using ZEN software and lasers at 405, 488, 543, and 639 nm wavelengths. Confocal z-stacks were acquired for analysis. Additionally, antibody controls were treated under the same conditions, except incubation with primary antibodies was not performed (Figure 4H).

### In-vivo therapeutic efficacy

Briefly, mice received a unilateral stereotactic injection of 1.5 μL AAV vector encoding human AS at a concentration of 8.15×10^12^ GC/ml. The mice were randomly divided into the following four groups (4–5 mice per group): healthy (non-injected with viral vector), untreated (PD), PD with BTL recipients, and PD with free SynO4 mAb recipients. Cy5-labeled BTL (250 μl of 50 mM liposomes [0.5 mg/kg body weight of encapsulated antibody]) or SynO4 antibody (250 μl of 40 μg/ml solution [0.5 mg/kg]) were injected intravenously every other day for a period of two and four weeks, during the body weight of mice was monitored. Finally, mice were sacrificed and perfused, and their brains were harvested, fixed (PFA 4%, neutral-buffered, for at least 24 h), and further examined.

### Immunohistochemistry analysis

Fixed brain specimens were embedded in paraffin, and the substantia nigra and striatum (CP) areas were sectioned into 5-μm-thick slices. Next, tissue sections were deparaffinized in a xylene ethanol gradient (xylene, xylene/ethanol (1:1 v/v), absolute ethanol, 95% ethanol, 70% ethanol, and 50% ethanol) for 3 min each and then placed in distilled water. Antigen retrieval was performed in 10 mM tri-sodium citrate solution (pH 6.0) titrated with HCl. Ready-to-use normal goat serum (2.5%) was used for blocking. Next, sections were overnight incubated at 4°C with one of the following primary mAbs: Chicken Anti-Tyrosine Hydroxylase (1:1000) (ab76442; Abcam), Mouse-Anti-Aggregated Alpha-Synuclein Clone 5G4 (1:400) (MABN389; Mercury Scientific, Israel), or Rabbit Anti-Iba1 (1:2000) (ab178846; Abcam), and Rabbit Anti-GFAP (1:2000) (ab68428; Abcam). Next, tissue sections were rinsed with distilled water and then incubated for 30 min in 0.3% H_2_O_2_ solution to block endogenous peroxidase activity. The tissue sections were then washed in distilled water and incubated for 40 min (at room temperature) with either Goat Anti-Chicken IgY (HRP) (ab6877; Abcam), ImmPRESS Goat Anti-Rabbit IgG (HRP) (VE-MP-7451-50; Vector Laboratories, USA), or Goat Anti-Mouse IgG (HRP) antibody. Next, tissue sections were rinsed with distilled water (3× times); for color development, sections were incubated with DAB solution (SK4105 kit; Vector Laboratories, USA) for 2 min, washed with distilled water, and counterstained with hematoxylin. Staining AS aggregates first, followed by incubation with chicken anti-tyrosine hydroxylase for 1 h at room temperature, we could double-stain dopaminergic neurons (anti-tyrosine hydroxylase) and AS aggregates (5G4 clone) simultaneously (Figure S23). Immediately after AS staining, tissue sections were rinsed with distilled water and then incubated with the anti-chicken HRP antibody for 40 min (at room temperature). Next, tissues were thrice rinsed with distilled water and incubated with vector red solution (SK-5105 kit; Vector Laboratories) for 25 min for color development. Finally, the tissue sections were washed with distilled water and counterstained with hematoxylin.

All slides were scanned using a 3DHistech Panoramic 250 Flash III automated slide scanner. The area with the viable regions (annotations) was measured using CaseViewer software and used for further analysis.

### Imaging analysis of immunohistochemistry staining

Image processing was performed by the image analysis team at the Biomedical Core Facility, Technion. The analysis was conducted using Python, utilizing several images and data processing libraries, and based on several consecutive steps for each image. The first step of the analysis included color segmentation, where brown-colored areas were counted and compared with the overall area of the tissue. The next step consisted of structure segmentation based on a pre-trained convolutional neural network, where cells were detected and counted. Third, the colocalization of brown color expression and cell structure in the image was quantified for counting the brown color-expressed cells. Finally, the measurements for each image were collected as a data frame and exported as an Excel sheet for further analysis (Figure 5B, C, and D, and Figures S23, 24, 25, and 26). For the same brain section, the percentage of neuronal loss was calculated by dividing the number of dopaminergic neurons detected in the right hemisphere (viral-injected side) by the number of dopaminergic neurons located in the left hemisphere (non-viral injected side). Next, the values of the healthy group were normalized to 1, and the values of all the other groups were normalized to the mean value of the healthy group.

### Evaluation of behavioral changes in PD mice post-viral induction

Healthy 6–8-week-old male c57BC/6JRccHsd mice were maintained under a regular 12-h light/dark cycle (light phase: 7 AM to 7 PM) in a controlled environment (22°C, 50% humidity), with excess to water and food *ad libitum.* First, to establish an accurate model for behavioral testing, the mice were divided into three groups: healthy controls, mice injected with 8.15×10^12^ GC/ml of AAV viruses (concentration used in the previous biochemical experiments), and mice injected with 1.08×10^13^ GC/ml of AAV viruses. To evaluate coordination and balance functions, we measured the motor activity of experimental mice at 7, 21, and 35 days post-viral injection using an accelerating speed rotarod (Figure S28). The low concentration of AAV viruses did not significantly reduce the latency to fall, indicating a motor performance comparable with that of the healthy controls (Day 7 *p*=4942; Day 21 *p*=0.8232; Day 35 *p*=0.9952). Conversely, 35 days after the high viral concentration injection, the latency to fall was significantly reduced (*p*<0.0165). Accordingly, the high viral concentration was used in the behavioral experiments to test the therapeutic efficacy of BTL.

In the behavioral experiments, mice were randomly divided into four groups (7–8 mice each) as follows: healthy (non-injected with viral vector), PD untreated, BTL, and free SynO4 mAb. Cy5-labeled BTL (350 μl of 50 mM liposomes; 0.5 mg/kg body weight of encapsulated antibody) or free SynO4 mAbs (250 μl of 40 μg/ml solution; 0.5 mg/kg) were injected intravenously every other day for four weeks. The weight of the mice was monitored.

The rotor-rod™ apparatus (San Diego Instruments, San Diego, CA) comprised a black Perspex material featuring a horizontal rotating rod (5.5 cm diameter) with four lanes and opaque black Perspex dividers for each mouse. The apparatus also included a sawdust cabin (45.7 cm height) for safe landing. A red-beam system automatically recorded fall latencies (in seconds) to accurately assess the animal’s performance.

To assess the efficacy of the BTL when compared with that of free SynO4, we utilized the rotarod test, commonly used to evaluate motor function and learning in rodents.^[79–82]^ One-week post-viral injection, mice were handled daily (~6 min) and habituated to the experimenter and experimental room for a week. The rotarod test was conducted during the third and fifth post-injection weeks (after 2 and 4 weeks of treatment) by a trained experimenter blinded to the treatment conditions. The test was performed for three consecutive days during each experimental week, with four daily trials separated by a 2-min inter-trial interval. The experimental and control naive groups were evaluated between 9:00 AM-12:00 PM in a counterbalanced manner to avoid a testing order/time effect between groups. On day 1 of the experiment, each mouse was placed on the rod for 2 min, allowing habituation to the arena before initiating testing. During each trial, the initial rotation speed of the rod was 5 rpm for 15 s, which was gradually accelerated at a rate of 0.1 rpm/s up to a maximum speed of 50 rpm after 450 s. The latency to fall was recorded for each trial, and a mean was calculated for each mouse per testing day to provide a quantitative measure of the motor performance. The average latency of the first testing day served as an indication of motor functioning; the last testing day was used to represent the motor learning endpoint. All motor measurements were performed in a double-blind manner, and the identification of groups was revealed at the end of the experiment.

### Analyzing toxicity using immunohistochemistry

Fixed organ (liver, kidneys, and spleen) specimens were embedded in paraffin and sectioned into 5-μm-thick sections. Next, tissue sections were deparaffinized in a xylene ethanol gradient (xylene, xylene/ethanol (1:1 v/v), absolute ethanol, 95% ethanol, 70% ethanol, and 50% ethanol) for 3 min each and then placed in distilled water. Sections were stained with hematoxylin for nuclear stain, rinsed under tap water, and then stained with 1% eosin.

All slides were scanned using a 3DHistech Panoramic 250 Flash III automated slide scanner. The area of viable regions (annotations) was measured using CaseViewer software and used for further analysis.

### Hematology blood test

Blood samples were collected from mice four weeks after the behavior experiment, i.e., 40 days after the experiment initiation. The mice were sacrificed and terminally bled through a cardiac puncture. To analyze the chemistry parameters, blood samples were incubated on ice for 30 min, followed by centrifugation for 10 min at 10,000 rpm to separate the serum. A heparin-coated syringe was used to collect blood samples into EDTA-covered vials to prevent clot formation. An analysis of blood cell count and multiple chemistry parameters was conducted (American Medical Laboratories, Israel) to evaluate the potential systemic immunogenicity, inflammation, hepatotoxicity, and nephrotoxicity of BTL and free SynO4 treatment. All values were normalized to the healthy control group.

### Statistical analysis

All data were reported as the mean±SD). Comparisons were performed between distinct groups. Groups were analyzed by a two-tailed unpaired t-test, one-way analysis of variance (ANOVA), and two-way ANOVA. Statistical significance was set as *p<0.05, **p<0.01, ***p<0.001, and ****p<0.0001, with a 95% confidence interval. Analysis and figures were generated using GraphPad Prism v. 8.0 (GraphPad Software, Inc., La Jolla, CA, USA).

## Supplementary Material

Movie S1

Movie S2

Movie S3

Supplementary material

## Figures and Tables

**Figure:**
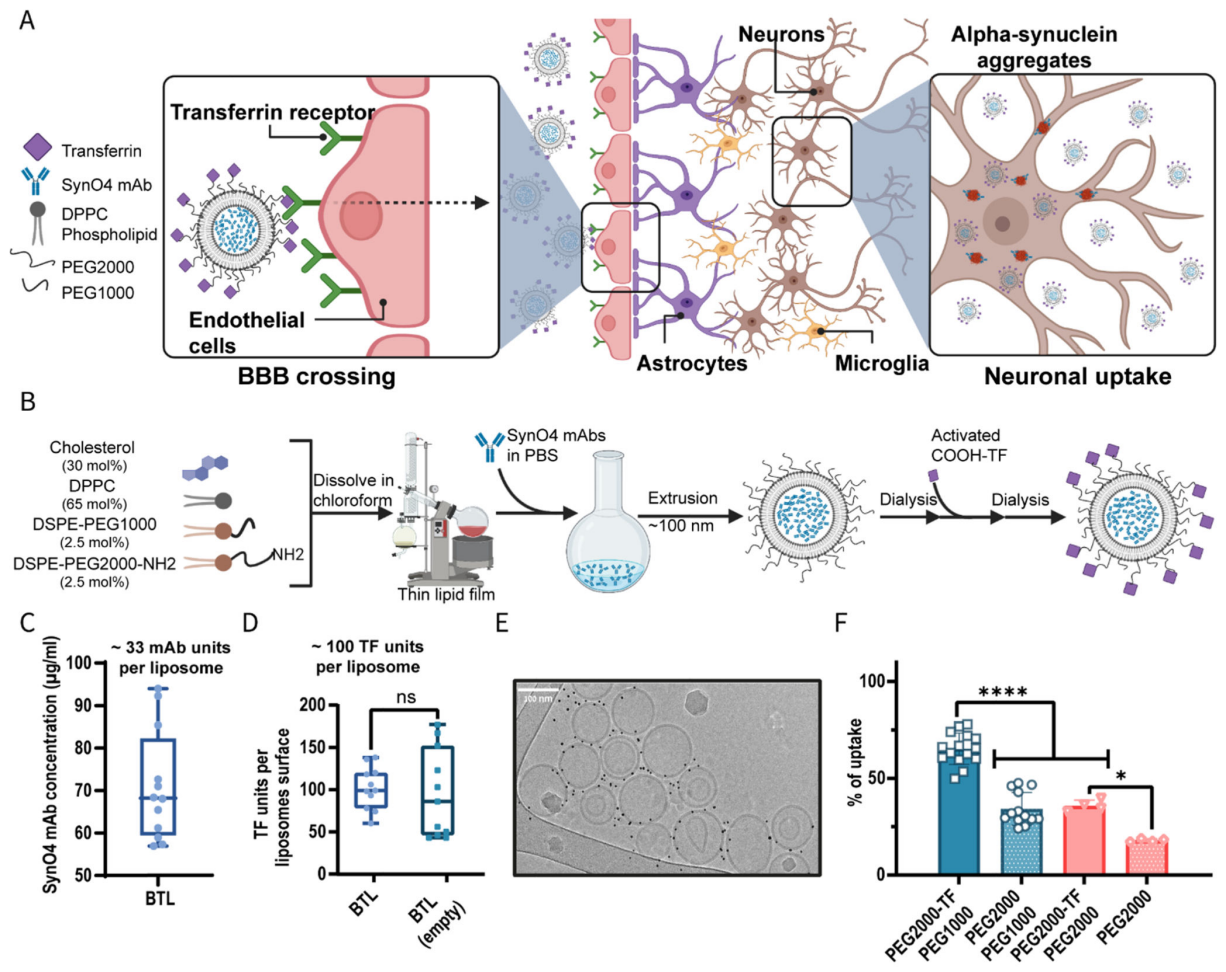
Synthesizing brain-targeted liposomes loaded with SynO4 mAb. (A). Schematic illustration of the therapeutic mode of action. Through receptor-mediated transcytosis, liposomes carrying SynO4 mAbs cross the BBB and are taken up by damaged neuronal cells; the mAbs are then released and target AS aggregates, thereby preventing neuronal cell death. (B). Schematic diagram of BTL synthesis. (C). Quantification of the encapsulated SynO4 mAb concentration in BTL using ELISA. (D). Evaluation of the number of transferrin units per liposome surface using the BCA protein assay. (E). Cryogenic transmission electron microscopy (cryo-TEM) of gold nanoparticles (GNPs) linked to BTL (empty) (scale bar: 100 nm). (F). In-vitro cellular uptake of targeted PEGylated liposomes in hCMEC/D3 cells; the uptake efficiency of each liposomal formulation was assessed by FACS analysis. The results of C and D (at least 12 independent repetitions) and F (at least 4 independent repetitions performed in three replicates) are presented as mean± standard deviation (SD). Two-tailed unpaired Student's t-test was used for the statistical analysis of D, and One-way ANOVA was used for the statistical analysis of F, with multiple comparisons test adjusted p-value; *p=0.0111, ****p<0.0001. AS, alpha-synuclein; BBB, blood-brain barrier; BTL, brain-targeted liposomes; DPPC, 1,2-dipalmitoyl-sn-glycerol-3-phosphocholine; ELISA, enzyme-linked immunosorbent assay; FACS, fluorescence-activated cell sorting; mAb, monoclonal antibody; NPs, nanoparticles; PBS, phosphate-buffered saline; PEG, polyethylene glycol; TF, transferrin.

**Figure:**
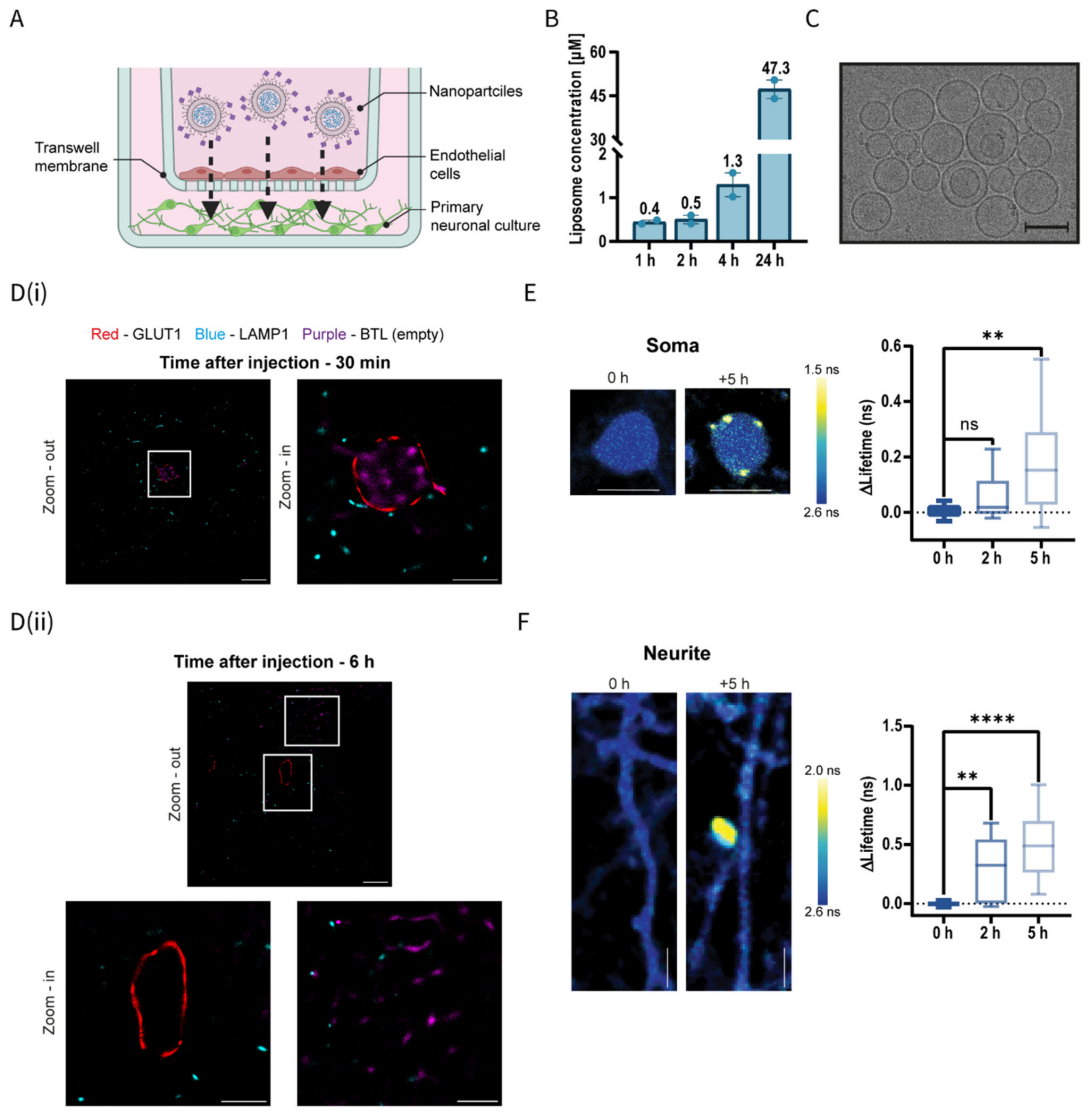
BTL cross the BBB. (A). Schematic diagram illustrating the penetration of BTL (empty) across an in-vitro BBB model comprising a co-culture of BMECs atop primary neurons placed in a noncontact manner in a Transwell. (B). Liposome concentration in neurons of the BBB model over time following application of Cy5-labeled BTL (empty) to the monolayer of BMECs, as determined by fluorescent measurement. The particle concentration increases over time, reaching 47.3±3.2 µg/ml after 24 h. (C). A Cryo-TEM image showing that liposomes remain intact after crossing the BBB (scale bar: 100 nm). (D). Tissue dSTORM images showing BTL crossing the BBB: (i) short time after injection and (ii) long time after injection. The liposomes were labeled with AZDye 647 (purple), capillaries were labeled with Alexa Fluor 488 (GLUT1; red), and lysosome molecules were labeled with CF568 (LAMP1; blue) (scale bars: zoom-out images 5 µm, zoom-in images 2 µm). Representative 2pFLIM pseudo-colored images and comparison analysis of the alteration in fluorescence lifetime of (E) soma and (F) neurite processes 0, 2, and +5 h after BTL injection, respectively. The liposomes were labeled with Cy3 (lower lifetime, yellow), and cells were labeled with GFP (higher lifetime, blue) (scale bars: soma images 10 µm, neurite images 5 µm). The results of B (1 independent repetition performed in 2 replicates), E (13–29 independent repetitions performed), and F (11–26 independent repetitions performed) are presented as mean±standard deviation (SD). One-way ANOVA with an adjusted p-value in multiple comparison tests was used for the statistical analysis; **p≤0.0012, ****p<0.0001. AS, alpha-synuclein; BBB, blood-brain barrier; BMECs, brain microvascular endothelial cells; BTL, brain-targeted liposomes; mAb, monoclonal antibody; Cryo-TEM, cryogenic transmission electron microscopy; GLUT1, glucose transport protein type 1; LAMP1, lysosome-associated membrane protein 1.

**Figure:**
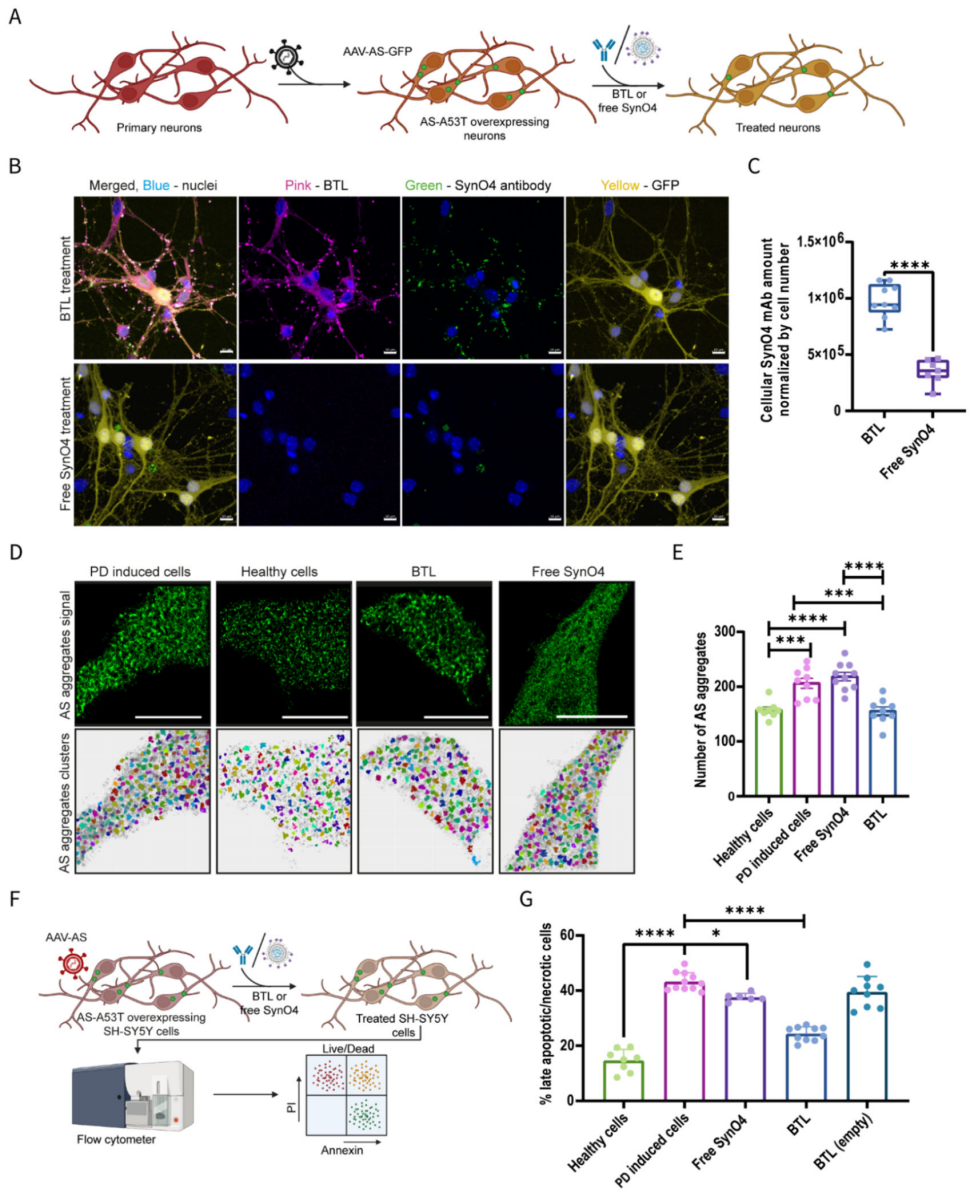
BTL are taken up by PD neurons and induce a therapeutic effect. (A). Schematic diagram illustrating the process of infecting PD primary neuronal cells with a viral vector overexpressing A535 alpha-synuclein, followed by treatment with BTL or free SynO4 mAbs. (B). Confocal images showing the uptake of BTL or free SynO4 mAbs in infected PD neurons after overnight incubation. Liposomes were labeled with Cy5 (pink), the antibody was labeled with Cy3 (green), and PD primary neuron cells were labeled with GFP (yellow) (scale bar: 10 µm). (C). Analysis of the cellular SynO4 mAb amount normalized to cell number by IMARIS imaging software. (D). dSTORM images of PD-infected neurons treated overnight with BTL or free SynO4 mAbs; neurons were marked with GFP (green) (scale bar: 9 µm). (E). Analysis of the number of AS aggregates using the HDCSCAN algorithm. (F). Schematic diagram illustrating PD-SH-SY5Y cells infected with a viral vector overexpressing A535 alpha-synuclein, followed by treatment with BTL or with free SynO4 mAbs and labeled with Annexin and PI dyes for a live/dead cell viability assay. (G). Quantification of the percentage of late apoptotic/necrotic cells following the five different treatments using FACS analysis. The results of C (3 independent repetitions performed; at least 6 images and at least 60 cells per image) and E and G (3 independent repetitions performed in at least 8 replicates each) are presented as mean±standard deviation (SD). One-way ANOVA with an adjusted p-value in multiple comparison tests was used for the statistical analysis; *p=0.0346, ***p=0.0003, ****p<0.0001. AAV, adeno-associated virus; AS, alpha-synuclein; BTL, brain-targeted liposomes; FACS, fluorescence-activated cell sorting; mAb, monoclonal antibody; PD, Parkinson’s disease.

**Figure:**
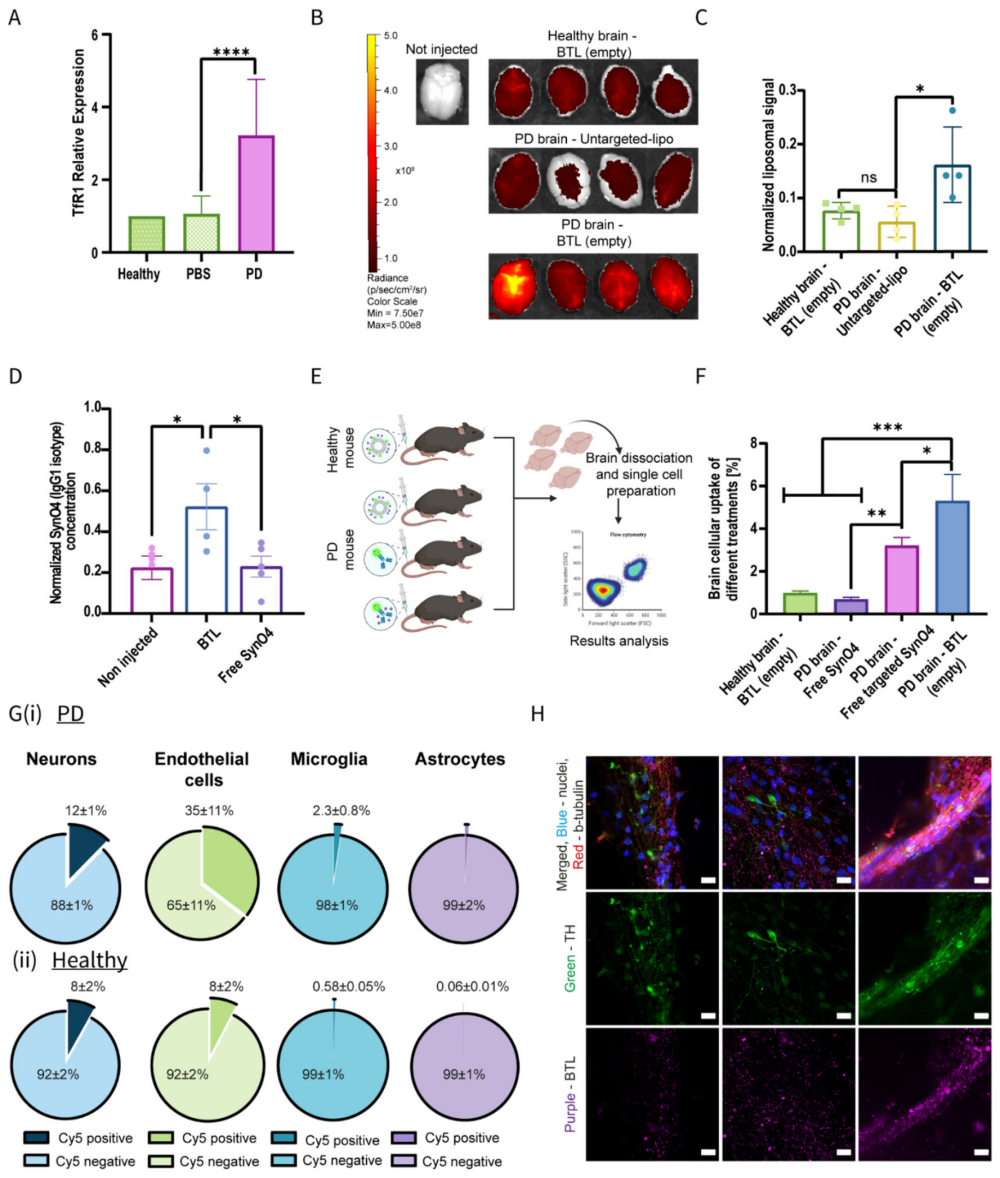
BTL cross the BBB and significantly accumulate in PD mice brains. (A). RT-qPCR analysis of TfR1 expression in the PD brain; TfR1 expression levels were normalized to those of the healthy group and were obtained according to the 2^(-∆∆Ct) method. (B-C). Nanoparticle biodistribution in the brains of PD-induced and healthy mice 12 h post-administration of Cy5-labeled BTL (empty) or Cy5-labeled untargeted liposomes, analyzed using an in-vivo imaging system (IVIS) (B) and quantified by IVIS software analysis (C). (D). Levels of the IgG1 isotype (SynO4 isotype) in PD brains following liposome delivery or antibody delivery, as determined using ELISA. (E). Schematic diagram illustrating the flow cytometry setup experiment. (F). The levels of BTL (empty), transferrin-SynO4 mAb, and free SynO4 mAb in PD brain cells and those of BTL in healthy brains were determined using flow cytometry. (G). Quantification of BTL cellular uptake in neurons, endothelial cells, microglia, and astrocytes in (i) PD brains and (ii) healthy brains. (H). Confocal imaging of BTL cellular uptake in human PD dopaminergic neurons. The liposomes were labeled with Cy5 (purple), and cells were stained with tyrosine hydroxylase (TH, green), b-tubulin (red), and nuclei (blue) (scale bar: 10 um). Results of A, C, and E (5 independent repetitions) and D and F (4 independent repetitions) are presented as mean±standard deviation (SD). One-way ANOVA with an adjusted p-value in multiple comparison tests was used for statistical analysis in A, C, D, and F; *p≤0.1061, **p≤0.0065, ***p≤0.0002, ****p<0.0001. BBB, blood-brain barrier; BTL, brain-targeted liposomes; PD, Parkinson’s disease. RT-qPCR, reverse transcription-quantitative PCR; TfR1, transferrin receptor.

**Figure:**
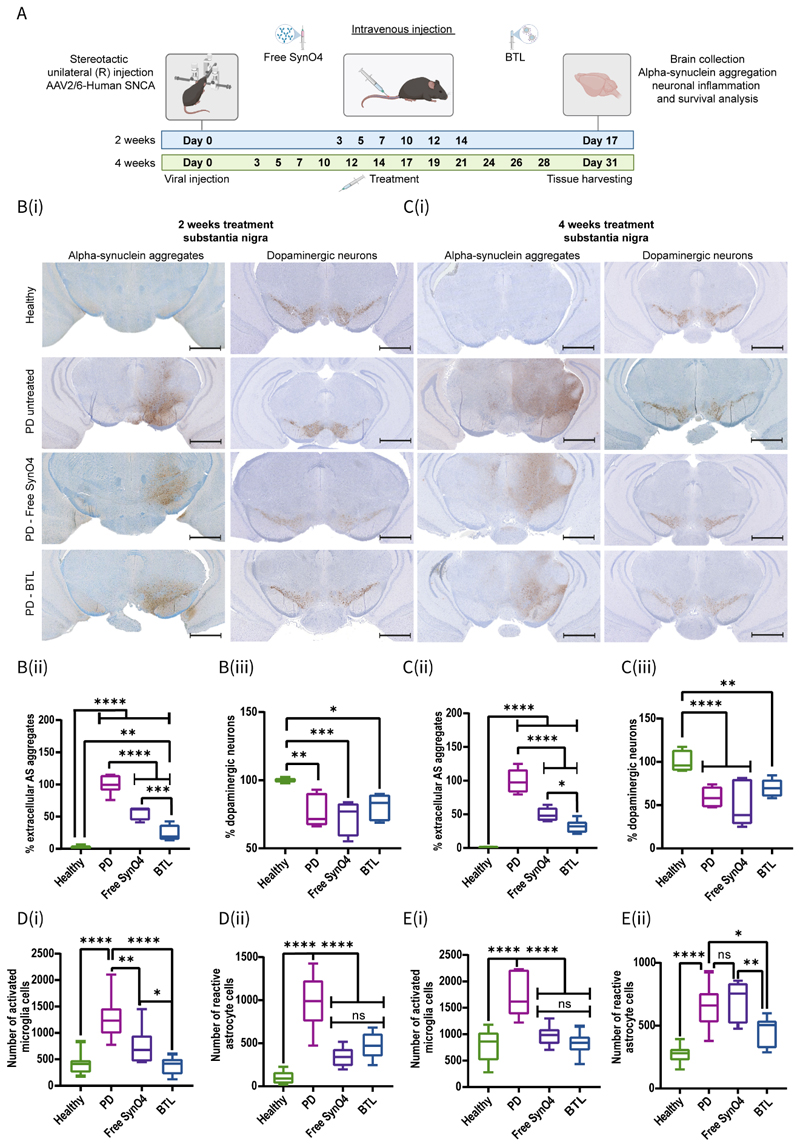
BTL reduce AS aggregation and neuroinflammation in the AAV-based PD mice model. (A). A schematic diagram illustrating the experiment to assess therapeutic efficacy: Healthy mice received unilateral AAV injection encoding human AS into the right hemisphere of the brain. Mice were injected every other day with the different treatments, i.e., free SynO4 mAbs or BTL, for two or four weeks. In the final stage, the brains were harvested, sectioned, and stained for biochemical and histological analysis and compared with the untreated PD group and healthy group (B(i), C(i)). Representative histological images of sections of the substantia nigra area after (B(i)) two weeks and (B(ii)) four weeks of treatment. Sections were stained against aggregated AS and dopaminergic neurons (scale bar: 2000 µm). (B(ii), C(ii)). The percentage of aggregated AS after (B(ii)) two weeks and (C(ii)) four weeks of different treatments. (B(iii), C(iii)). The percentage of dopaminergic neuron survival after (B(iii)) two weeks and (C(iii)) four weeks of different treatments. The healthy group values were normalized to 100%, and the values of other groups were normalized to the mean value of the healthy group. (D(i), E(i)). The number of activated microglia cells (D(i)) after two weeks and (E(i)) four weeks of different treatments; the sections were stained with an antibody against the Iba1 marker. (D(ii), E(ii)). The number of reactive astrocyte cells (D(ii)) after two weeks and (E(ii)) four weeks of different treatments; the sections were stained with an antibody against the GFAP marker. The results of B(ii), B(iii), D(i), D(ii), and E(ii) (3–4 independent repetitions in 1–3 technical replicates), C(ii) and E(i) (4–5 independent repetitions in 1–3 technical replicates) and C(iii) (3–5 independent repetitions in 1–3 technical replicates) are presented as mean±standard deviation (SD). One-way ANOVA was used for the statistical analysis in B, C, D, and E; *p≤0.0370, **p≤0.0072, ***p≤ 0.0004, ****p< 0.0001. AAV, adeno-associated virus; AS, alpha-synuclein; BTL, brain-targeted liposomes; mAbs, monoclonal antibodies; PD, Parkinson’s disease.

**Figure:**
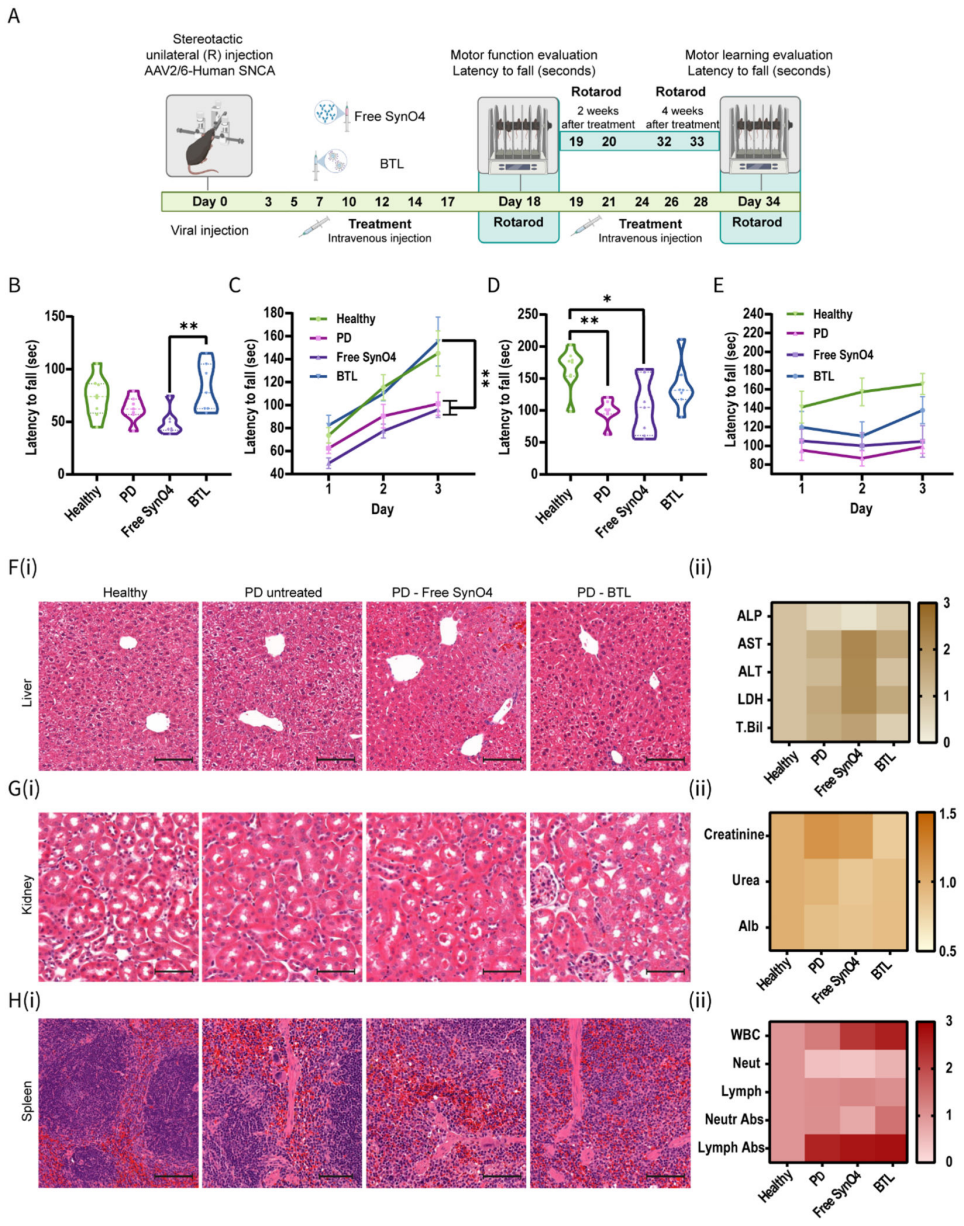
The capacity of BTL to prevent disease progression in a viral PD mice model. (A). Schematic diagram illustrating the behavioral therapeutic efficacy experiment: Healthy mice received a unilateral AAV injection encoding human AS. Then, the mice were injected every other day with either free SynO4 or BTL for 2 or 4 weeks. At the end of the treatment period, mice were measured on three consecutive days in an accelerating speed rotarod to detect coordination and balance functions. (B). Motor functioning capacity on the first day of rotarod evaluation after treatment for two weeks. (C). Short-term motor learning capability after treatment for two weeks. (D). Motor functioning capacity on the last day (day 3) of rotarod evaluation after treatment for four weeks. (E). Long-term motor learning after treatment for four weeks. Histological organ sections (F(i)) liver, (G(i)) kidney, and (H(i)) spleen on day 40 of the experiment. Sections were stained with hematoxylin and eosin to identify the cell structure. No differences in cell structure can be observed between healthy and BTL-treated groups in all the evaluated organs (scale bar: 100 µm). (F(ii)). Hepatotoxicity test of blood on day 40 of the experiment. Hepatic enzymes, including alkaline phosphatase (ALP), aspartate transaminase (AST), alanine transaminase (ALT), lactate dehydrogenase (LDH), and total bilirubin (T.Bill), were measured. No differences can be observed between the healthy and BTL-treated groups. The presence of free Syno4 results in increased levels of hepatic enzymes and bilirubin, indicating liver damage. (G(ii)). Assessment of nephrotoxicity using blood collected on day 40 of the experiment. Creatinine, urea, and albumin levels were measured. No differences can be observed between the healthy and BTL-treated groups. (H(ii)). Assessment of white blood cell count (WBC) using blood sample collected on day 40 of the experiment. No differences can be observed between the healthy and BTL-treated groups. All values are normalized to the values of the healthy group. Neut- Neutrophils, Lymph- Lymphocytes. The results of B and D (7–8 independent repetitions) are presented as mean±standard deviation (SD). One-way ANOVA was used for statistical analysis; *p≤0.0109 **p≤0.0049. Results of C and E (7–8 independent repetitions) are presented as mean±SD. Two-way ANOVA was used for statistical analysis; *p≤0.0106 **p≤0.0075. The results of F(i), G(i) and H(i) are representative sections of three independent repetitions performed in 1–3 technical replicates. The results of F(ii), G(ii) and H(ii) (three independent repetitions performed in 5 technical replicates) are presented in heat maps. AAV, adeno-associated virus; AS, alpha-synuclein; BTL, brain-targeted liposomes; PD, Parkinson’s disease.

## Data Availability

All the data needed to evaluate the conclusions in the paper are present in the paper and/or the Supplementary Materials.
