# Supplementary material for: Brain-targeted liposomes loaded with monoclonal antibodies reduce alpha-synuclein aggregation and improve behavioral symptoms in Parkinson’s disease
Source: Adv Mater. Author manuscript; Available in PMC 2024 Jan 4. (PMC7615408; doi:10.1002/adma.202304654)
Supplement: Supplementary material [file EMS190288-supplement-Supplementary_material.pdf]

ADVANCED MATERIALS

Supporting Information

for *Adv. Mater.*, DOI 10.1002/adma.202304654

Brain-Targeted Liposomes Loaded with Monoclonal Antibodies Reduce Alpha-Synuclein Aggregation and Improve Behavioral Symptoms in Parkinson's Disease

*Mor Sela, Maria Poley, Patricia Mora-Raimundo, Shaked Kagan, Aviram Avital, Maya Kaduri, Gal Chen, Omer Adir, Adi Rozencweig, Yfat Weiss, Ofir Sade, Yael Leichtmann-Bardoogo, Lilach Simchi, Shlomit Aga-Mizrachi, Batia Bell, Yoel Yeretz-Peretz, Aviv Zaid Or, Ashwani Choudhary, Idan Rosh, Diogo Cordeiro, Stav Cohen-Adiv, Yevgeny Berdichevsky, Anas Odeh, Jeny Shklover, Janna Shainsky-Roitman, Joshua E. Schroeder, Dov Hershkovitz, Peleg Hasson, Avraham Ashkenazi, Shani Stern, Tal Laviv, Ayal Ben-Zvi, Avi Avital, Uri Ashery, Ben M. Maoz and Avi Schroeder**

Supplementary Information

Brain-targeted liposomes loaded with monoclonal antibodies reduce alpha-synuclein aggregation and improve behavioral symptoms in Parkinson's disease

Mor Sela, Maria Poley, Patricia Mora-Raimundo, Shaked Kagan, Aviram Avital, Maya Kaduri, Gal Chen, Omer Adir, Adi Rozencweig, Yfat Weiss, Ofir Sade, Yael Leichtmann-Bardoogo, Lilach Simchi, Shlomit Aga-Mizrachi, Batia Bell, Yoel Yeretz-Peretz, Aviv Zaid Or, Ashwani Choudhary, Idan Rosh, Diogo Cordeiro, Stav Cohen-Adiv, Yevgeny Berdichevsky, Anas Odeh, Jeny Shklover, Janna Shainsky-Roitman, Joshua E. Schroeder, Dov Hershkovitz, Peleg Hasson, Avraham Ashkenazi, Shani Stern, Tal Laviv, Ayal Ben-Zvi, Avi Avital, Uri Ashery, Ben M. Maoz and Avi Schroeder

*Corresponding author.

This PDF file includes:

Figure S1 to S29

Movie S1 to S3

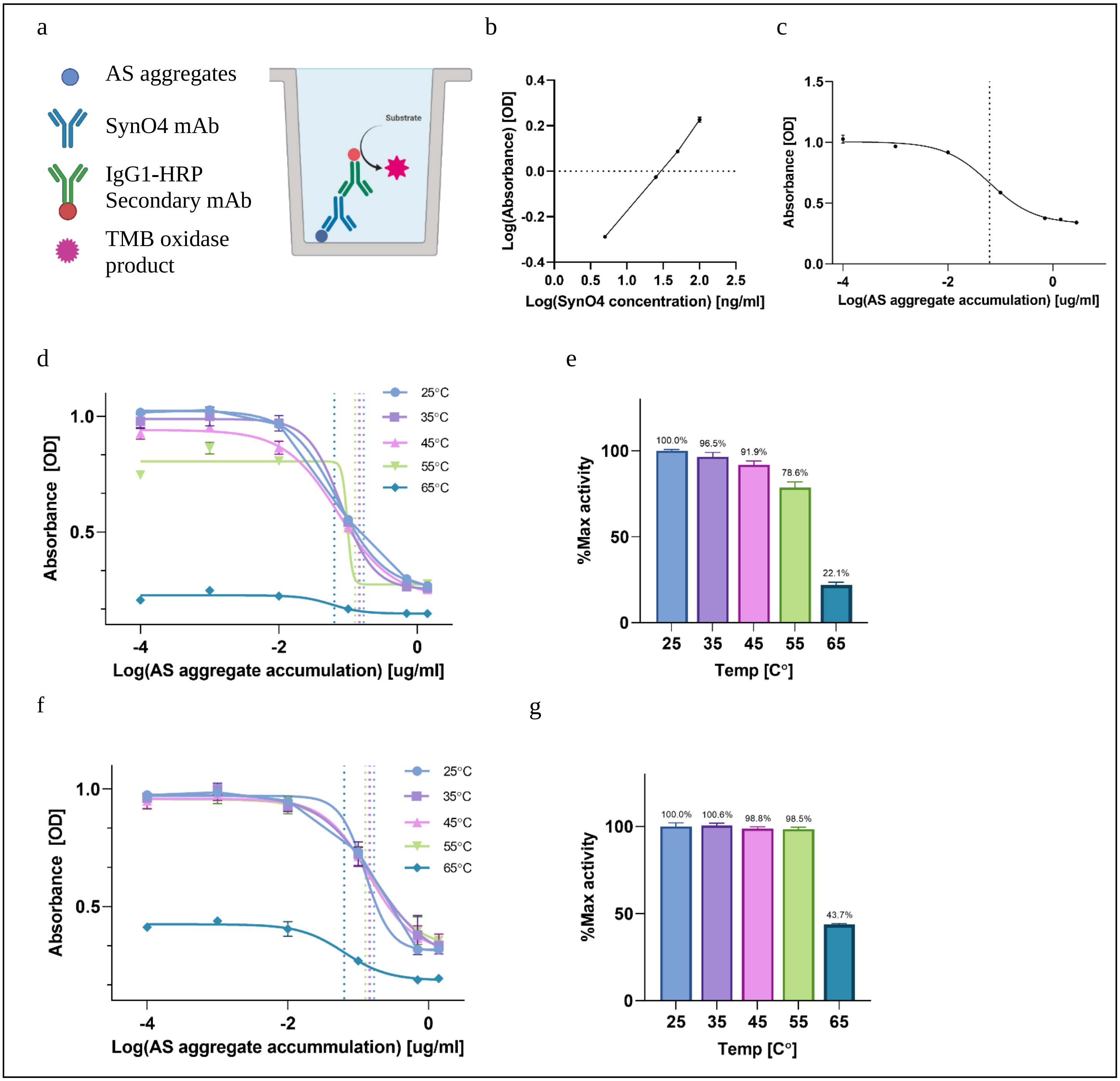

Figure S1. SynO4 antibody activity. **(a)** Schematic illustration of a direct ELISA assay. (**b**) Calibration curve of known SynO4 antibody concentrations. (**c**) IC50 curve of SynO4 antibody inhibition by AS aggregates (for 25 ng/ml of SynO4 mAbs IC50=0.06 µg/ml). (**d**) SynO4 antibodies were pre-treated at different temperatures (35, 45, 55, and 65°C) for 1 h and immediately measured by ELISA to create an IC50 curve. (**e**) Maximal activity of SynO4 antibodies (**f**) SynO4 antibodies pre-treated at different temperatures (35, 45, 55, and 65°C) for 1 h, stored overnight at 4°C, and the day after measured by ELISA to create an IC50 curve. (**g**) The maximal activity of SynO4 antibodies after overnight at 4°C. Results (3 independent repetitions in 3 replicates in each experiment) are presented as mean±standard deviation (SD).

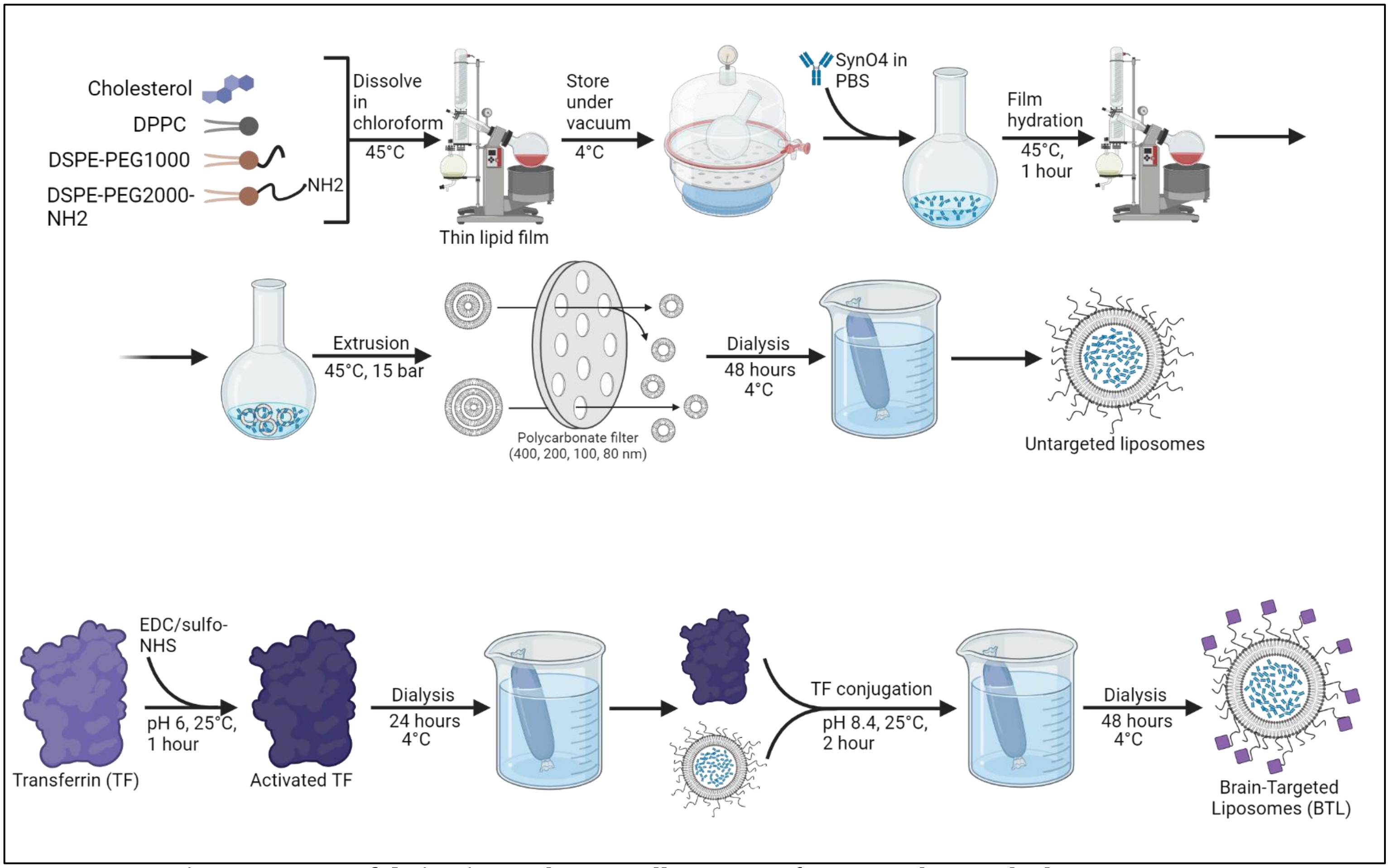

Figure S2. BTL fabrication. Schematic illustration of BTL synthesis. The liposomes were prepared using the thin-film method. Next, they were extruded and the non-encapsulated SynO4 antibodies were removed by dialysis. Then, transferrin protein was cross-linked to the surface of the liposomes by amide linkage reaction. Lastly, non-conjugated transferrin was removed by dialysis.

	Average diameter [nm]	PDI	Zeta potential [mV]	Particle concentration [particles/ml]	Drug loading rate [(μmole antibody/ μmole lipid) min^{-1}]
**BTL**	113.5±1.5	0.162±0.010	-28.2±0.1	8.49E+12	1.50E-8
**SynO4 loaded liposomes**	114.3±0.3	0.165±0.025	-29.1±1.3	6.67E+12	2.56E-8
**BTL (empty)**	119.4±0.7	0.092±0.006	-25.5±1.2	5.84E+12	-

Figure S3. Physical parameters. Average diameter, PDI, zeta potential, and particle concentration of the three liposomal formulations. The values were measured by Zetasizer Ultra (Malvern Analytical).

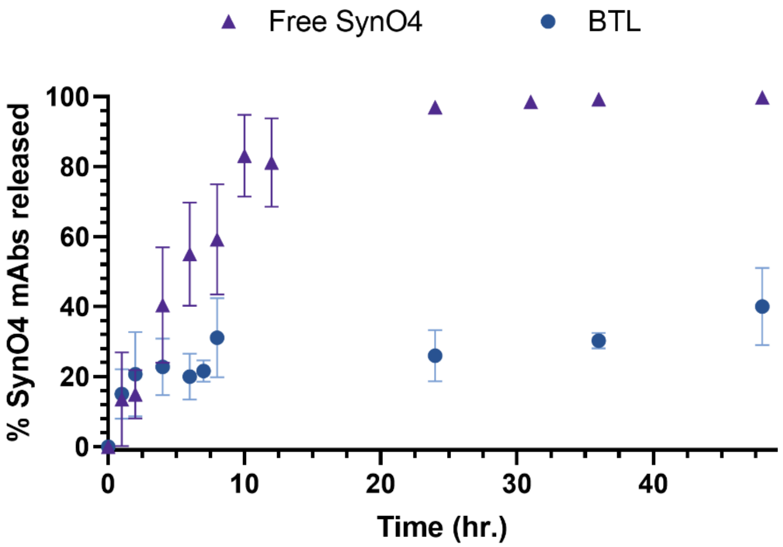

Figure S4. ***In-vitro*** **release profiles.** In-vitro drug release profiles of BTL compared to free SynO4 mAbs in PBS over 48 h, at 37ºC. Results (3 independent experimental repetitions performed in 3 replicates in each experiment) are presented as mean±standard deviation (SD).

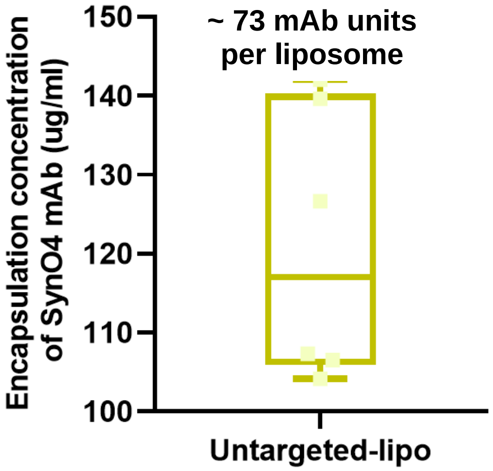

Figure S5. SynO4 mAb encapsulation concentration (EC) in untargeted liposomes. The EC was measured by ELISA assay. The results (6 independent experimentalrepetitions) are presented as mean±standard deviation (SD).

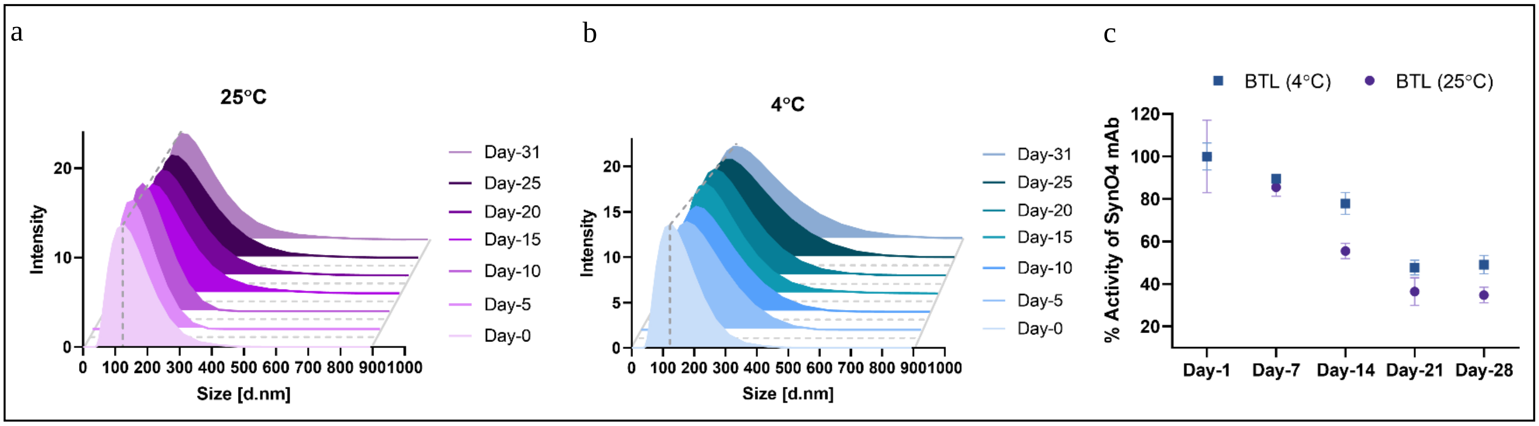

Figure S6. Stability testing of BTL. (a-b) The particles' stability was coherent for 31 days at (a) 25°C and (b) 4°C; no significant changes in size were measured. **(c)** The percentage activity of encapsulated SynO4 mAbs within BTL was measured using ELISA. As can be gleaned from the graph, the antibody exhibited stability when stored at 4°C for a duration of 14 days or at 25°C for one week. The hydrodynamic diameter data of BTL in a and b is presented as a waterfall graph. The results of c (1 independent experimental repetition) are presented as mean±standard deviation (SD).

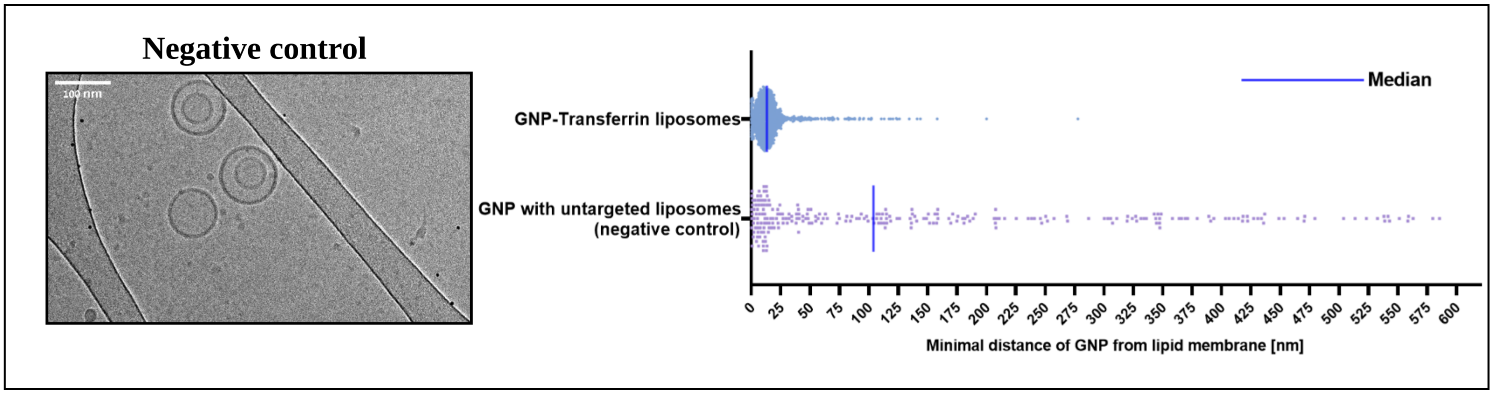

Figure S7. Cryo-TEM imaging. (Left) Representative Cryo-TEM image of gold nanoparticles (GNP) with untargeted liposomes (negative control) (scale bar: 100 nm). **(Right)** Fiji imaging software analysis, a scatterplot of GNP-liposomes. Results represent 1 independent experimental repetition performed in more than 1000 replicates.

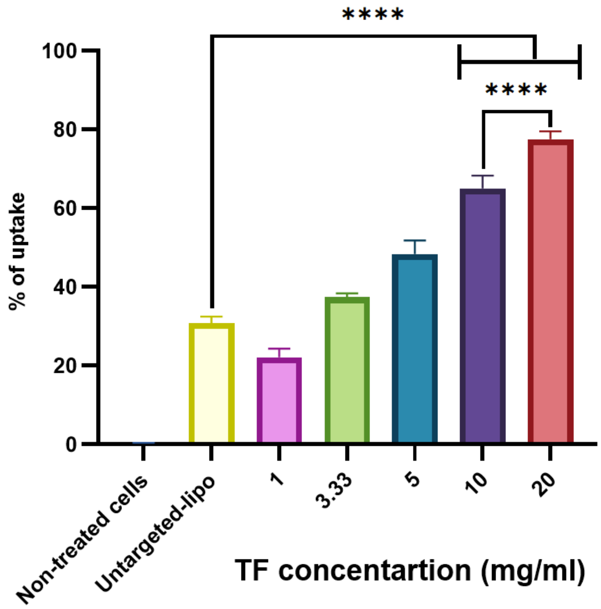

Figure S8. Effect of the number of TF moieties on the liposome uptake by endothelial cells. FACS analysis of the cellular uptake by hCMEC/D3 cells of liposomal formulations prepared with different transferrin concentrations. The average number of TF units conjugated per liposome was determined as follows: TF was undetectable in concentrations of 1 mg/ml, 3.33 mg/ml, and 5 mg/ml due to the protein absorbance spectra value being insufficiently sensitive and falling outside the detection assay range. At a concentration of 10 mg/ml, the measured number of TF units conjugated per liposome was 95±23, and in the concentration of 20 mg/ml, 109±11. Results (2 independent experimental repetitions performed in 4 replicates) in each experiment are presented as mean±standard deviation (SD). One-way ANOVA was used for the statistical analysis with multiple comparisons test adjusted p value; *p<0.0001.

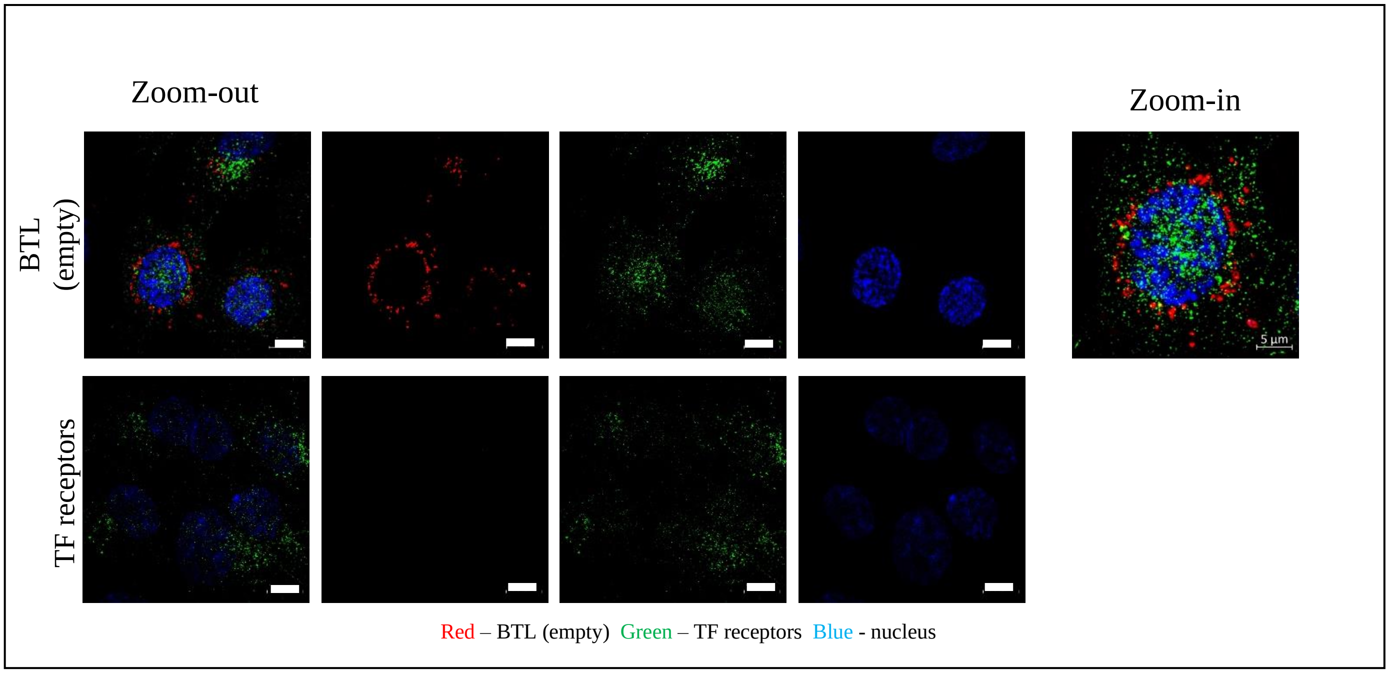

Figure S9. BTL cellular uptake by hCMEC/D3 cells. Super-resolution images of the cellular uptake of BTL (empty) by brain endothelial cells (hCMEC/D3). hCMEC/D3 cells stained only with anti-TF receptor antibodies were used as negative control. Scale bar: 10 µm for the zoom-out images and 5 µm for the zoom-in image in the right top corner.

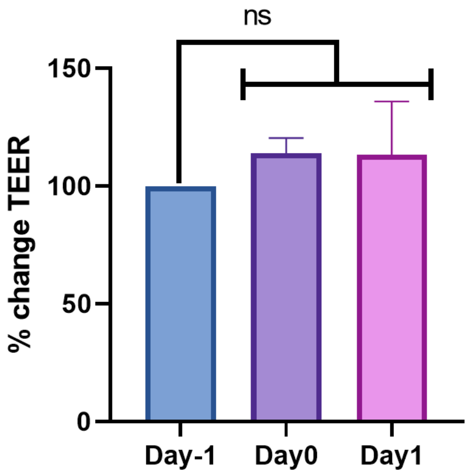

Figure S10. The percentage difference in TEER measurements. The TEER values were measured on Day-1 (one day before the liposomal treatment), Day 0 (the day of the liposomal treatment), and Day 1 (24 h after the liposomes treatment). The values of Day-1 were normalized to 100%, and the values of Day 0 and Day 1 were normalized to Day-1. The results demonstrated that the liposome treatment does not affect the TEER values (non-significant), indicating that the integrity of the endothelial layer was not damaged. The results (1 independent experimental repetition performed in 2 replicates) are presented as mean±standard deviation (SD). One-way ANOVA was used for the statistical analysis with multiple comparisons test adjusted p-value.

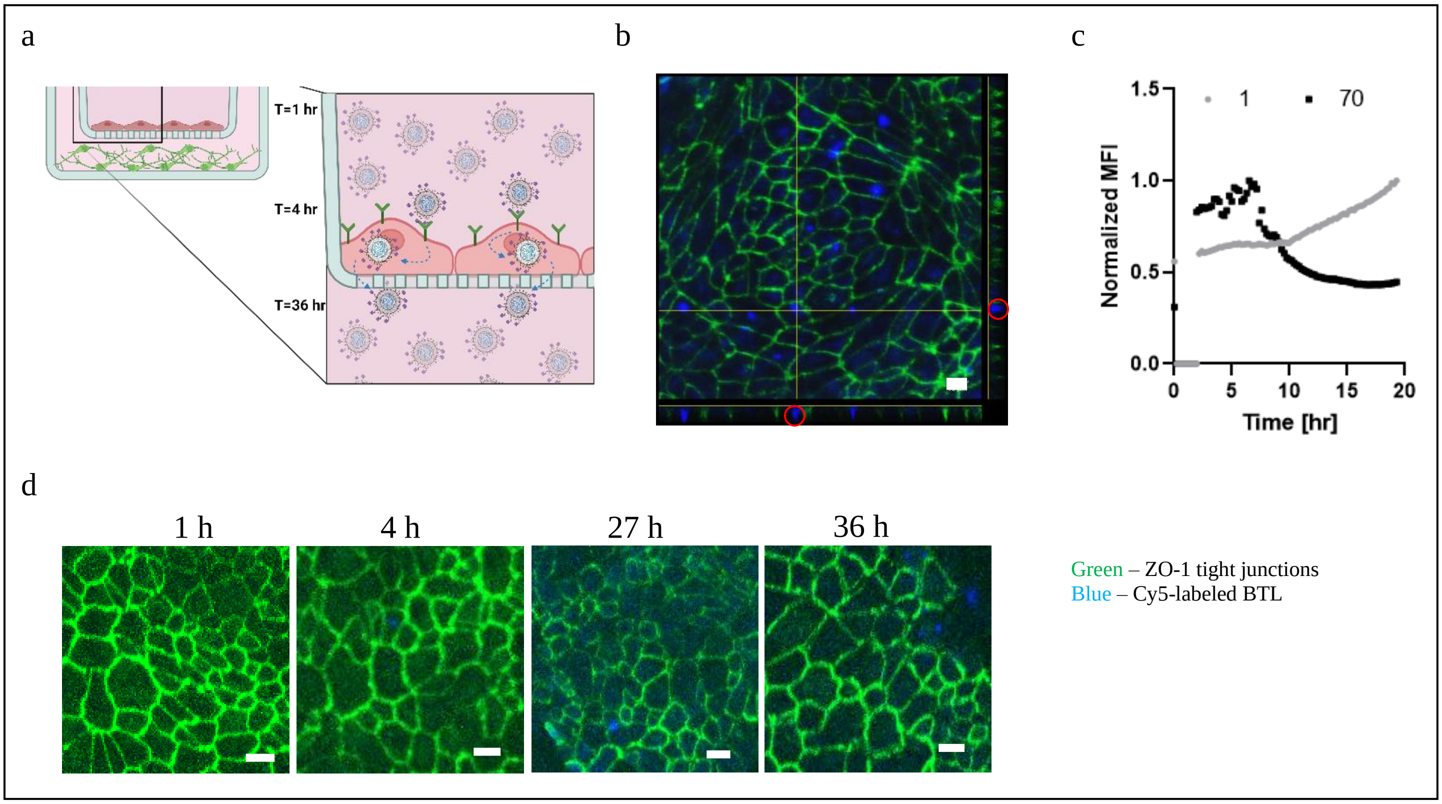

Figure S11. BTL cross the BBB in an *in vitro* model. (a) Schematic illustration showing how BTL cross the BBB layers over time. **(b)** Orthogonal view of BMEC incubated with liposomes for 24 h. BMECs were stained with ZO-1 antibody (tight junctions) and the BTL were labeled by Cy5 dye (scale bar: 10 µm); red circles indicate the presence of BTL particles inside the cells. **(c)** Normalized mean fluorescent intensity (MFI) of BTL at various heights of the membrane on which BMECs were cultured, as measured by live imaging. In this graph, Z=1 represents the lowest level below the cells, and Z=70 is the highest level above the cells. **(d)** Fluorescence images of BMECs stained for ZO-1 tight junctions and incubated with Cy5-labeled BTL for 1, 4, 27, 36 h; scale bar: 20 µm.

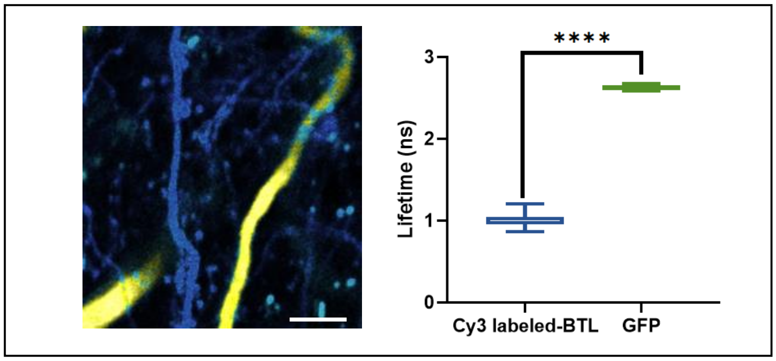

Figure S12. The uptake and distribution of BTL in neuronal brain cells of mice. *In-vivo* 2pFLIM pseudo-colored image of the GFP-labeled neuronal process and blood vessels following Cy3-labeled BTL (scale bar: 50 µm); the graph displays 2pFLIM measurements before and after the intravenous injection of Cy3-labeled BTL particles, revealing the GFP lifetime and the presence of Cy3-positive blood vessels. The clear differentiation in spectral characteristics and fluorescence lifetimes between GFP (2.62 ns±0.01 ns) and Cy3 (1.003 ns±0.01 ns) enables the accurate identification and tracking of the specific entry points and positions of Cy3-labeled BTL particles within GFP-labeled neurons ($p<0.0001$); this result ensures the fluorescence lifetime of GFP-labeled neurons can be effectively distinguished from the Cy3-labeled BTL within the brain's blood vessels. Results of a (14 independent repetitions performed) are displayed as mean±standard deviation (SD). Two-tailed unpaired Student's t-test was used for the statistical analysis of a, with multiple comparisons test adjusted *P*-value; ****$p<0.0001$.

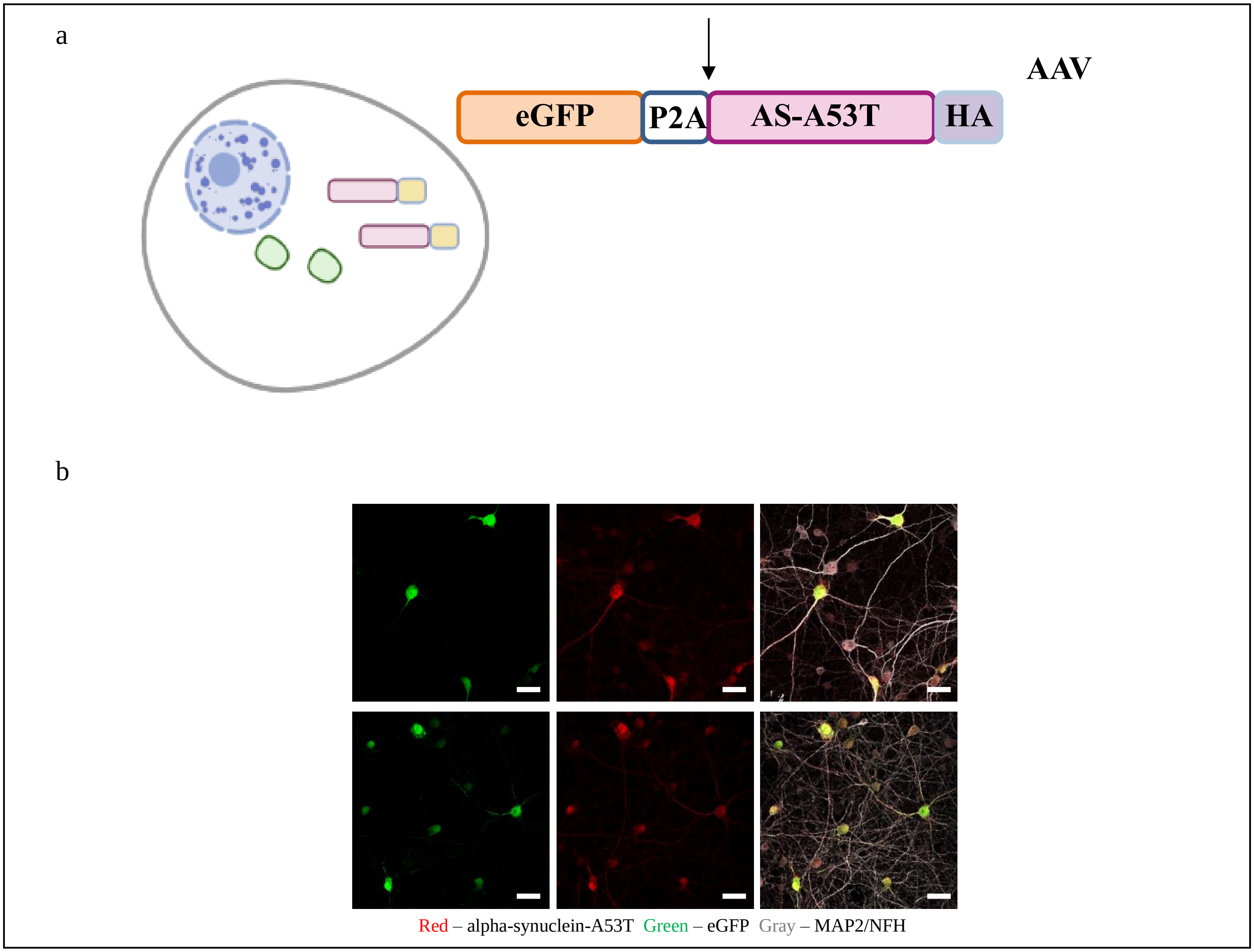

Figure S13. Morphological characterization of human alpha-synuclein-A53T overexpression in primary murine cortical neurons with the AAV P2A system. **(a)** Schematic representation of the AAV P2A system for dual expression of eGFP and alpha-synuclein with familial PD mutation A53T (alpha-synuclein A53T) under a human synapsin promoter from one mRNA. This system allows the robust expression of the eGFP for monitoring live transduction efficiency *in vitro* and *in vivo* without the need to conjugate the fluorescent protein directly to alpha-synuclein-A53T. Instead, the alpha-synuclein-A53T is tagged with a short peptide (HA), which allows the physiological conformation of the aggregate-prone protein. **(b)** Mouse primary cortical neurons were transduced with the AAV system and neurons were visualized for eGFP (green), stained for alpha-synuclein-A53T (targeting the HA tag; red), and for dendrites (MAP2; gray) or both dendrites and axons (NFH; gray). Images show alpha-synuclein-A53T expression both in the cell body and neurites; scale bar: 10 µm.

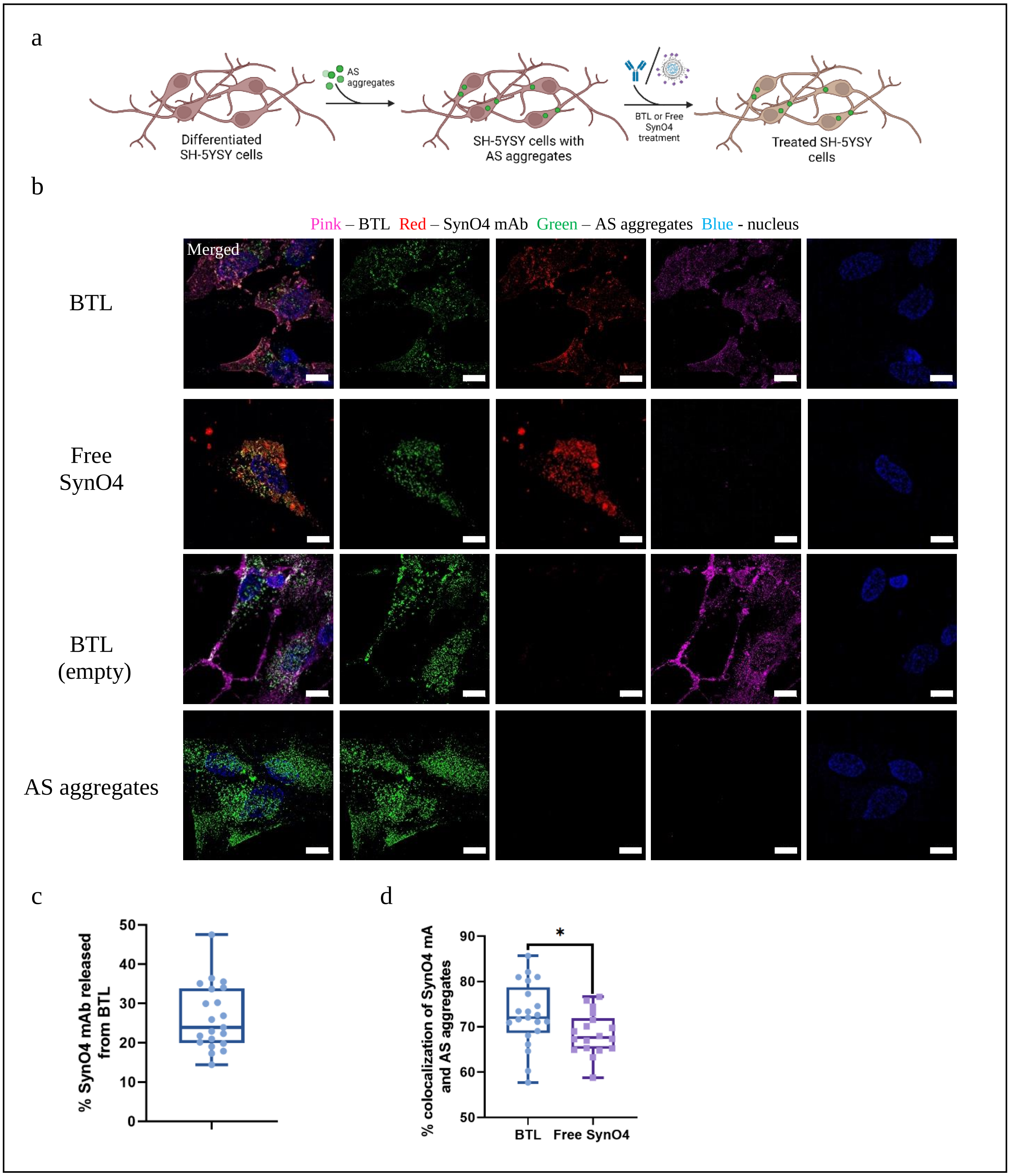

Figure S14. BTL uptake by PD-SH-SY5Y cells. (a) Schematic representation of PD-SH-SY5Y cells. AS aggregates were seeded with differentiated SH-SY5Y cells and after 6 h, the cells were washed and incubated with free SynO4 mAbs or BTL overnight. **(b)** Super-resolution images showing the cellular uptake of BTL compared to free SynO4 mAbs (scale bar: 10 µm). The merged images (left column) show a co-localization (yellow) between the mAbs (red) and AS aggregates (green) after the treatment with BTL (top row); BTL (empty) and AS aggregates were used as negative control **(c)** The percent of SynO4 mAbs co-localized with AS aggregates in PD-SH-SY5Y cells following treatment with BTL or free SynO4 mAbs (73%±7% vs. 68%±5%, respectively) **(d)** The percent of SynO4 mAbs released from BTL in PD-SH-SY5Y cell culture overnight (~25%). The graphs' results (1 independent experimental repetition performed in more than 20 technical replicates) are presented as mean±standard deviation (SD). One-way ANOVA was used for the statistical analysis with a multiple comparison test adjusted p value; $*P = 0.0346$.

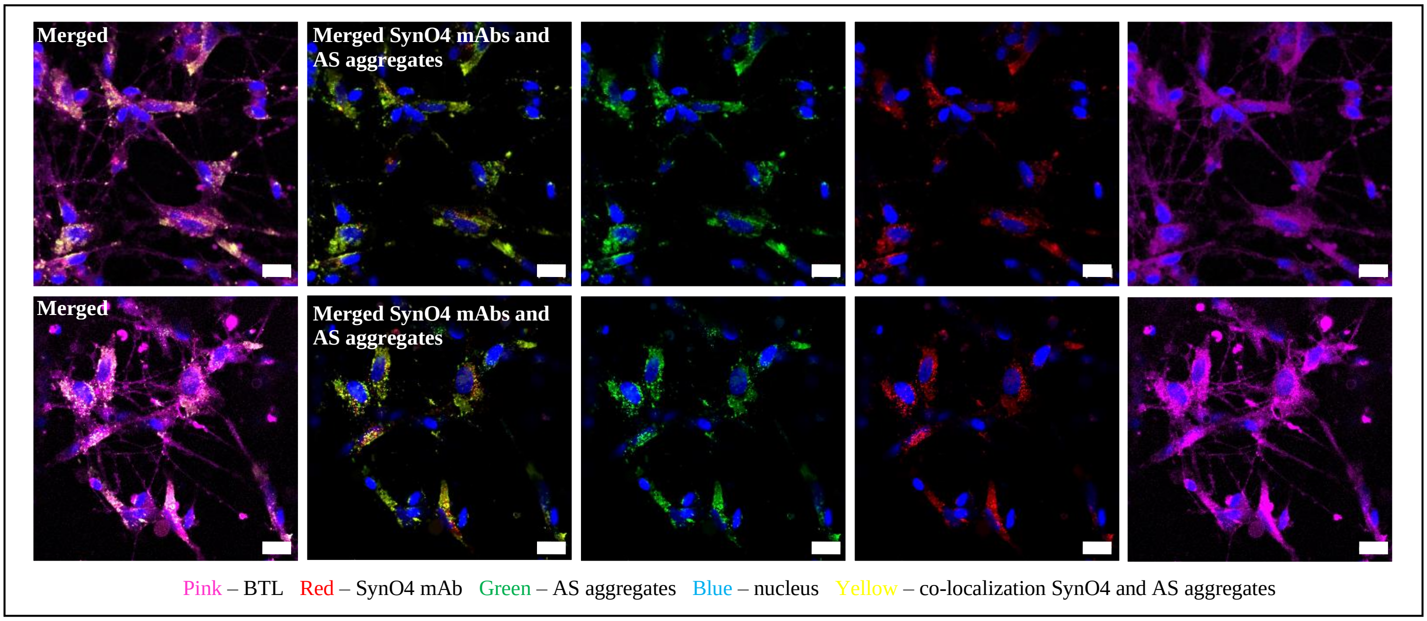

Figure S15. Confocal imaging of BTL uptake in PD-SH-SY5Y cells. The images depict the uptake of BTL (pink) within the cytoplasm and exons of the cells. The merged images illustrate co-localization (identified in the yellow channel) between SynO4 mAbs (red) and AS aggregates (green) within specific cellular locations; scale bar: 20 µm.

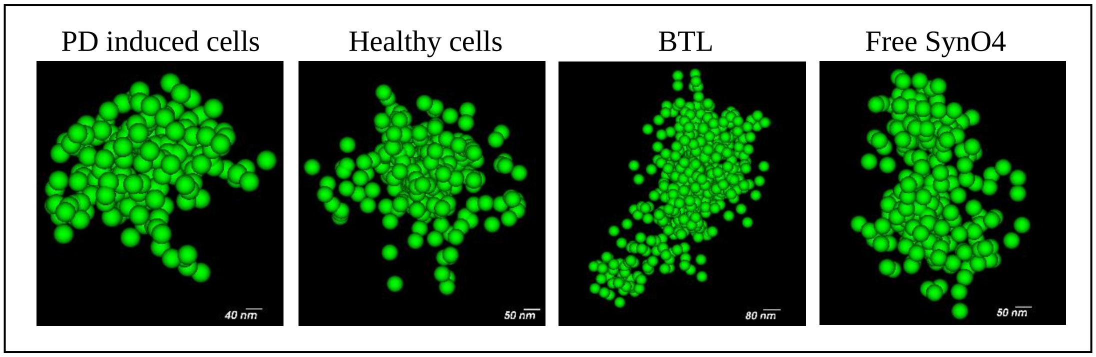

Figure S16. Zoom-in imaging of AS clusters in the different groups. The super-resolution images exhibit improved resolution and visualization of the AS structures, aiding in the identification of individual AS molecules.

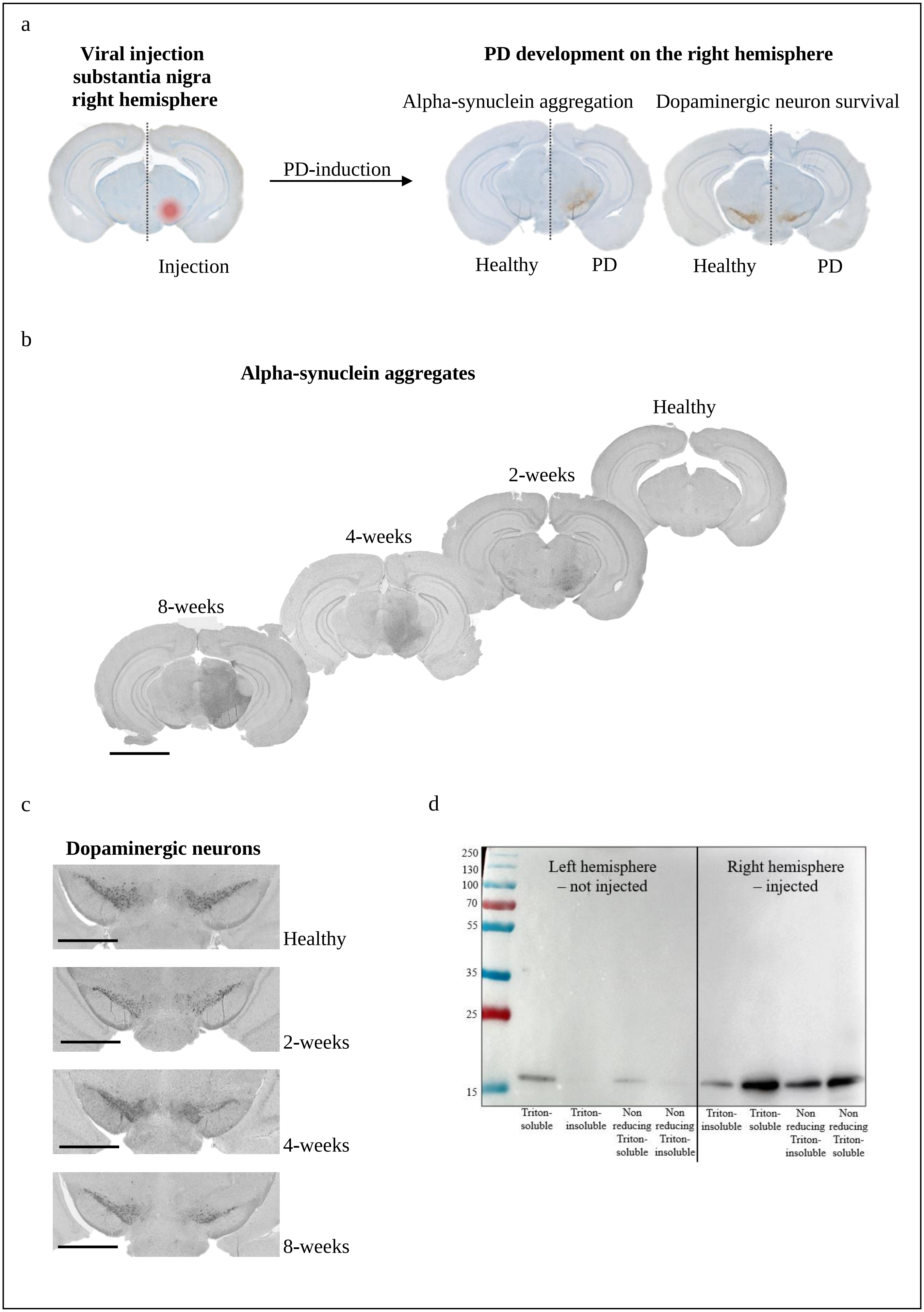
a
Viral injection substantia nigra right hemisphere
Injection
PD-induction
PD development on the right hemisphere
Alpha-synuclein aggregation
Dopaminergic neuron survival
Healthy
PD
Healthy
PD
b
Alpha-synuclein aggregates
Healthy
2-weeks
4-weeks
8-weeks
c
Dopaminergic neurons
Healthy
2-weeks
4-weeks
8-weeks
d
250
130
100
70
55
35
25
15
Left hemisphere – not injected
Right hemisphere – injected
Triton-soluble
Triton-insoluble
Non reducing Triton-soluble
Non reducing Triton-insoluble
Triton-insoluble
Triton-soluble
Non reducing Triton-insoluble
Non reducing Triton-soluble

Figure S17. Viral PD mice model establishment. (a) Illustration of the experiment: Healthy mice received unilateral AAV injection encoding human alpha-synuclein (AS) in the right hemisphere. The brains were harvested, cut for histology, and stained against alpha-synuclein aggregates and dopaminergic neurons. **(b-c)** The amount of (b) AS aggregates and (c) dopaminergic neurons in mice sacrificed, 2, 4, and 8 weeks post viral injection compared to healthy mice not-injected with the viral vector. Over time, the production of alpha-synuclein aggregates increases (scale bar: 2000 µm) and the number of dopaminergic neurons decreases on the viral injected hemisphere (scale bar: 2000 µm). (**d)** Western blot analysis of the protein extract under various conditions (Triton-soluble, Triton-insoluble, non-reducing Triton-insoluble, non-reducing Triton-soluble) in the right and the left hemisphere of A PD brain 2 weeks after viral injection. The AS protein was stained using an anti-AS mAb. Strong staining of the AS protein (17 kDa) in the right brain extract, compared to almost no expression in the left hemisphere.

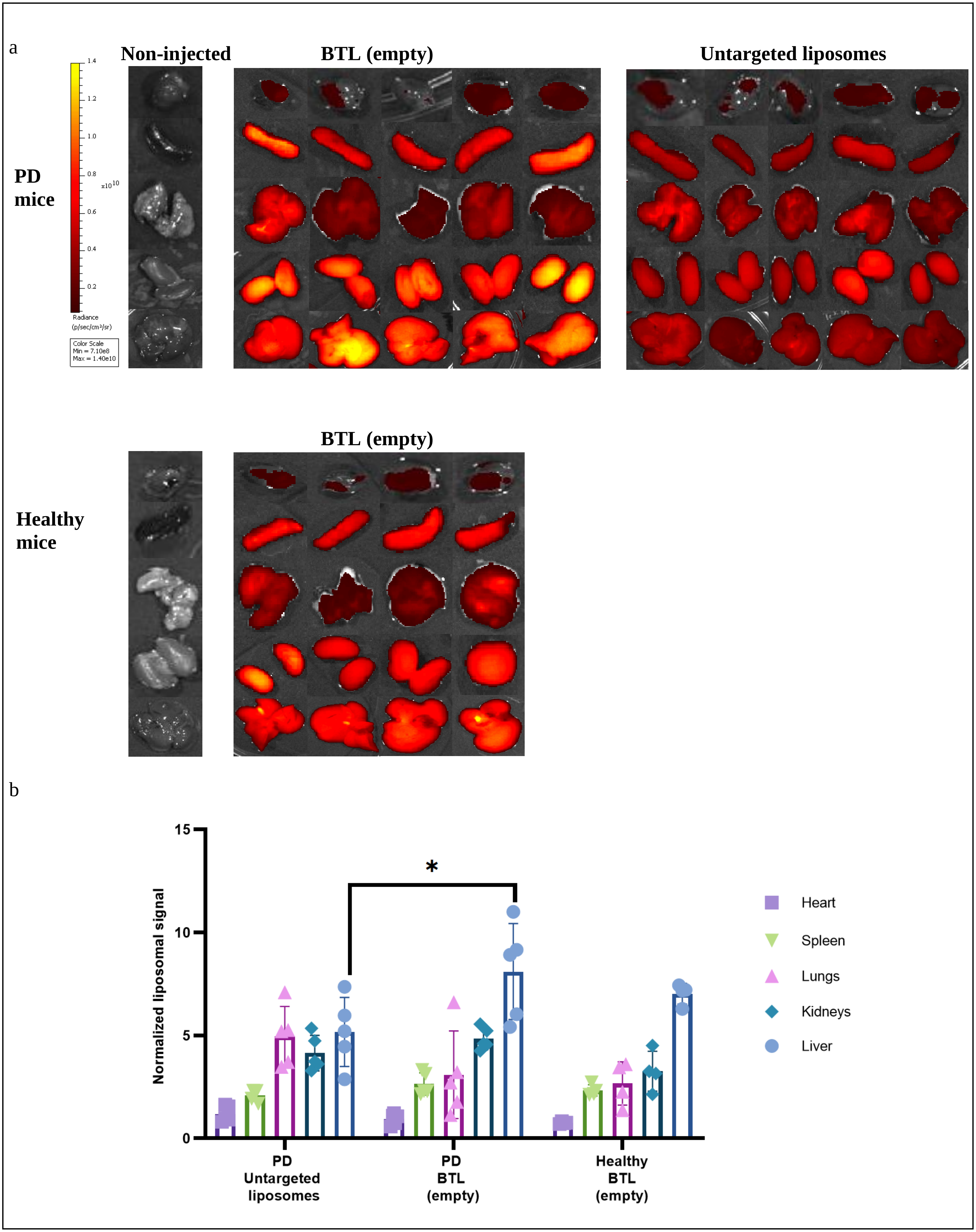

a
Non-injected
BTL (empty)
Untargeted liposomes
PD mice
Healthy mice
BTL (empty)
1.4
1.2
1.0
0.8
0.6
0.4
0.2
x10^10
Radiance (p/sec/cm²/sr)
Color Scale
Min = 7.10e8
Max = 1.40e10
b
Normalized liposomal signal
15
10
5
0
*
PD Untargeted liposomes
PD BTL (empty)
Healthy BTL (empty)
Heart
Spleen
Lungs
Kidneys
Liver

Figure S18. Biodistribution of BTL and untargeted liposomes in PD and healthy mice. (a) Representative IVIS *ex-vivo* images of the accumulation of BTL (empty) and untargeted liposomes, both labeled with Cy5 dye, in different organs (from top to bottom: heart, spleen, lungs, kidneys, and liver) 24 h after being intravenously injected into PD-induced mice (8 weeks post viral injection) and healthy mice **(b)** Quantification of liposome accumulation using IVIS software imaging. The results (5 independent experimental repetitions) are presented as mean±standard deviation (SD). A 2-way ANOVA test was used for the statistical analysis with a multiple comparison test adjusted p value; *p=0.0187.

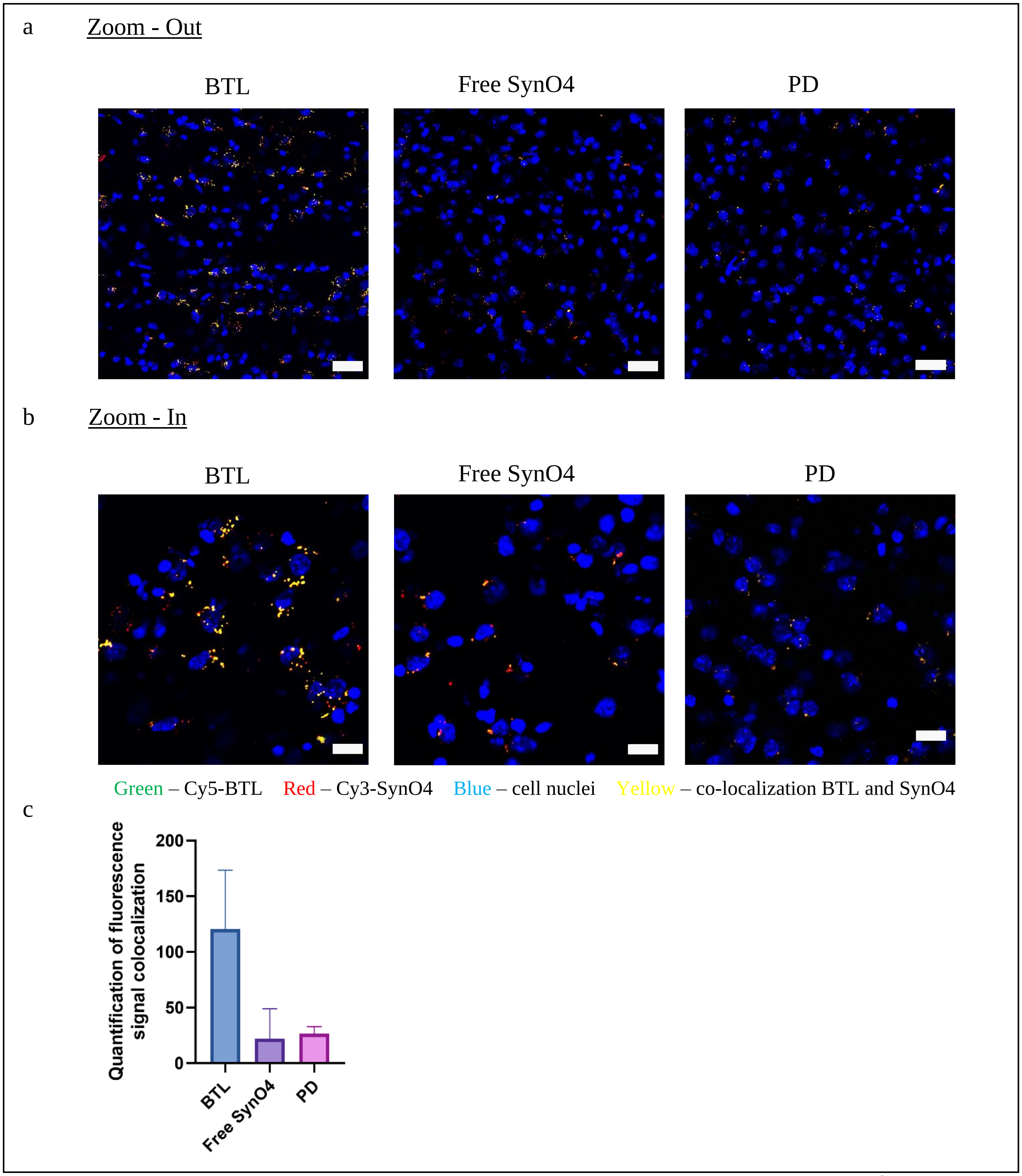

Figure S19. BTL detection in PD-induced brain. (a) Fluorescent histology images of the substantia nigra 24 h after the intravenous injection of BTL or free SynO4 mAbs, or without treatment injection (PD). The SynO4 antibodies are labeled with Cy3 (red), BTL with Cy5 (green), and the nuclei are stained with DAPI (blue) (scale bar: 20 µm). The co-localization of Cy3-SynO4 and Cy5-BTL is shown as a yellow signal. (**b)** Zoom-in images of the top panel X63 magnification (scale bar: 10 µm). **(c)** Quantification of fluorescence signal co-localization of Cy3-SynO4 and Cy5-BTL in brain sections. The results of c (3 independent repetitions performed) are displayed as mean±standard deviation (SD). One-way ANOVA was used for statistical analysis; p=0.1122 of BTL vs. free SynO4, p=0.1268 of BTL vs. PD, and p=0.1268 of free SynO4 vs. PD.

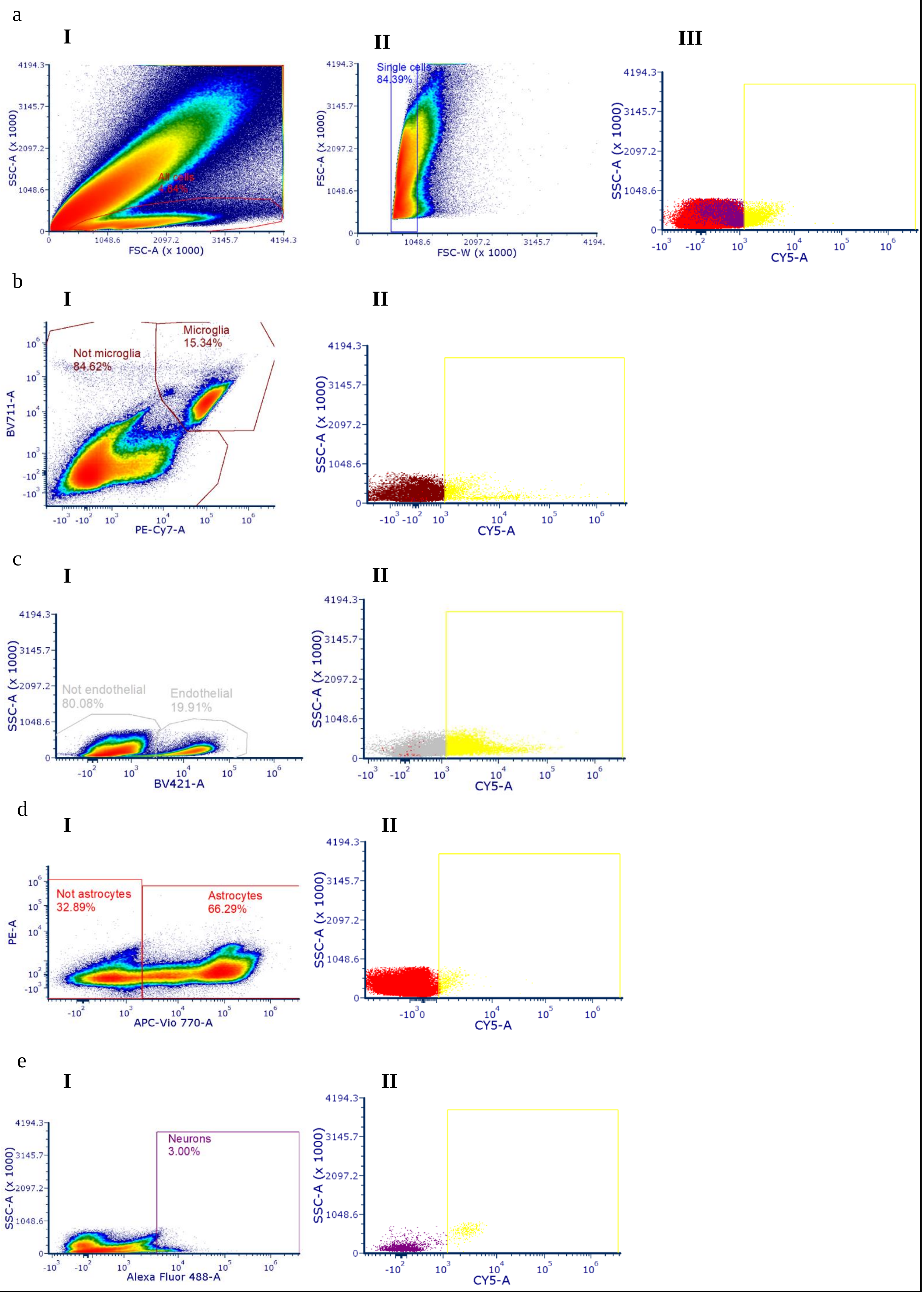
a
I
II
III
All cells
4.64%
Single cells
84.39%
SSC-A (x 1000)
FSC-A (x 1000)
FSC-W (x 1000)
CY5-A
b
I
II
Not microglia
84.62%
Microglia
15.34%
BV711-A
PE-Cy7-A
c
I
II
Not endothelial
80.08%
Endothelial
19.91%
BV421-A
d
I
II
Not astrocytes
32.89%
Astrocytes
66.29%
PE-A
APC-Vio 770-A
e
I
II
Neurons
3.00%
Alexa Fluor 488-A

Figure S20. Gating strategy for flow cytometry assay. (a-e) The gating strategy to discriminate brain cell types (microglia, endothelial, astrocytes, and neurons) and to detect the Cy5-positive population in each cell type is shown on a representative liposome PD-brain sample. The all-cell population is detected by plotting the forward scatter-area against the side scatter-area (plot a, I); cells are separated from debris (red). Single cells are identified by plotting the forward scatter-width against the forward scatter-area (plot a, II). The stained Cy5 single-cell events are presented in Plot A, III. The microglia are eliminated by gating on BV711(CD45)-positive and pe-Cy7(CD11b)-positive events (plot b, I); the stained Cy5 microglia cells are identified by plot b, II. Then, the endothelial cells are eliminated by gating on BV421(CD31)-positive events (plot c, I); the stained Cy5 endothelial cells are identified by plot C, II. Next, the astrocytes are eliminated by gating PE(CD44)-positive and APC-Vio770(ACSA-2)-positive events (plot d, I); the stained Cy5 astrocytes are identified by plot d, II. Finally, the neurons are eliminated by gating on Alexa Fluor 488(CD24)-positive events (plot e, I); the stained Cy5 neurons are identified by plot e, II.

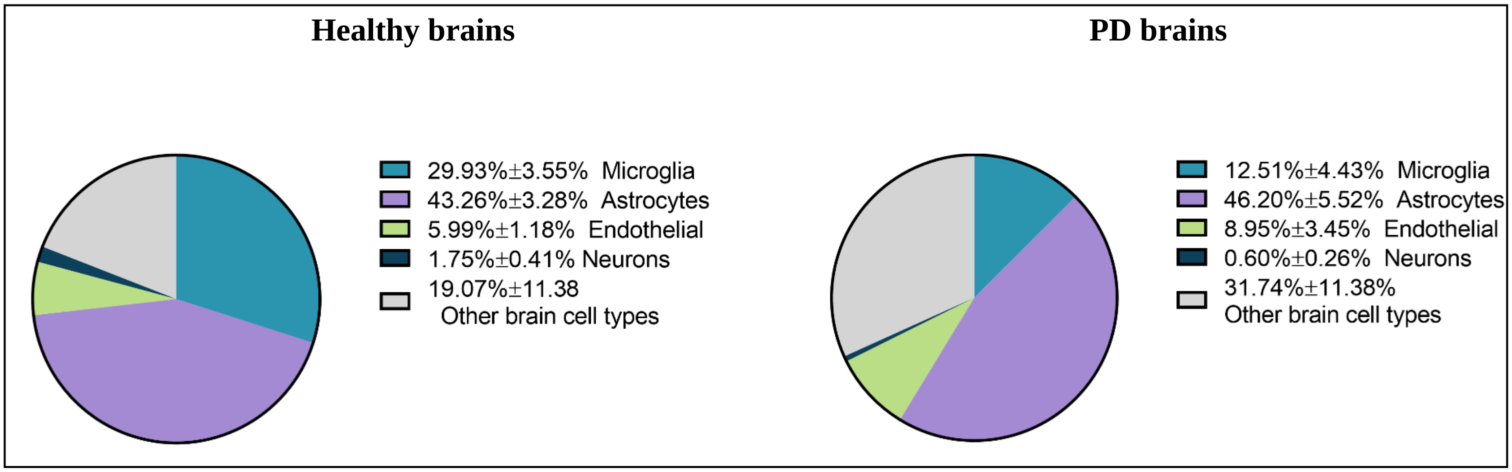

Figure S21. Brain cell population characterization. Brain cell samples were stained with a panel of antibodies and then measured by flow cytometry. Each cell type (microglia, endothelial cells, astrocytes, or neurons) is identified by its markers from the single-cell population gating. The results are shown by pie chart graphs (4-10 independent experimental brain repetitions) and presented as mean±standard deviation (SD).

a

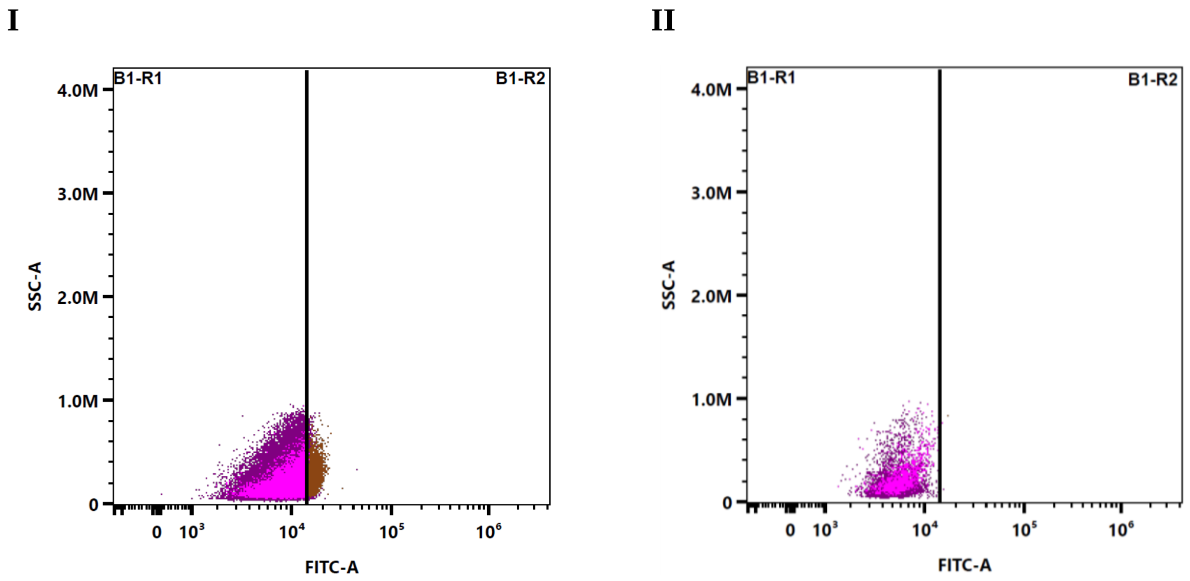

b

Gate	Count	FITC-MFI	SD-MFI	% Parent
I: B1-R1	175,367	8,846	2,451	96.76
I: B1-R2	5,869	15,707	1,241	3.24
II: B1-R1	6142	5,489	1,673	99.97
II: B1-R2	2	-	-	0.03

c

Gate	Count	Cy5-MFI	SD-MFI	Cy3-MFI	SD-MFI	% Parent
I: B1-R1	175,367	3,200	1,514	7,835	5,669	96.76
I: B1-R2	5,869	6,222	2,154	17,840	9,737	3.24

Figure S22. BTL uptake in PD dopaminergic neurons of mice. The gating strategy to discriminate microglia, endothelial cells, and astrocytes is shown in Figure S20. **(a)** The dopaminergic neurons are eliminated by gating on FITC (Dopamine Transporter)-positive events (B1-R2 gate) (I); a plot of the negative control of a BTL PD-brain sample not stained with FITC (Dopamine Transporter) antibody (II). **(b)** Data table showing the number of cells, FITC-MFI, SD-MFI, and % parent in the negative (B1-R1; other neuron subtypes or oligodendrocytes) and positive (B1-R2; dopaminergic neurons) gates of plots I and II in a. **(c)** Data table showing the number of cells, Cy5-MFI, Cy3-MFI, SD-MFI, and % parent in the B1-R1 and B1-R2 gates of plot I in a.

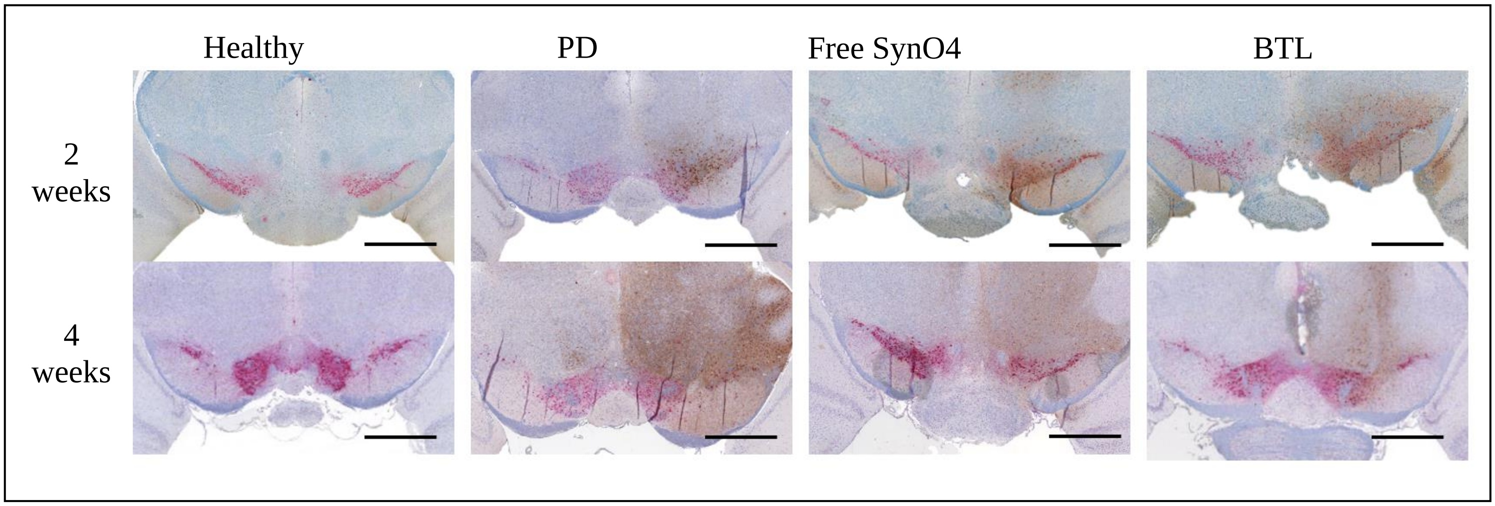

Figure S23. Co-localization of dopaminergic neurons and aggregated alpha-synuclein in the substantia nigra of a mouse brain. Immunohistochemical analysis of representative brain sections 2 and 4 weeks post viral injection compared to healthy mice not injected with the viral vector. The sections were double-stained for dopaminergic neurons (anti-tyrosine hydroxylase, pink) and aggregated alpha-synuclein (5G4 clone, brown) (scale bar: 2000 μm).

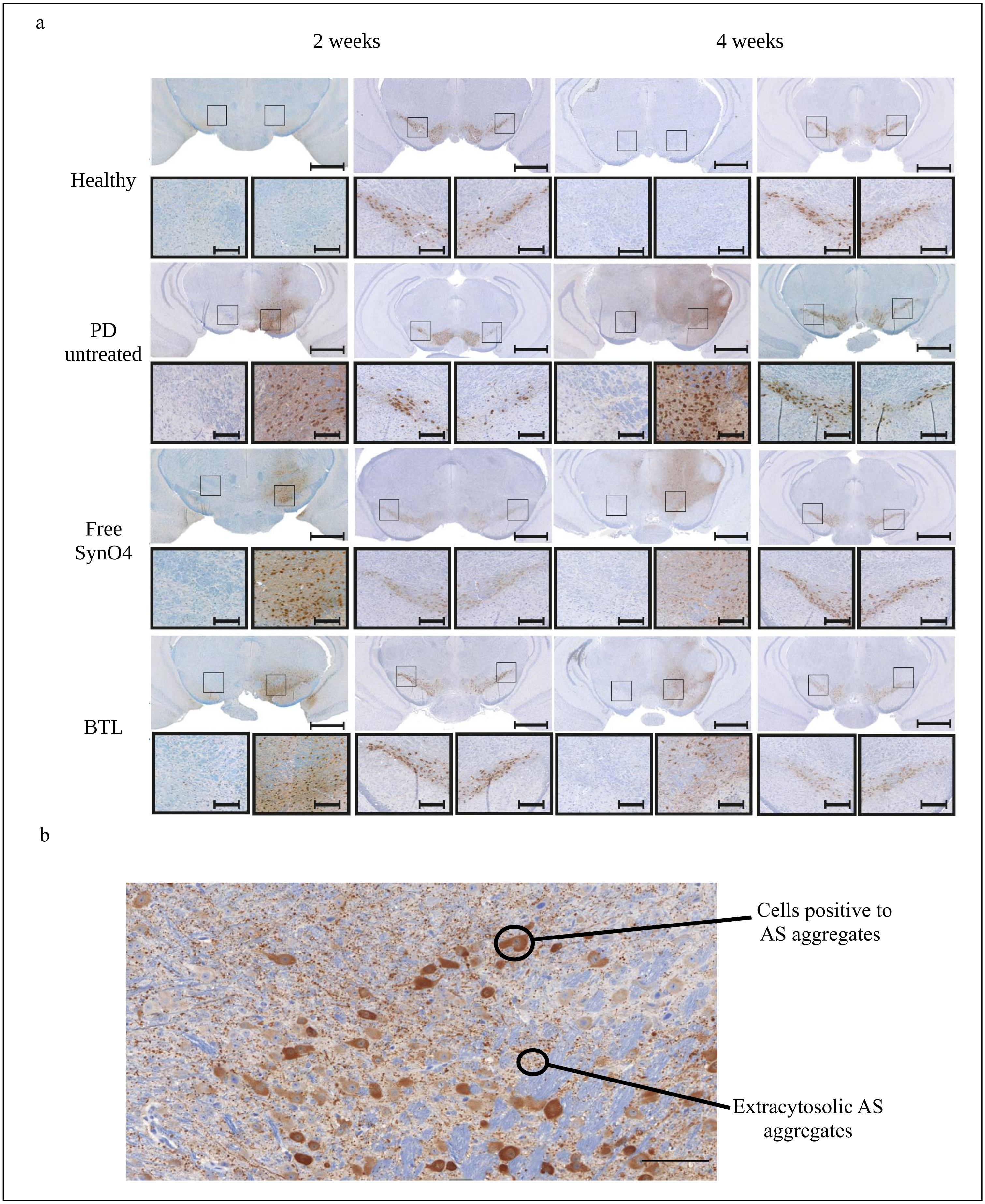
a
2 weeks
4 weeks
Healthy
PD
untreated
Free
SynO4
BTL
b
Cells positive to
AS aggregates
Extracytosolic AS
aggregates

Figure S24. Histological staining of AS aggregates and dopaminergic neurons in brain sections following efficacy experiment evaluation. (a) Representative images of histological substantia nigra area sections after two and four weeks of the respective treatments. The sections were stained against aggregated alpha-synuclein (5G4 marker) and dopaminergic neurons (TH marker) (scale bar: 2000 µm); the zoom-in images (lower panels) are of the black squares in the images (scale bar: 200 µm). (**b)** A representative stained cut image of AS aggregates is presented to emphasize image analysis processing. A color segmentation technique was employed to count the extracytosolic AS aggregates present in the brown-colored areas, which were then compared to the overall tissue area. Additionally, the co-localization of brown color expression and cell structure was used to identify and count the cells that were positive and expressed intracellular AS aggregates. (Scale bar: 100 µm).

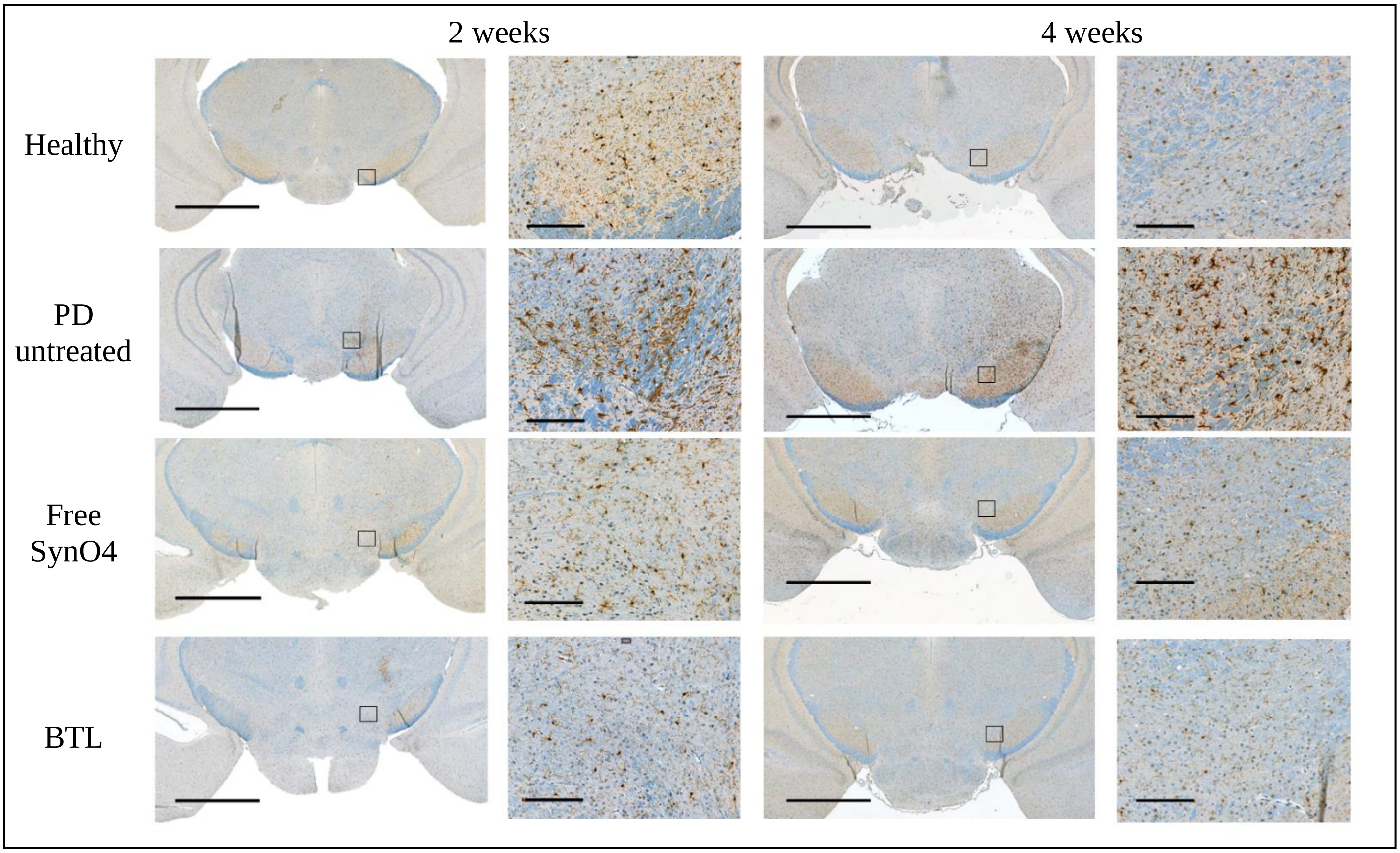

Figure S25. Histological staining of activated microglia cells in brain sections following efficacy evaluation. Representative images of histological substantia nigra area sections after two and four weeks of the respective treatments. The sections were stained against activated microglia cells (Iba1 marker) (scale bar: 1000 µm); the zoom-in images (right panels) are of the black squares in the images (scale bar: 200 µm).

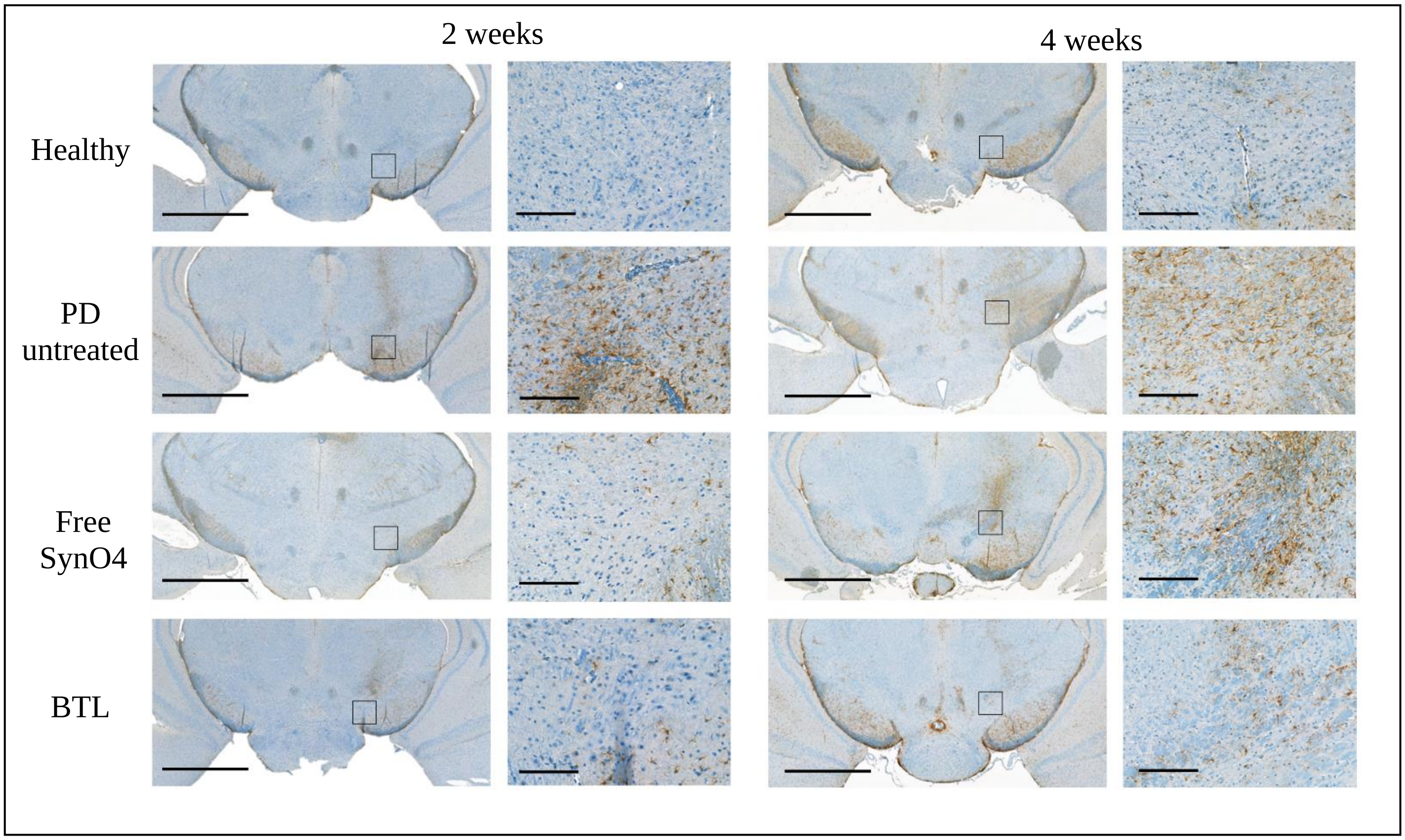

Figure S26. Histological staining of reactive astrocyte cells in brain sections following efficacy evaluation. Representative images of histological sections of the substantia nigra after two and four weeks of the respective treatments. The sections were stained against reactive astrocyte cells (GFAP marker) (scale bar: 1000 µm); the zoom-in images (right panels) are of the black squares in the images (scale bar: 200 µm).

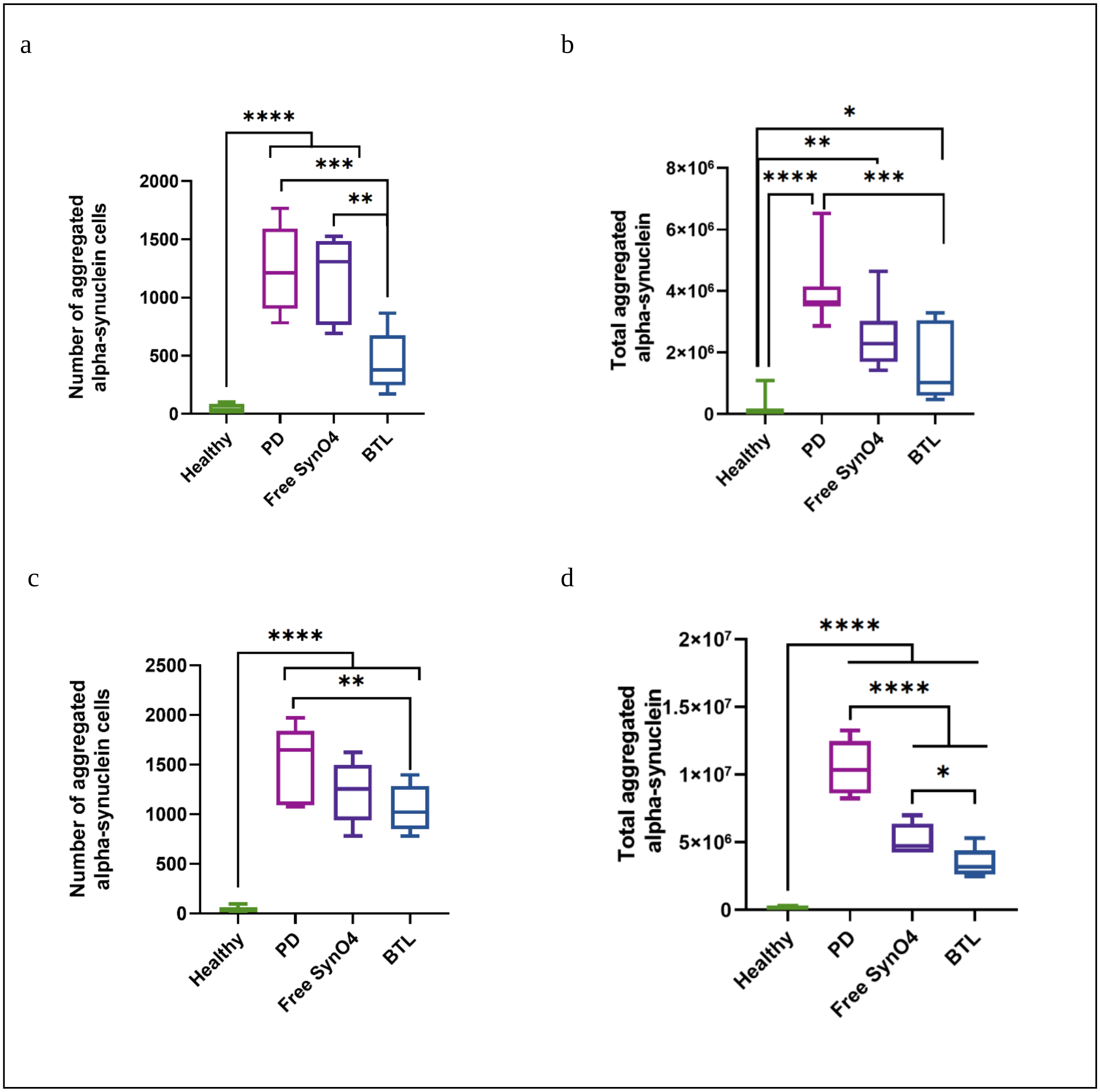

Figure S27. BTLs' capacity to reduce alpha-synuclein aggregation in the PD mice model. (**a)** Quantification of aggregated alpha-synuclein-positive cells in substantia nigra brain sections after 2 weeks of treatment with BTL or free SynO4 (**b)** Quantification of the total aggregated alpha-synuclein in substantia nigra brain sections after 2 weeks of treatment with BTL or free SynO4 (**c)** Quantification of aggregated alpha-synuclein-positive cells in substantia nigra brain sections after 4 weeks of treatment with BTL or free SynO4 (**d)** Quantification of the total aggregated alpha-synuclein in substantia nigra brain sections after 4 weeks of treatment with BTL or free SynO4. The results of a and b (3-5 independent repetitions performed in 1-3 technical replicates) and of c and d (4-5 independent repetitions performed in 1-3 technical replicates) are displayed as a min to max box graph and presented as mean±standard deviation (SD). One-way ANOVA was used for statistical analysis. *p<0.05, **p<0.01, ***p<0.001, ****p<0.0001.

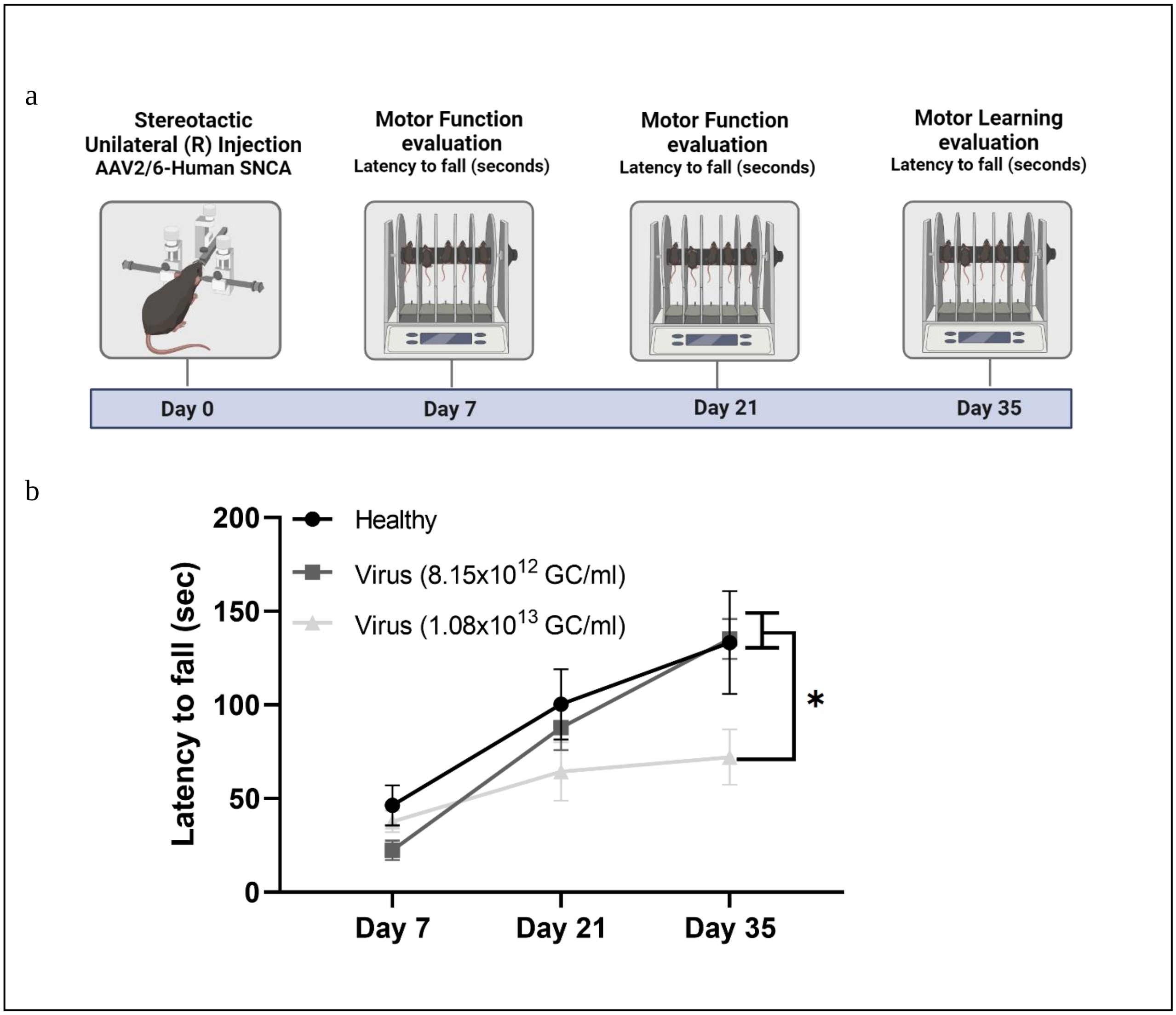

Figure S28. Behavioral viral PD mice model establishment. (a) Illustration of the behavioral evaluation experiment involving healthy mice (n=5) and PD mice that received a unilateral AAV injection encoding human AS at one of two different concentrations, 8.15×10^{12} GC/ml (n=5) or 1.08×10^{13} GC/ml (n=5). **(b)** Latency to fall, measured in an accelerating speed rotarod, of the three groups at 7, 21, and 35 days after viral injection. Both the healthy group and the low viral concentration injection group showed significant performance improvement from day 7 to day 35 ($p<0.0006$), while the high viral concentration group did not exhibit significant improvement, indicating their limited motor learning capacity ($p<0.2457$). The results shown are of 5 independent repetitions and are presented as mean±standard deviation (SD). A 2-way ANOVA test was used for the statistical analysis with a multiple comparison test adjusted p-value; $^*p \leq 0.016$.

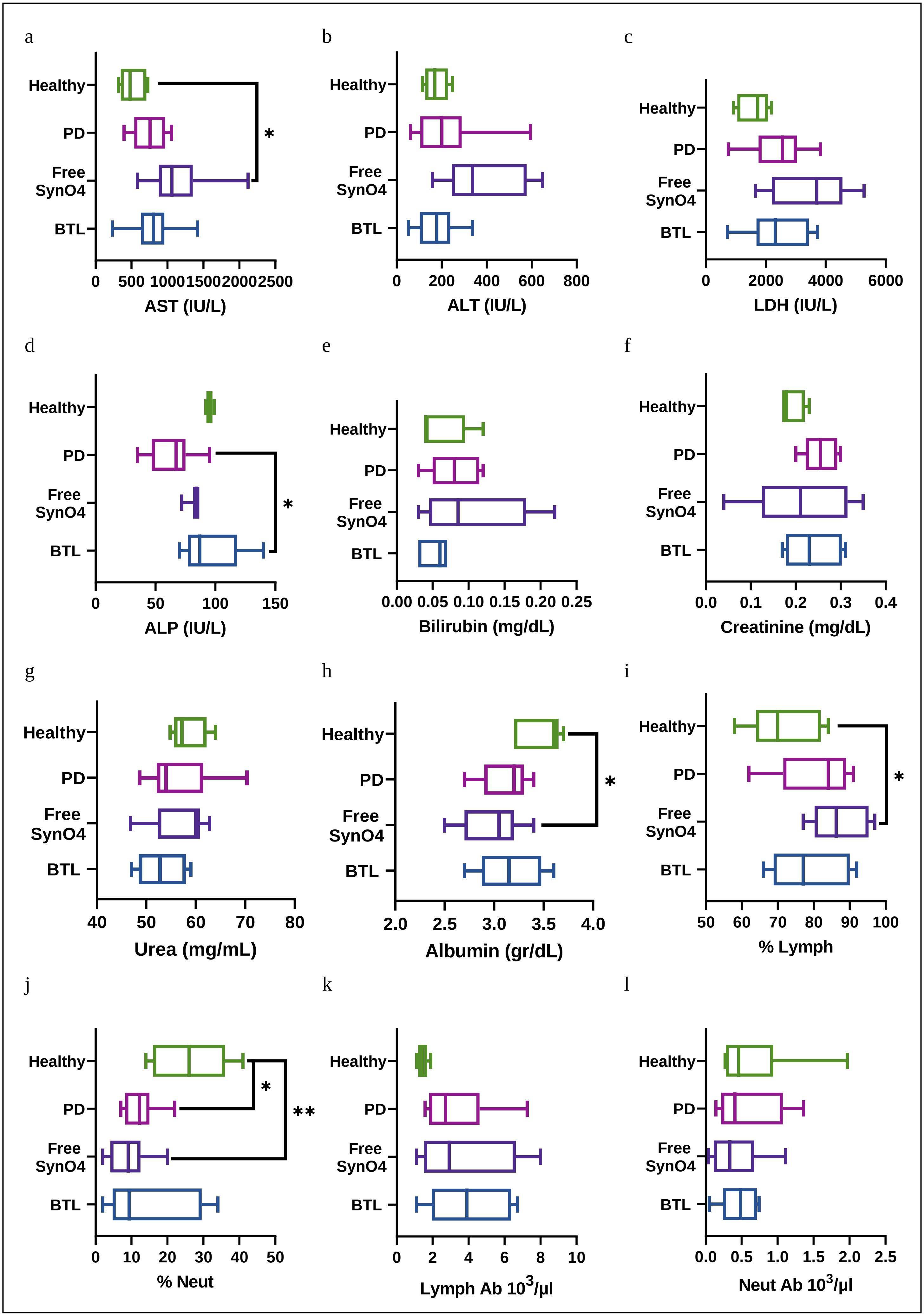

a
Healthy
PD
Free SynO4
BTL
*
0 500 1000 1500 2000 2500
AST (IU/L)
b
Healthy
PD
Free SynO4
BTL
0 200 400 600 800
ALT (IU/L)
c
Healthy
PD
Free SynO4
BTL
0 2000 4000 6000
LDH (IU/L)
d
Healthy
PD
Free SynO4
BTL
*
0 50 100 150
ALP (IU/L)
e
Healthy
PD
Free SynO4
BTL
0.00 0.05 0.10 0.15 0.20 0.25
Bilirubin (mg/dL)
f
Healthy
PD
Free SynO4
BTL
0.0 0.1 0.2 0.3 0.4
Creatinine (mg/dL)
g
Healthy
PD
Free SynO4
BTL
40 50 60 70 80
Urea (mg/mL)
h
Healthy
PD
Free SynO4
BTL
*
2.0 2.5 3.0 3.5 4.0
Albumin (gr/dL)
i
Healthy
PD
Free SynO4
BTL
*
50 60 70 80 90 100
% Lymph
j
Healthy
PD
Free SynO4
BTL
*
**
0 10 20 30 40 50
% Neut
k
Healthy
PD
Free SynO4
BTL
0 2 4 6 8 10
Lymph Ab 10^3/μl
l
Healthy
PD
Free SynO4
BTL
0.0 0.5 1.0 1.5 2.0 2.5
Neut Ab 10^3/μl

Figure S29. BTL toxicity evaluation in viral PD mice model. (a-e) Hepatotoxicity test of blood on day 40 of the experiment. Hepatic enzymes: (a) Aspartate transaminase (AST), (b) Alanine transaminase (ALT), (c) Lactate dehydrogenase (LDH), (d) Alkaline phosphatase (ALP), and (e) total bilirubin (Bilirubin) were measured. No significant differences were found between the healthy and BTL groups. As a result of the free SynO4 treatment, hepatic enzymes as well as bilirubin levels were increased, indicating liver damage. **(f-h)** Nephrotoxicity test of blood on day 40 of the experiment. (f) Creatinine, (g) urea, and (h) albumin levels were measured. No differences were noted between the healthy and BTL groups. **(i-l)** White blood cell count (WBC) test of blood collected on day 40 of the experiment. (i) %Lymph (% Lymphocytes of the total WBC), (j) %Neut (% neutrophils of total WBC), (k) Lymph Ab (lymphocyte antibodies), and (l) Neut Ab (neutrophil antibodies). A higher presence of lymphocytes and their antibodies was noted in the PD, free SynO4, and BTL groups. In the free SynO4 group, lower levels of neutrophils were observed. All results (7-8 independent repetitions) are presented as mean±standard deviation (SD). One-way ANOVA was used for statistical analysis; $*p \leq 0.0379$, $**p = 0.0034$.

Movie S1. Protein dynamics crystal structure of holo-transferrin human protein. Color legend: TF - green, aspartic acid amino acid - pink, glutamic acid amino acid - red, and the binding-site pocket to TfR1 – blue.

Transferrin has a total of 678 amino acids, and in the movie, the acidic amino acids are labeled, as well as the binding pocket:

- Aspartic acid residues – 22, 43, 52, 66, 77, 82, 88, 109, 123, 157, 182, 185, 216, 220, 238, 240, 248, 255, 259, 280, 296, 311, 316, 329, 356, 375, 395, 406, 411, 435, 439, 457, 461, 496, 510, 548, 567, 577, 584, 611, 633, 647, 652, 653, 662.
- Glutamic acid residues – 32, 34, 75, 102, 108, 160, 166, 231, 243, 256, 279, 284, 291, 300, 337, 347, 352, 357, 370, 376, 386, 391, 394, 404, 429, 438, 442, 497, 501, 526, 531, 545, 575, 579, 591, 592, 613, 644, 667, 672, 673, 691.

Based on the protein dynamics structure, the TF molecule possesses 38 acidic binding sites to which the amino PEG in the BTL can bind.

- TF's binding site to TfR1 amino acid residues - 268, 114, 82, 240, 375, and 207.

Movie S2. Live imaging of BTL in BBB chip. Time lap movie (played at a speed of x0.5) showing BTL crossing BBB cells layers over 20 h.

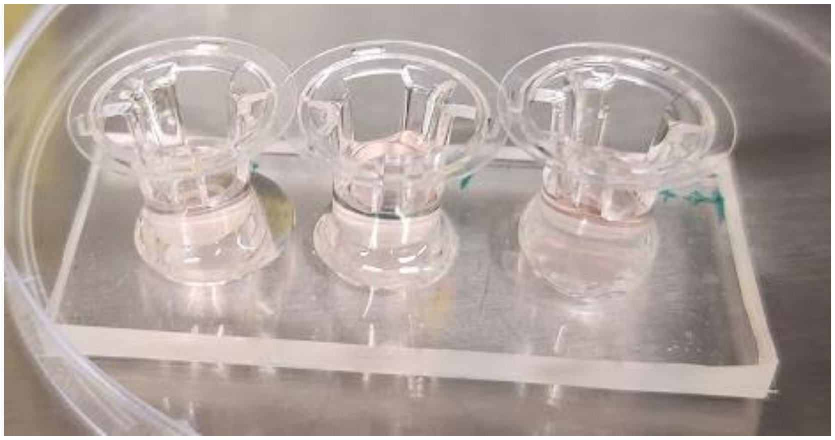

Here is an image showing the chip used to visualize the transport of BTL particles through the BBB.

Movie S3. A typical instance of the Rotor-rod test conducted 29 days after viral injection. The test was performed on a mouse selected from the healthy group, the untreated PD group, the free SynO4 group, and the BTL group. The recorded video showcases the mice's latency to fall, played at a speed of x4.0.
